# Supercharged ferritin nanocages enable universal cytosolic protein delivery

**DOI:** 10.1038/s41467-026-74247-x

**Published:** 2026-06-09

**Authors:** Dingkang Liu, Hong Luo, Qingzhou Lu, Lichao Yu, Minjiang Chen, Wenbing Yao, Pan Hu, Lubin Liu, Wei Liu, Xiangdong Gao, Jiansong Ji, Jun Yin

**Affiliations:** 1 Jiangsu Key Laboratory of Druggability of Biopharmaceuticals and State Key Laboratory of Natural Medicines, School of Life Science and Technology, China Pharmaceutical University, Nanjing, China; 2 Department of Obstetrics and Gynecology, Women and Children’s Hospital of Chongqing Medical University, Chongqing, China; 3 Zhejiang Key Laboratory of Imaging and Interventional Medicine, Zhejiang Engineering Research Center of Interventional Medicine Engineering and Biotechnology, The Fifth Affiliated Hospital of Wenzhou Medical University, Lishui, China

**Keywords:** Protein delivery, Nanoparticles, Nanobiotechnology

## Abstract

Efficient intracellular protein delivery represents an essential prerequisite for protein-based biotechnologies and therapeutics targeting intracellular components. However, this process is limited by multiple factors, including nonspecific protein binding, insufficient cellular uptake, inefficient endosomal escape, and inadequate cytosolic protein release. Here we show that by engineering fully recombinant supercharged protein nanocages, we achieve exceptionally high cellular uptake using a strategy we term ‘supercharged interface engineering’. By incorporating unnatural amino acids bearing phenylboronic acid groups, we develop a representative protein nanocage, pFn + . Simply mixing pFn+ with protein cargoes forms a noncovalent complex possessing enhanced cellular uptake efficiency, robust endosomal escape capability, and excellent biocompatibility. Notably, this system successfully delivers functional gene-editing tools and therapeutic antibodies in female mouse models. These findings indicate that pFn+ represents a promising platform for enhancing the cytosolic delivery of protein cargoes. Moreover, the proposed supercharged interface engineering strategy is valuable for advancing next-generation intracellular protein delivery systems.

## Abstract (web summary)

Protein therapeutics require efficient intracellular protein delivery. Here, the authors report on supercharged ferritin nanocages, loaded by simple mixing, which allow for cytosolic protein delivery through supercharged interface engineering, demonstrating application for gene editing and antibody delivery.

## Introduction

Proteins have emerged as indispensable tools in biological and pharmacological applications, owing to their high specificity and activity^1^. Despite significant success in targeting extracellular proteins, most protein-based diagnostics and drugs, often large and hydrophilic, face challenges in accessing intracellular targets due to inefficient delivery into cells^2^. This intracellular delivery hurdle poses a major challenge for the life sciences, hindering the development of protein-based therapeutics and diagnostics for intracellular targets.

Over the past decades, numerous approaches have been developed for cellular protein delivery, including cationic lipids^3^, synthetic polymers^4–6^, oligonucleotides^7^, cell-penetrating peptides (CPPs)^8^, and supercharged proteins^9,10^ or polypeptides^11^. Despite these advancements, several challenges persist—namely low potency, significant cytotoxicity, lack of generality, and endosomal entrapment^12^. Among these methods, CPPs have emerged as powerful tools for delivering various cargoes^13^. Cationic CPPs, typically composed of 8–10 arginine or lysine residues, represent the most widely used and well-characterized class. To enhance their efficacy for delivering larger protein cargos, researchers have proposed various strategies—such as peptide cyclization^14^—which likely optimizes the spatial presentation of positive charges. The observation that CPPs tend to form clusters prior to internalization, combined with recent insights into the improved efficacy of cyclic and tricyclic CPPs, supports the hypothesis that higher surface-exposed positive charge correlates with increased delivery efficiency^2,15^. This is further corroborated by studies showing that the net charge of positively supercharged GFP variants strongly influences cellular uptake^16^. However, due to the limited number of surface-exposed amino acid residues in both CPPs and currently available supercharged proteins or polypeptides, achieving higher positive charge density remains challenging. This limitation fundamentally restricts their potential for efficient intracellular delivery. Notably, early supercharged protein systems generally operate within moderate net charge ranges (e.g., GFP + 36^16,17^) and exhibit a critical bottleneck: delivery efficiency drops sharply for cargos larger than ~150 kDa, due to steric hindrance during internalization and inevitable sequestration in endolysosomal compartments^18^. To obtain a more impactful, universal, and biocompatible method for intracellular protein delivery, we aim to devise an approach that can overcome the current limitations.

**Fig. 1:**
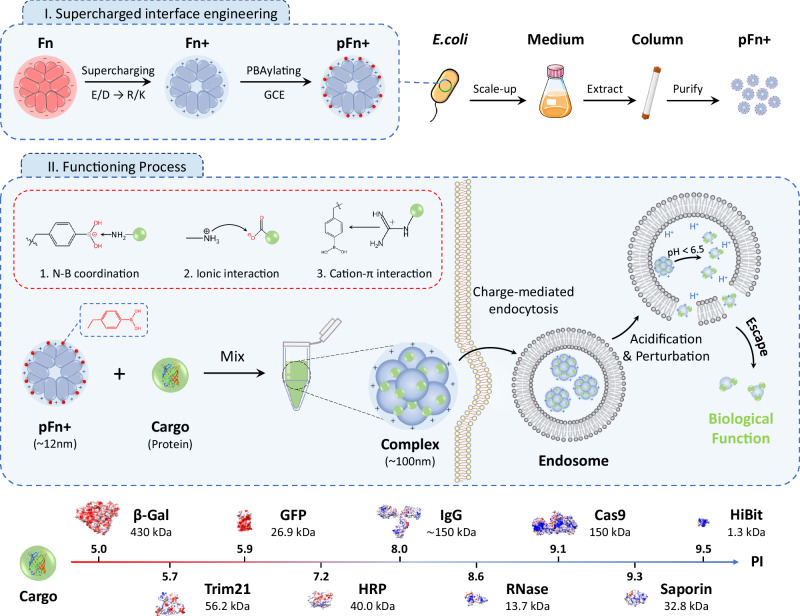
Schematic illustration of the design and functionality of a protein nanocage-based delivery platform. This platform utilizes a ferritin (Fn) nanocage, engineered through a two-step protein engineering strategy to achieve supercharged interfaces. First, site-directed mutagenesis replaces solvent-exposed anionic residues (aspartic acid [D] and glutamic acid [E]) with cationic residues (lysine [K] or arginine [R]) at optimized positions on each ferritin monomer, yielding Fn+. Second, genetic code expansion (GCE) technology incorporates the unnatural amino acid 4-boronophenylalanine (4-BPA) at a defined surface site, appending phenylboronic acid (PBA) moieties, yielding pFn+. This pFn+ nanocage can be efficiently produced at scale in a cost-effective manner using an *E. coli* expression system. The platform enables the straightforward formation of complexes through simple mixing with various protein cargoes. These complexes are subsequently internalized by mammalian cells with high efficiency and released into the cytoplasm, where they exert their biological functions both in vitro and in vivo.

Ubiquitous protein nanocages, such as viral capsids, ferritin, small heat shock proteins, and DNA-binding proteins from starved cells, fulfill a variety of functions, while their shell-like structures hold great promise for various applications in the field of nanomedicine and nanotechnology^19,20^. Here, we propose a supercharged interface engineering strategy to redesign a naturally occurring protein nanocage with different surface positive charges for enhanced delivery efficiency (Fig. 1). Owing to the high surface/volume ratio, a super high surface charge (ranging from −168 to +312) protein nanocage is obtained. The results indicated that the surface charges of the protein nanocages affect the level of cellular uptake. To enable reversible covalent conjugation between the supercharged nanocage and cargo proteins, we genetically incorporated a phenylboronic acid (PBA)^21,22^-bearing unnatural amino acid (UAA) at solvent-exposed sites of the nanocage. We further attempted to investigate the process of protein binding, cellular uptake, endosomal escape, and protein release. Importantly, our study demonstrates that the bioengineered nanocage can successfully deliver various cargo proteins into cells, especially including Cas9/gRNA complex, antibodies, as well as the Trim21/antibody complex. These findings provide mechanistic insights into the unusual ability of bioengineered nanocages to enter cells and deliver cargo proteins, and inform the future development and application of the interface engineering strategy. Overall, these bioengineered nanocages can be used as general cytosolic delivery vehicles for a broad range of functional proteins.

## Results

### Rational design of supercharged protein nanocage

**Fig. 2:**
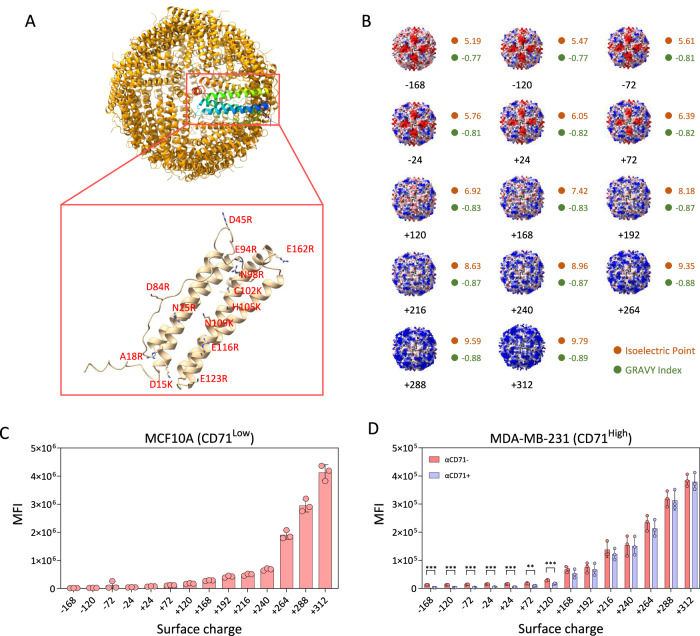
Rational design and screening of Fn+. **A** Thirteen potential supercharged mutation sites on the surface of Fn monomers were predicted and marked in red. **B** The predicted surface electrostatic potential distribution, theoretical isoelectric point, and GRAVY parameter of supercharged ferritins with different net charges (absolute values), observed along a fourfold symmetry axis. Red: −5 kTe^−1^, blue: +5 kTe^−1^. **C** Cellular uptake efficiency of Fn+. FITC-labeled proteins at 1 μM were incubated with MCF10A cells at 37 °C for 12 h. The mean fluorescence intensity (MFI) was analyzed using flow cytometry (*n* = 3 independent samples). **D** Cellular uptake efficiency of Fn+. FITC-labeled proteins at 1 μM were incubated with MDA-MB-231 cells at 37 °C for 12 h. The CD71 receptor was blocked by using an αCD71 antibody, and the MFI was analyzed using flow cytometry (*n* = 3 independent samples). Data are presented as mean ± SEM; statistical significance was determined by two-way ANOVA with Sidák’s multiple comparisons test (**P < 0.01, ***P < 0.001).

We selected ferritin (Fn) as our model protein nanocage due to its remarkable biocompatibility, surface tailorability and uniform size by self-assembly^23^. As a typical protein nanocage, Fn can spontaneously form a 24-mer protein nanocage with an outer diameter of 12 nm^24^. The outer surface of the Fn cage provides a vast external surface area, and its functionality can be conveniently modified through amino acid mutations, exhibiting strong functional plasticity. To obtain supercharged Fn (Fn+) that was expressible and biologically active, software tools such as Rosetta Supercharge and Alphafold2 were used to comprehensively predict potential mutable sites on the surface of Fn. Initially, the PDB structure of Fn was uploaded to the Rosetta Supercharge module^25^ for the prediction of surface mutable sites. As the fixbb protocol^26^ ran more iterations and the energy assignments of positively charged amino acids increased, the number of mutable sites also increased. Eventually, 13 potential mutable sites on the surface of Fn were identified (Supplementary Fig. 1A). To determine the arginine and lysine residue choices for each hypercharge mutation site, Alphafold2 was used as a structural generation tool for single-site R/K mutations, and mutation sites were determined by synthesizing RMSD scores. The protein structure after single-site mutation predicted by Alphafold2 was shown in Supplementary Fig. 1B. Further, the RMSD scores of the mutated structure was calculated using the Matchmaker algorithm, selecting mutations with smaller RMSD to minimize structural changes (Supplementary Fig. 1C). The final determined mutation sites were D15K, D45R, D84K, E94R, E116R, D123K, E162R, A18R, N25R, N98R, C102K, H105K, and N109K (Fig. 2A). Predictions indicated that as the surface electrostatic potential of Fn+ increased, its theoretical isoelectric point also increased, while the GRAVY index decreased with the increase of polar amino acids (Fig. 2B and Supplementary Fig. 2A). Subsequently, a series of mutant Fn+ with different numbers of surface positive charges from Fn − 168 to Fn + 312 (net charges from −168 to +312) were expressed in *E. coli* (Supplementary Fig. 2B, C). SEC-HPLC showed that the apparent molecular size of Fn+ was comparable to that of wildtype Fn (Supplementary Fig. 3). Dynamic light scattering (DLS) results showed that Fn+ could form spherical protein particles of about 12 nm (Supplementary Fig. 4).

Since wildtype Fn can target CD71 to enter cells^27^, we further investigated the cytosolic accumulation of Fn+ using MCF10A (CD71^Low^) and MDA-MB-231 (CD71^High^) cells (Supplementary Fig. 5A). FITC conjugation did not compromise nanocage integrity, as labeled Fn+ maintained a monodisperse size distribution identical to unlabeled controls under physiological conditions (Supplementary Fig. 5B). In CD71^Low^ cells, there is a correlation between increased surface charge and enhanced cellular entry efficiency of Fn+ (Fig. 2C and Supplementary Fig. 5C). To further investigate the role of CD71 in the cellular uptake of Fn+, we inhibited the CD71 with an αCD71 antibody in CD71^High^ cells. At lower charge ranges (−168 to +120), the efficiency of Fn+ uptake was significantly influenced by the αCD71 blockade; however, at higher charge ranges (+168 to +312), the efficiency of Fn+ uptake was unaffected by αCD71 inhibition (Fig. 2D and Supplementary Fig. 5D). This indicated that when Fn+ possesses a higher surface positive charge, charge-mediated endocytosis predominates over CD71-mediated endocytosis. Overall, these findings demonstrated that increasing the surface charge of the protein nanocage significantly enhances its cellular entry efficiency.

### Screening of the nanocage/cargo protein complex

To utilize Fn+ for cytosolic delivery of cargo proteins, avoiding time-consuming and laborious tasks, such as chemical modifications or genetic engineering, we envisioned introducing a functional group into Fn+ that can interact with various proteins, so that the delivery of different proteins can be achieved conveniently and quickly by forming nanocage/cargo protein complex. Phenylboronic acid (PBA), as a non-covalent protein crosslinking moiety, has gradually gained attention in recent years^22,28^. However, the means of modifying recombinant proteins with PBA remain relatively limited. Using genetic codon expansion (GCE) technology, the MjTyrRS was employed as an orthogonal tRNA/aminoacyl-tRNA synthetase (aaRS) pair to introduce 4-boronophenylalanine (4-BPA) into the recombinant protein^29^, thus facilitating the incorporation of one PBA onto each Fn monomer (Supplementary Fig. 6). The expression of PBA-modified Fn+ (pFn+) in *E. coli* could be observed by adding 4-boronophenylalanine (Supplementary Fig. 7A), the purified pFn+ was verified through Western blot (Supplementary Fig. 7B), DLS and TEM (Supplementary Fig. 7C). Additionally, the trypsin-digested products of pFn+ separated by RP-HPLC showed an increased molecular weight in the peptide segments, indicating the potential incorporation of PBA (Supplementary Fig. 7D, E). A series of b and y ions definitively confirmed that PBA was included at position 7 of pFn+ (Supplementary Fig. 7F). These results indicate that the pMjTyrRS/tRNA synthetase pair can actively introduce 4-BPA into Fn+ to obtain pFn+.

**Fig. 3:**
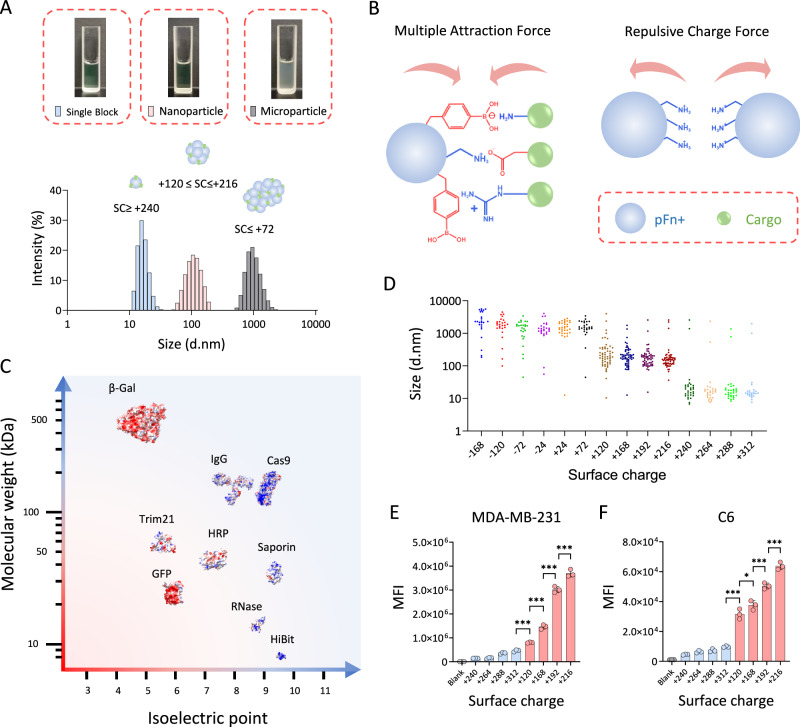
Screening of pFn+/cargo complex. **A** Representative particle size distribution of single block, nanoparticles, and microparticles formed by pFn + @GFP. The abbreviation SC refers to surface charge. **B** Schematic illustration of the principle of complex formation by pFn+ and cargo. **C** Molecular weight and isoelectric point distributions of different proteins, including HiBit, RNase, saporin, GFP, Trim21, HRP, β-gal, Cas9, and IgG. **D** Particle size distributions of complex formed by supercharged ferritin with different net charges (absolute values) with various proteins and peptides (*n* = 498 independent measurements). Flow cytometric analysis of the uptake efficiency from pFn + @GFP (+120 to +312) in MDA-MB-231 (**E**) and C6 (**F**) cells (*n* = 3 independent samples). Data are presented as mean ± SEM; statistical significance was determined by one-way ANOVA with Tukey’s multiple comparisons test (*P < 0.05, ***P < 0.001).

Next, we tried to mix pFn+ with GFP to obtain the pFn+/GFP complex. The results showed a series of unexpected phenomena. First, when the surface positive charge of pFn+ was low (less than +120), a propensity to form larger aggregates or microparticle precipitates exceeding 1000 nm was observed. Second, pFn+ variants from pFn + 120 to pFn + 216 formed nanoparticles approximately 100 nm in size with cage proteins. Third, with a surface positive charge exceeding +240, the particle size distribution narrowed to around 10 nm (Fig. 3A). We speculate that two synergistic factors may override the anticipated electrostatic repulsion in highly negatively charged Fn variants: (1) despite a net negative charge, the surface retains numerous lysine and arginine residues that form localized cationic patches, which can mediate electrostatic adhesion. In contrast, our strategy for introducing positive charge mutations involves replacing pre-existing acidic residues, progressively eliminating negative charges and yielding a surface dominated by uniformly distributed positive charges. As a result, increasing the number of positive mutations enhances electrostatic repulsion. (2) The PBA moieties exhibit strong binding affinity toward cargo proteins; however, the mixed-charge surface of negatively charged Fn—with coexisting positive and negative patches—fails to generate sufficient repulsive force to counteract PBA-mediated attraction, ultimately resulting in the formation of large aggregates (~1000 nm). Conversely, strongly cationic variants (≥ + 240) possess a homogeneous surface charge landscape, where electrostatic repulsion dominates and effectively suppresses PBA-driven aggregation, yielding small assemblies (~10 nm). At intermediate charge states (+120 to +216), the balance between attractive and repulsive forces permits formation of ~100 nm nanocomplexes (Fig. 3B). This inverse relationship between charge magnitude and particle size was confirmed by an additional 498 evaluations of particle sizes formed by mixing pFn+ with different proteins, including GFP, HRP, β-gal, Saporin, RNase, Cas9, and IgG (Fig. 3C, D). Therefore, we selected pFn+ (+120 to +312), which is less prone to precipitation with cargo protein, for further study. We assessed the cellular uptake efficiency of pFn+ (+120 to +312) using GFP as the cargo. The fluorescence properties of pFn + @GFP (+120 to +312) closely resembled those of GFP alone, suggesting that pFn+ does not impair GFP functionality (Supplementary Fig. 8A). Flow cytometry analysis revealed that the uptake efficiency of pFn + @GFP (+120 to +216) was significantly greater than that of pFn + @GFP (+240 to +312), with pFn+216@GFP demonstrating the highest efficiency in MDA-MB-231 and C6 cells (Fig. 3E, F, and Supplementary Fig. 8B, C). These findings suggested that larger nanoparticles (~100 nm) exhibit greater cellular uptake efficiency compared to smaller nanoparticles (~10 nm), likely due to enhanced surface charge density that promotes membrane interaction. Specifically, quantitative analysis of GFP fluorescence intensity in MDA-MB-231 and C6 cells showed that pFn+216@GFP achieved a markedly higher signal compared to GFP + 36 (Supplementary Fig. 8D, E). Furthermore, to determine the role of PBA modification, we compared GFP delivery mediated by unmodified Fn + 216 versus pFn + 216. PBA-modified Fn + 216 formed stable, monodisperse complexes, with an average hydrodynamic diameter of approximately 100 nm (Supplementary Fig. 9A). Moreover, only pFn + 216 successfully facilitated intracellular GFP delivery (Supplementary Fig. 9B–D), indicating that PBA modification is essential for enabling Fn + 216 to functionally interact with cargo proteins. Consequently, pFn + 216 was selected as the most efficient delivery vector for subsequent studies.

### Study on the cellular entry of cage/cargo protein complex

**Fig. 4:**
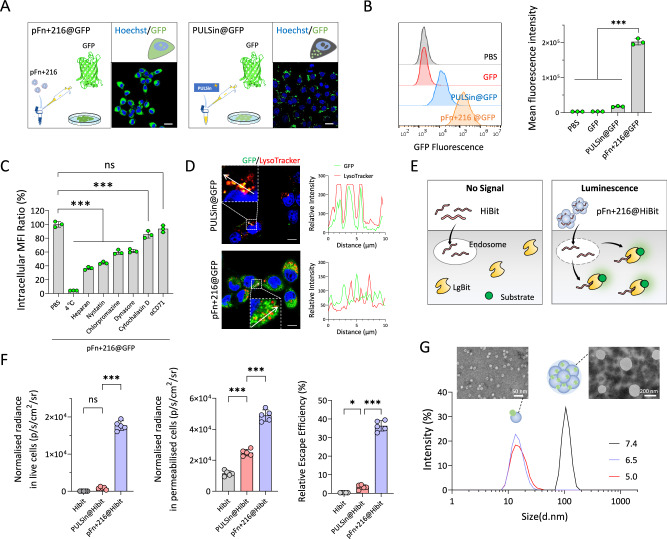
Cellular uptake kinetics of the pFn+216@GFP complex. **A** MDA-MB-231 cells imaged by laser scanning confocal microscopy (LSCM) after incubation with pFn+216@GFP (molar ratio of 1:8 for pFn+: GFP, 1 μM GFP) for 1 h. Cell nuclei were stained with Hoechst 33342 (blue). Scale bar, 20 μm. **B** Flow cytometry analysis of pFn + 216-mediated GFP delivery in live cells (*n* = 3 independent samples). Data are presented as mean ± SEM; statistical significance was determined by one-way ANOVA with Dunnett’s multiple comparisons test (***P < 0.001). **C** Flow cytometry analysis of the cellular uptake mechanism of pFn + 216 (*n* = 3 independent samples). The MFI of pFn+216@GFP was set as 100% as a positive control. Data are presented as mean ± SEM; statistical significance was determined by one-way ANOVA with Dunnett’s multiple comparisons test (ns, not significant; ***P < 0.001). **D** Confocal imaging revealed that in MDA-MB-231 cells treated with pFn+216@GFP for 1 h, the delivered GFP colocalized with LysoTracker, and cell nuclei with Hoechst 33342 (blue). Scale bar, 10 μm. **E**, **F** Comparison of lysosomal escape efficiency between pFn+216@HiBit and PULSin@HiBit using the HiBiT-LgBiT assay (*n* = 5 independent samples). Data are presented as mean ± SEM; statistical significance was determined using one‑way ANOVA with Tukey’s multiple comparisons test (ns, not significant; *P < 0.05, ***P < 0.001). **G** Particle size of pFn+216@GFP at different pH levels was detected by DLS.

Next, the cytosolic protein delivery capability of pFn + 216 was validated. We investigated the appropriate molar ratio between pFn + 216 and GFP, and based on flow cytometry results, we selected a 1:8 molar ratio as the optimal condition for further study (Supplementary Fig. 10A). The cellular uptake efficiency of pFn+216@GFP exhibited a concentration and time-dependent pattern (Supplementary Fig. 10B, C). Additionally, confocal microscopy video capture revealed rapid GFP uptake, observable as early as 60 s (Supplementary Fig. 10D and Supplementary Movie 1). Further examination was conducted on the influence of serum on the uptake efficiency. Results demonstrated that, although there was approximately a 65% decrease in the cellular uptake of GFP in 100% serum, the average fluorescence intensity was still over 30 times greater than that of the control group (Supplementary Fig. 10E). Comparative analysis revealed that pFn+216@GFP exhibited markedly enhanced cellular uptake and intracellular localization compared to the conventional protein transfection reagent PULSin@GFP (Fig. 4A). Flow cytometry further confirmed that pFn + 216 efficiently mediates intracellular GFP delivery while maintaining high cell viability (Fig. 4B and Supplementary Fig. 10F).

Furthermore, the stability of the pFn+216@GFP nanocomplexes across different production batches was validated. DLS analysis revealed nearly overlapping particle size distribution curves for three batches, with peak diameters consistently around 100 nm (Supplementary Fig. 11A), indicating batch-independent monodispersity of the complexes. Flow cytometry further confirmed functional consistency: GFP fluorescence intensity upon cellular uptake was similar across batches, with no significant differences in mean fluorescence intensity (Supplementary Fig. 11B). Notably, this efficient delivery mediated by pFn + 216 was remarkably consistent across multiple diverse cell lines (MDA-MB-231, C6, A549, PC12, BEL-7402, and Jurkat cells) and notably effective in transducing hard-to-transfect primary cells, such as T lymphocytes and hepatocytes, highlighting its utility as a delivery vehicle (Supplementary Fig. 12A). In direct comparison with a panel of established CPPs showed that pFn + 216 significantly outperformed all tested candidates in intracellular GFP delivery efficiency (Supplementary Figs. 13, 14, and Supplementary Table 1).

To elucidate the internalization mechanism of pFn+216@GFP, MDA-MB-231 cells were pre-treated under various conditions before co-incubation with pFn+216@GFP. Flow cytometry analysis revealed that at 4 °C, GFP internalization was completely inhibited, suggesting that the uptake of the complex is an active, energy-dependent process characteristic of endocytosis. Moreover, both heparan and nystatin significantly reduced cellular uptake efficiency by approximately 60%, implying that the internalization of the complex is predominantly reliant on heparan sulfate proteoglycans and caveolin-mediated pathways. Conversely, chlorpromazine and dynasore, known inhibitors of clathrin-mediated endocytosis, exerted a moderate effect on the uptake of the complex. In contrast, αCD71 and cytochalasin D appeared to have negligible influence on the internalization process (Fig. 4C and Supplementary Fig. 12B). Collectively, these findings suggested that the cellular entry of pFn+216@GFP involves multiple endocytic mechanisms. Now that one of the major obstacles in intracellular delivery is the entrapment of biomacromolecules in the endosome, we next performed a colocalization analysis of GFP and lysosome. The colocalization of the delivered GFP with lysotracker (lysosome marker) was studied. Colocalization analysis indicated that at 1 h, GFP was diffusely distributed throughout the cytoplasm (Fig. 4D). In parallel, we employed the HiBiT-LgBit system to quantitatively evaluate lysosomal escape efficiency^30,31^. This method specifically measures cytoplasmic HiBiT peptide fluorescence relative to total cellular fluorescence (Fig. 4E). The results indicate that the lysosomal escape efficiency of pFn+216@HiBit (36.3%) is significantly higher than that of PULSin@HiBit (3.4%). Meanwhile, compared to PULSin, the total intracellular uptake and cytoplasmic quantity were approximately 2.0-fold and 20.7-fold higher, respectively (Fig. 4F). Chloroquine treatment significantly enhanced pFn + 216-mediated delivery, further indicating endosomal involvement (Supplementary Fig. 12C). Collectively, these results indicate that pFn + 216 facilitates efficient escape of its cargo from early endosomes prior to lysosomal degradation, leading to significant accumulation within the cytoplasm. To explore the disassembly process of the complex within cells, we measured its particle size at various pH levels. The results indicated that the size of the complex was approximately 100 nm at pH 7.4, but decreased to approximately 10 nm when the pH was below 6.5, suggesting that dissolution occurs due to the disruption of intermolecular interactions (Fig. 4G). In addition, control experiments confirm that only pFn + 216 exhibit no significant size change across pH 5.0–7.4, demonstrating that the pH-responsive behavior is exclusive to the pFn+216@cargo protein complexes (Supplementary Fig. 11C). The complex disassembles under acidic conditions, such as those found in endosomes, thereby reducing spatial shielding and restoring the biological activity of active proteins.

We further conducted multidimensional safety evaluations of the system across both in vitro and in vivo models. For cytocompatibility, pFn + 216 exhibited no significant cytotoxicity (Supplementary Fig. 15A–F). Hemocompatibility assessments showed no significant hemolysis at concentrations up to 10 µM (Supplementary Fig. 15G). The in vivo safety assessment encompassed acute toxicity screening (14-day observation), multi-organ histopathology (H&E staining of liver, spleen, kidney, and heart; Supplementary Fig. 16A), serum biochemistry (ALT/AST for liver function, BUN/creatinine for kidney function; Supplementary Fig. 16B, C), and cytokine profiling (Supplementary Fig. 16D)—provides comprehensive evidence supporting the biosafety of pFn + 216 at therapeutically relevant doses. To further evaluate potential cellular stress responses induced by pFn + 216-mediated protein delivery, we conducted comprehensive transcriptomic profiling. RNA-seq analysis comparing pFn + 216-treated and vehicle control cells identified 118 significantly differentially expressed genes (DEGs), comprising 38 upregulated and 80 downregulated transcripts (Supplementary Fig. 17A). Crucially, gene set enrichment analysis (GSEA) showed no enrichment in canonical stress-response or cell death pathways (e.g., p53 signaling, apoptosis, ER stress). Gene Ontology (GO) analysis indicated the DEGs were primarily associated with physiological regulatory processes, including voltage-gated ion channel activity, cell-cell junction assembly, and basolateral plasma membrane organization, consistent with homeostatic cellular adaptation (Supplementary Fig. 17B). KEGG analysis further confirmed enrichment solely in benign functional pathways, most notably taste transduction (Supplementary Fig. 17C). Collectively, these preliminary findings support the potential of pFn + 216 as an effective and safe protein delivery carrier.

### Intracellular delivery of bioactive proteins

**Fig. 5:**
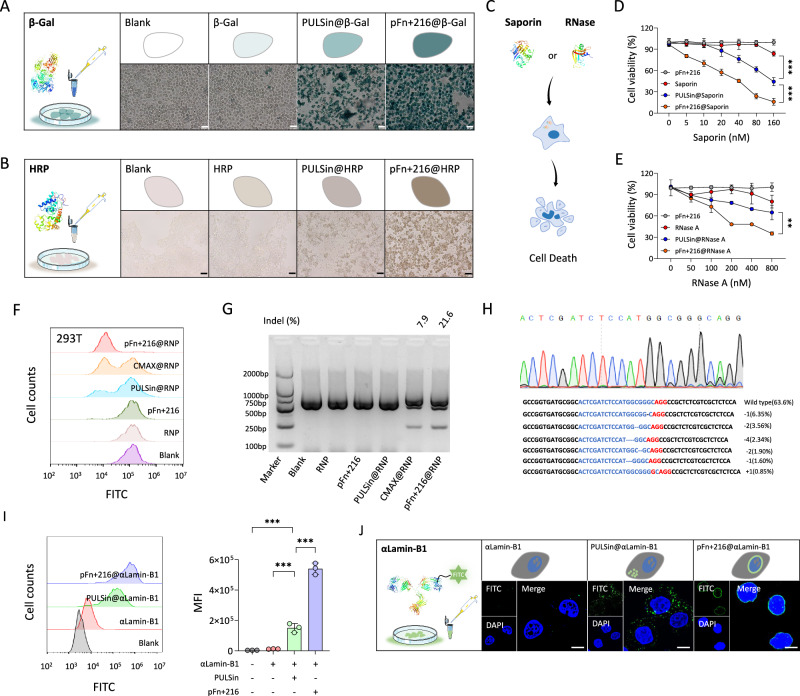
Intracellular delivery of bioactive proteins. **A** Staining image of MDA-MB-231 cells after 24 h of treatment with β-gal, PULSin@β-gal, and pFn+216@β-gal. Untreated MDA-MB-231 cells served as a negative control. β-gal catalyzes the conversion of X-gal into a blue precipitate. Scale bar: 50 μm. **B** Intracellular delivery of HRP mediated by pFn + 216. MDA-MB-231 cells were incubated with pFn+216@HRP for 24 h, followed by 3,3’-diaminobenzidine (DAB) staining. Untreated MDA-MB-231 cells were used as a negative control. HRP catalyzes the oxidation of DAB to produce a brown precipitate. Scale bar: 50 μm. **C**Schematic illustration of cytoplasmic delivery of toxic proteins (Saporin or RNase A) mediated by pFn + 216. **D**, **E** Cytotoxicity of pFn+216@Saporin and pFn+216@RNase A. MDA-MB-231 cells were treated with either pFn+216@Saporin (**D**) or pFn+216@RNase A (**E**) for 24 h; cell viability was assessed using the MTT assay (*n* = 3 independent samples). Data are presented as mean ± SEM; statistical significance was determined by two-way ANOVA with Sidak’s multiple comparisons test (**P < 0.01, ***P < 0.001). **F** Flow cytometry analysis and quantification of average fluorescence intensity in HEK-293T-GFP cells treated with free RNP, pFn + 216, PULSin@RNP, CMAX@RNP, or pFn+216@RNP. **G** Detection of GFP gene cleavage in HEK-293T-GFP cells treated with pFn+216@RNP using the T7 endonuclease I (T7E1) assay. **H** DNA sequencing results demonstrating GFP gene editing in HEK-293T-GFP cells treated with pFn+216@RNP. **I** Flow cytometric analysis of the change in average fluorescence intensity of MDA-MB-231 cells incubated with pFn+216@αLamin-B1 for 24 h (*n* = 3 independent samples). Data are presented as mean ± SEM; statistical significance was determined using one‑way ANOVA with Tukey’s multiple comparisons test (***P < 0.001). **J** Confocal microscopy image of MDA-MB-231 cells incubated with pFn+216@αLamin-B1. After 24 h of incubation, cells were fixed, permeabilized, and stained with DAPI (blue) to visualize nuclei. Scale bar: 10  μm.

In addition to potent cytoplasmic delivery, the bioactivity of proteins must be preserved after pFn + 216 delivery. β-galactosidase (β-Gal; Mw = 430 kDa, pI = 5.0) and horseradish peroxidase (HRP; Mw = 40.0 kDa, pI = 7.2) were chosen as model proteins (Supplementary Fig. 18A, B). MDA-MB-231 cells treated with pFn+216@β-Gal were then incubated with X-gal (a substrate of β-Gal that produces a blue product), and the cells treated with pFn+216@β-Gal displayed a more pronounced blue color compared to those treated with PULSin@β-Gal (Fig. 5A). The activity of HRP was detected using DAB (which reacts with HRP to produce a brown product) staining (Fig. 5B). MDA-MB-231 cells treated with pFn+216@HRP exhibited significantly higher intracellular HRP activity compared to the control group.

We further investigated the delivery efficacy of pFn + 216 for transporting toxic proteins (Fig. 5C). The analysis revealed that the complex formed by pFn + 216 with Saporin (Mw = 32.8 kDa, pI = 9.4) or RNase (Mw = 13.7 kDa, pI = 9.5) exhibited consistent size and morphology (Supplementary Fig. 18C, D). The cytotoxicity exhibited by the pFn+216@RNase was significantly greater than that of the PULSin@RNase, with RNase alone exerting minimal effect on cellular proliferation (Fig. 5D). After 24 h of treatment with the pFn+216@Saporin, the cell proliferation of MDA-MB-231 cells was notably suppressed (Fig. 5E).

Further investigations were conducted to evaluate the gene editing efficiency mediated by the delivery of Cas9/sgRNA ribonucleoprotein (RNP) complexes via pFn + 216. Characterization results demonstrated that pFn + 216 formed stable complexes with RNP, exhibiting uniform size distribution and morphology (Supplementary Fig. 18E). Confocal microscopy analysis revealed that FITC-labeled RNP complex delivered by pFn + 216 efficiently localized to the cell nucleus (Supplementary Fig. 19), indicating successful lysosomal escape and nuclear targeting facilitated by the nuclear localization sequence (NLS). To assess functional gene editing, we assembled RNP complexes consisting of sgRNA targeting the GFP gene and Cas9-NLS, which were subsequently delivered into HEK-293T-GFP cells using pFn + 216. As shown in Fig. 5F, a significant reduction in GFP fluorescence intensity was observed in the pFn+216@RNP-treated cells compared to controls. Consistent with this, T7E1 cleavage assays confirmed a marked decrease in GFP protein expression levels (Fig. 5G). Furthermore, Sanger sequencing analysis validated the gene editing efficiency of pFn+216@RNP at the target GFP locus (Fig. 5H).

Delivering functional antibodies within living cells would enable the labeling and manipulation of intracellular antigens, a long-standing goal in cell biology and medicine^32^. Given the above, we believe that pFn + 216 can be easily applied to cytosolic antibody delivery. The efficacy of pFn + 216 in antibody delivery was evaluated using nonspecific IgG (derived from normal rabbit serum; Mw ≈ 150 kDa, pI  ≈ 8.0). The results demonstrated a clear concentration-dependent delivery of pFn+216@IgG with uniform size and morphology (Supplementary Figs. 18F and 20). ΑLamin-B1 (αLamin-B1), chosen as the model antibody due to its utility in analyzing nuclear envelope morphology, was employed to assess the antigen-binding activity of the delivered antibody in live cells. A complex comprising pFn+216@αLamin-B1 was incubated with MDA-MB-231 cells. Flow cytometry analysis confirmed that pFn+216@αLamin-B1 achieved significant uptake efficiency (Fig. 5I). Furthermore, confocal microscopy revealed distinct circular fluorescent signals around the outer nuclear membrane in live cells (Fig. 5J). Overall, these results collectively demonstrated that pFn + 216 can mediate highly efficient intracellular delivery of bioactive proteins.

### pFn+216@RNP complex effectively edits organs in vivo

**Fig. 6:**
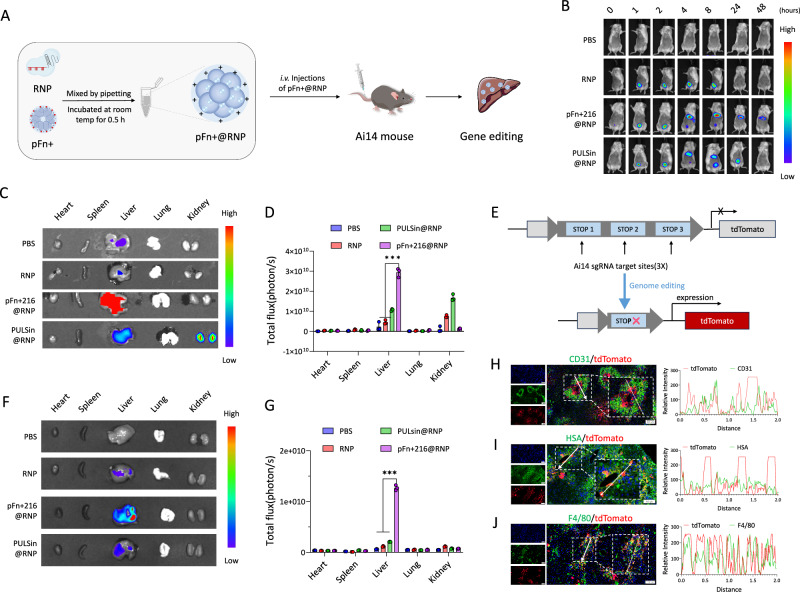
Applications for in vivo gene editing. **A** Schematic illustration of the experimental design for pFn+216@RNP-mediated gene editing in Ai14 tdTomato mice. **B** In vivo fluorescence imaging of mice at 0, 1, 2, 4, 8, and 24 h post-intravenous injection of PBS (control), RNP, pFn+216@RNP, and PULSin@RNP (6 mg Cy7-labeled Cas9 equivalent/kg). **C**Ex vivo fluorescence imaging of major organs harvested 24 h after intravenous injection of pFn+216@RNP and the control (6 mg Cy7-labeled Cas9 equivalent/kg). **D** Quantitative analysis of Cas9-Cy7 accumulation in various organs (*n* = 3 independent animals). Data are presented as mean ± SEM; statistical significance was determined by two-way ANOVA with Sidák’s multiple comparisons test (***P < 0.001). **E** Schematic representation of the Ai14 mouse model, which harbors a STOP cassette flanked by three SV40 polyA sequences to suppress downstream tdTomato expression. Successful gene editing mediated by Cas9 RNP targeting the SV40 polyA sequence results in the excision of the STOP cassette, enabling tdTomato expression. **F** In vivo imaging revealed that intravenous injection of pFn+216@RNP selectively induced tdTomato expression in the liver. **G** Quantification of total tdTomato fluorescence flux in major organs demonstrated a significant increase exclusively in the liver, with no detectable enhancement in other organs (*n* = 3 independent animals). Data are presented as mean ± SEM; statistical significance was determined by two-way ANOVA with Sidák’s multiple comparisons test (***P < 0.001). **H–J** Immunofluorescence staining of liver sections from mice injected with pFn+216@RNP confirmed extensive gene editing. Liver sections were stained with αRFP antibody for tdTomato (red), an αCD31 antibody for endothelial cells (green in **H**), an αHSA (hepatocyte-specific antigen) antibody for hepatocyte (green in **I**), an αF4/80 antibody for Kupffer cells (green in **J**), and DAPI for nucleus (blue). Representative images are shown. Scale bar: 100 μm.

Having demonstrated the efficient delivery of RNP by pFn + 216 in vitro, we next evaluated the potential of this delivery strategy to mediate genome editing in vivo following intravenous administration in mice. Initially, the biodistribution of pFn+216@RNP (Cy7-labeled Cas9) was assessed (Fig. 6A). After a single intravenous injection, Cy7 fluorescence was monitored at predetermined time intervals from 0 to 48 h. Surprisingly, the pFn+216@RNP group showed significantly higher fluorescence intensity in the liver compared to both the naked RNP and PULSin@RNP groups (Fig. 6B). Ex vivo imaging of major organs at 24 h post-injection confirmed strong hepatic accumulation of fluorescence specifically in the pFn+216@RNP group (Fig. 6C, D), aligning with the in vivo data. To precisely identify the cellular tropism within the liver, we performed flow cytometric analysis of primary hepatocytes, Kupffer cells, and endothelial cells isolated from treated mice. This analysis revealed that Kupffer cells exhibited the highest uptake efficiency of pFn+216@RNP, with 41.0% of F4/80-positive cells showing high Cy7 signal, significantly surpassing the uptake by hepatocytes (8.98%) and endothelial cells (28.3%) (Supplementary Fig. 21). This biodistribution profile indicates that pFn+216@RNP achieves unexpectedly liver-targeted delivery.

To further investigate the in vivo gene editing efficiency of intravenously administered pFn+216@RNP, Ai14 mice were utilized. The genome of Ai14 mice harbors a LoxP-flanked stop cassette that inhibits downstream expression of tdTomato (Fig. 6E). This stop cassette, comprising three SV40 polyA transcription terminators, serves as a target for RNP-mediated editing. Ai14 mice were intravenously injected with pFn+216@RNP on day 0, and major organs were harvested on day 7. The pFn+216@RNP successfully targeted the SV40 polyA sequence, resulting in a marked increase in tdTomato fluorescence in the liver, indicative of successful gene editing (Fig. 6F, G). Immunofluorescence revealed tdTomato expression in multiple hepatic cell types—hepatocytes, endothelial cells, and Kupffer cells (Fig. 6H–J). Quantitative colocalization analysis showed predominant accumulation in Kupffer cells, with strong overlap between F4/80 and tdTomato signals (Fig. 6J). This Kupffer cell-preferential uptake suggests that pFn + 216 is particularly suitable for targeting macrophage-associated liver disorders. Together, these results demonstrate that pFn + 216 mediates efficient RNP delivery and functional gene editing in vivo, supporting its potential as a versatile platform for liver-directed therapies.

### Antibody-mediated degradation of endogenous proteins

**Fig. 7:**
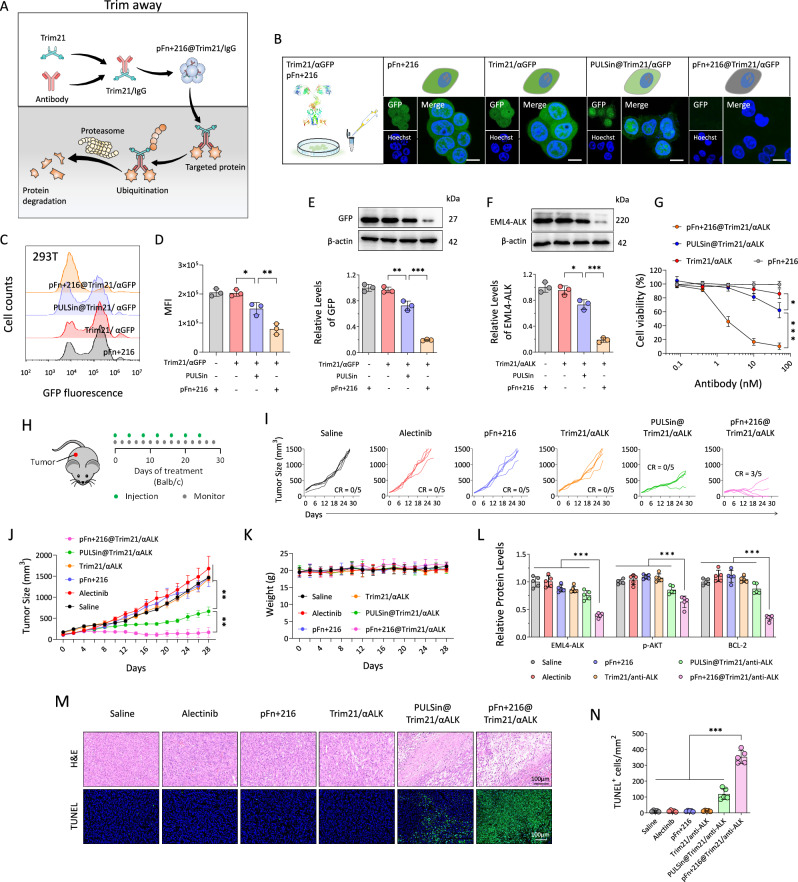
Applications for intracellular antibody delivery. **A** Schematic illustration of the trim-away mechanism. **B** Confocal microscopy image of a monolayer of HEK-293T-GFP cells incubated with pFn+216@Trim21/αGFP for 24 h. Cell nuclei were stained with Hoechst 33342 (blue). Scale bar: 10 μm. **C**, **D** Flow cytometric analysis of the change in average fluorescence intensity of HEK-293T-GFP cells treated with pFn+216@Trim21/αGFP (*n* = 3 independent samples). Data are presented as mean ± SEM; statistical significance was determined using one‑way ANOVA with Tukey’s multiple comparisons test (*P < 0.05, **P < 0.01). **E** Immunoblot analysis demonstrating targeted degradation of GFP protein in HEK-293T-GFP cells mediated by pFn+216@Trim21/αGFP (*n* = 3 independent samples). Data are presented as mean ± SEM. **F** Immunoblot analysis showing targeted degradation of EML4-ALK protein in cells mediated by pFn+216@Trim21/αALK (*n* = 3 independent samples). Data are presented as mean ± SEM; statistical significance was determined using one‑way ANOVA with Tukey’s multiple comparisons test (*P < 0.05, **P < 0.01, and ***P < 0.001). **G** Cytotoxicity of pFn+216@Trim21/αALK was assessed using an MTT assay (*n* = 3 independent samples). Data are presented as mean ± SEM; statistical significance was determined by two-way ANOVA with Sidák’s multiple comparisons test (*P < 0.05, ***P < 0.001). **H** Schematic diagram illustrating the antitumor effect in an H3122/Ale subcutaneous tumor model. Mice were randomly assigned to six groups (saline, alectinib, pFn + 216, Trim21/αALK, PULSin@Trim21/αALK, and pFn+216@Trim21/αALK). Mice were administered intratumoral injections of saline, pFn + 216, Trim21/αALK, PULSin@Trim21/αALK, or pFn+216@Trim21/αALK complex per mouse (equivalent to 1 μg αALK IgG) every 4 days. The Alectinib group received oral administration (20 mg/kg/day). **I** Tumor growth curves of different treatment groups every 2 days. CR complete response, defined as tumor volume less than 50 mm^3^ for the remainder of the experiment. **J** Average tumor growth curves were recorded over 21 days (*n* = 5 independent animals). Data are presented as mean ± SEM; statistical significance was determined by two-way ANOVA with Sidák’s multiple comparisons test (**P < 0.01). **K** Average body weight after different treatments were recorded every 2 days (*n* = 5 independent animals). Data are presented as mean ± SEM. **L** Western blot analysis of p-AKT, total AKT, BCL-2, and EML4-ALK under different treatments, with protein expression levels determined by densitometry (**L**) (*n* = 5 independent animals). Data are presented as mean ± SEM; statistical significance was determined by one-way ANOVA with Dunnett’s multiple comparisons test (***P < 0.001). **M** Representative images of tumor sections stained with H&E and TUNEL assay following different treatments. **N** TUNEL staining of tumor tissue sections. Quantitative analysis was performed with 3 randomly selected fields per slide and 5 animals per group. Data are presented as mean ± SEM; statistical significance was determined by one-way ANOVA with Dunnett’s multiple comparisons test (***P < 0.001).

The recently discovered “trim-away” mechanism opens a window for the rapid and selective degradation of endogenous proteins without prior modification of the genome or mRNA^33^. In the trim-away process, the target protein is initially bound by an off-the-shelf antibody, followed by the intracellular antibody receptor Trim21-mediated ubiquitination that forms a protein complex, which is subsequently degraded in the proteasome. However, the broad application of trim-away is significantly limited by the inability of antibodies to permeate the cell membrane. To address this limitation, Clift et al. introduced antibodies into cells using microinjection and electroporation techniques^34^, which necessitate specialized skills or equipment. Consequently, there is a pressing need to develop a more accessible trim-away approach. In this study, we employed pFn + 216 to deliver a complex comprising αGFP antibody and Trim21 protein to HEK-293T-GFP cells, aiming to evaluate its capability for targeted protein degradation (Fig. 7A). Confocal microscopy and flow cytometry showed that pFn + 216 effectively mediated the degradation of GFP in HEK-293T-GFP cells via the Trim21/αGFP complex (Fig. 7B–D). Additionally, Western blot analysis further revealed that the pFn+216@Trim21/αGFP successfully achieved GFP knockdown (Fig. 7E). Furthermore, we included two additional critical controls: an isotype control antibody (IgG) and a catalytically inactive Trim21 RING-domain mutant (C16A)^35^. Neither control resulted in target protein degradation compared to the pFn+216@Trim21/αGFP group, confirming that both antigen-specific binding and functional Trim21 E3 ligase activity are essential for this process (Supplementary Fig. 22).

EML4-ALK, a well-characterized oncogene, encodes a cytoplasmic fusion protein that drives tumor progression^36^. Resistance to small-molecule ALK inhibitors often leads to treatment failure^37^, highlighting the need for alternative strategies, such as targeted protein degradation. To validate this hypothesis, we established an Alectinib-resistant H3122 subline (H3122/Ale) through chronic, escalating exposure to Alectinib. Compared to the parental cells, H3122/Ale exhibited upregulation of the efflux transporters ABCB1 (P-glycoprotein) and ABCC1 (MRP1), which are ATP-binding cassette (ABC) transporters widely associated with multidrug resistance phenotypes in cancer cells^38–41^. Consistent with these observations, H3122/Ale cells displayed an approximately 16.2-fold increase in Alectinib resistance compared to parental cells (Supplementary Fig. 23A, B), indicating a markedly reduced drug sensitivity. Functional assays confirmed a markedly attenuated response: while parental H3122 cells showed potent, dose-dependent inhibition at low Alectinib concentrations (1–100 nM), significant effects in H3122/Ale cells were only observed at the highest dose (100 nM) (Supplementary Fig. 23C–F). Critically, western blot analysis verified that treatment with pFn+216@Trim21/αALK significantly reduced EML4-ALK protein levels in H3122/Ale cells (Fig. 7F) and markedly suppressed cell proliferation (Fig. 7G). To ascertain whether these effects specifically resulted from the intracellular delivery of functional cargo, we performed Annexin V/PI flow cytometry. Apoptosis was significantly enhanced only in cells treated with the complete pFn+216@Trim21/αALK complex, confirming that the pro-apoptotic effect is cargo-dependent and requires efficient nanocage-mediated delivery (Supplementary Fig. 23G, H). To further demonstrate that the anti-tumor effect depends on proteasomal degradation of the target protein, we employed the proteasome inhibitor MG132. Co-treatment with MG132 effectively reversed the key outcomes induced by pFn+216@Trim21/αALK: it prevented EML4-ALK degradation (Supplementary Fig. 24A), restored cell viability in CCK-8 assays (Supplementary Fig. 24B), and largely rescued clonogenic survival (Supplementary Fig. 24C, D).

To evaluate the in vivo anti-tumor potential of this system, we performed real-time imaging after intratumoral injection of Cy7-labeled complexes. Compared to control groups (IgG-Cy7 alone and PULSin@IgG-Cy7), the pFn+216@IgG-Cy7 group exhibited significantly more uniform penetration and diffusion throughout the tumor tissue (Supplementary Fig. 25), demonstrating the superior distribution capability of our nanocage platform. We further investigated the dose-dependent therapeutic efficacy. Using the H3122/Ale subcutaneous tumor model, intratumoral injections of pFn+216@Trim21/αALK complexes at three distinct doses (low, 0.25 µg; medium, 0.5 µg; and high, 1.0 µg αALK IgG per mouse) revealed a clear dose-dependent inhibition of tumor growth, with the high-dose group showing the most potent effect and no associated systemic toxicity (Supplementary Fig. 26A–D). Further analysis of tumor lysates revealed a dose-dependent downregulation of EML4-ALK, phosphorylated AKT (p-AKT), and the anti-apoptotic protein BCL-2, with the most pronounced reduction observed in the high-dose group (Supplementary Fig. 26E, F). To further assess whether these effects depend on Trim21 catalytic activity, we employed the catalytically inactive Trim21(C16A) mutant^35^. In H3122/Ale cells, while pFn+216@Trim21/αALK effectively reduced EML4-ALK levels, the C16A mutant largely abrogated this effect (Supplementary Fig. 27A), indicating that the degradation process depends on Trim21 catalytic function. The C16A mutant failed to suppress cell viability or inhibit clonogenic survival, whereas wild-type Trim21 retained these effects (Supplementary Fig. 27B, C). Importantly, in vivo validation using H3122/Ale xenografts showed that the C16A mutant lost tumor-suppressive activity, while wild-type Trim21 significantly inhibited tumor growth (Supplementary Fig. 27D–F). These results demonstrate that Trim21 catalytic activity is required for efficient EML4-ALK degradation and is closely associated with the observed antitumor effects. Finally, we evaluated the comparative anti-tumor efficacy of pFn+216@Trim21/αALK (high dose) versus control treatments in H3122/Ale tumor-bearing mice via intratumoral injection (Fig. 7H). Tumor growth inhibition was significantly greater in the pFn+216@Trim21/αALK group compared to the PULSin@Trim21/αALK, Trim21/αALK alone, pFn + 216 alone, Alectinib alone and saline control groups (Fig. 7I, J). Body weights remained stable across all treatment groups, indicating no systemic toxicity (Fig. 7K). Mechanistically, Western blot analysis confirmed the effective depletion of EML4-ALK fusion protein in vivo (Fig. 7L and Supplementary Fig. 27G). Consequently, we observed a marked inactivation of the AKT signaling pathway, reflected by a significant reduction in the p-AKT/AKT ratio (*P* < 0.001). This was accompanied by a significant downregulation of the anti-apoptotic protein Bcl-2 (*P* < 0.001). Histopathological analysis employing H&E staining and TUNEL assays revealed that the pFn+216@Trim21/αALK treatment group exhibited significantly larger areas of necrosis and a higher incidence of apoptosis compared to the other groups (Fig. 7M). Quantitative analysis of the TUNEL assays further confirmed that the co-delivery of pFn+216@Trim21/αALK induced a significantly higher level of apoptosis, markedly outperforming commercial transfection reagents (Fig. 7N). These results demonstrate that pFn + 216 can efficiently deliver the Trim21/antibody complex into cells both in vitro and in vivo. Collectively, this strategy represents a significant advancement in the labeling, delivery, and targeted manipulation of intracellular antigens, offering a powerful tool for both basic research and therapeutic applications.

## Discussion

In this study, we present a supercharged protein nanocage engineering strategy that enables efficient cytosolic delivery of diverse functional proteins. By systematically redesigning the surface charge landscape of Fn nanocages through in silico-guided mutagenesis, we generated a library of Fn+ variants with charges spanning from −168 to +312. Crucially, we demonstrate a charge-dependent cellular internalization mechanism: while low-charge Fn+ relies on CD71-mediated endocytosis, variants with net surface charges exceeding +168 bypass receptor dependency and achieve charge-dominated uptake. This shift from receptor-mediated to charge-driven entry significantly expands the utility of the Fn platform, opening avenues for the delivery of a wider range of therapeutic cargoes beyond natural binding partners.

To achieve universal, non-conjugative cargo protein loading, we pioneered the site-specific incorporation of PBA into pFn+ via genetic code expansion. pFn+ dynamically self-assembles with cargo proteins, but complex formation is exquisitely charge-dependent: moderately charged variants (pFn + 120 to pFn + 216) form stable ~100 nm nanocomplexes, whereas excessive charge (> + 240) disrupts assembly through electrostatic repulsion. This optimal charge density range—peaking at pFn+216— balances nanocomplex stability and cellular uptake efficiency, enabling superior endosomal escape and functional delivery that outperforms commercial reagents like PULSin^TM^. The system demonstrates exceptional safety due to the biocompatible Fn scaffold and reversible cargo protein binding, resulting in negligible cytotoxicity, hemolysis, and organ toxicity. In addition, the system is highly scalable, enabling efficient expression in *E. coli* (unoptimized yield >50 mg/L) without the need for complex chemical modifications.

Notably, in vivo gene editing via systemic administration achieved highly efficient, liver-specific tdTomato activation in *Ai14* mice, leveraging the inherent hepatic first-pass accumulation of nanoparticles. Furthermore, successful antibody-guided intracellular protein degradation (trim-away) was demonstrated for both GFP and the oncogenic driver EML4-ALK; therapeutic pFn + 216 nanoparticles encapsulating Trim21/αALK complexes effectively suppressed tumor growth in vivo, validating intracellular antibody functionality. Beyond these demonstrated applications, the supercharged interface engineering strategy underlying pFn + 216 offers distinct advantages over existing platforms. It exhibits remarkable versatility, efficiently delivering proteins ranging from 13 to 430 kDa, with pI spanning 5.0 to 9.5, including complex multi-component cargos, such as Cas9/sgRNA ribonucleoproteins and Trim21/antibody complexes.

In our in vivo gene editing studies, we observed predominant accumulation of pFn+216@cargo protein complexes in the liver, which presents a substantial advantage for the development of therapeutics targeting liver-associated diseases. Nonetheless, expanding the targeting scope beyond the liver represents a key direction for future optimization. This could be achieved through fusion with tissue-specific homing peptides or small targeting moieties, such as nanobodies or single-chain antibodies. Alternatively, surface charge masking strategies—for example, shielding positive charges with PEG—could enable conditional exposure of the supercharged surface in specific tissue microenvironments to achieve localized delivery. Moreover, the intrinsic cavity of Fn itself offers opportunities for cargo protein loading^42^ (e.g., metal ions or small molecules, such as doxorubicin), enabling the development of multi-cargo co-delivery strategies that fully leverage the native properties of the Fn scaffold. Finally, while our supercharged interface engineering strategy has thus far been applied only to Fn, future studies may extend this approach to other natural or synthetic protein nanocages^43^, providing valuable guidance for the design of next-generation intracellular delivery vehicles with tunable surface or exposed charge properties.

In summary, we engineered a series of innovative supercharged protein nanocages (Fn+) and demonstrated a correlation between increased surface charge and enhanced internalization efficiency. By leveraging genetic codon expansion technology, we incorporated the unnatural amino acid with a PBA group into Fn+ (pFn+), thus achieving efficient and universal cytoplasmic protein delivery without compromising bioactivity. The unique properties of pFn+ are further complemented by low toxicity, stability in mammalian serum, applicability across diverse mammalian cell types, and ease of use by mixing with a protein of interest. Importantly, pFn+ has been shown to effectively deliver Cas9/sgRNA, antibody and Trim21/antibody complex in live cells, thereby facilitating precise intracellular target manipulation. These findings underscore the potential of pFn+ as a versatile, safe, and efficient intracellular protein delivery system, opening an avenue for the enhancement of research tools and the development of therapies targeting intracellular proteins.

## Methods

### Surface mutation site prediction for ferritin nanocage

The Rosetta molecular modeling program was used to identify potential sites in human recombinant ferritin (Fn) for designing a protein nanoparticle with a positive supercharge. Molecular modeling in Rosetta was conducted using the fixbb protocol to fix the position of the amino acid backbone. After energy minimization of the Fn crystal structure (PDB 2CEI) in Rosetta, an appropriate original protein conformation was obtained, followed by surface mutation site screening. The simulation calculated the outer surface of monomeric Fn in the spherical PDB structure of biologically self-assembled Fn, allowing mutations of residues on the surface of the Fn nanocage (54 residues per monomer), but excluding its inner surface or residues in contact with other subunit molecules. Alphafold2 was used to predict the structural deviation of these 9 mutation sites when single mutations to R or K were introduced, comparing the mutated structures with the prototype Fn structure. The root mean square deviation (RMSD) was calculated for the same mutation sites undergoing R or K mutations using the Matchmaker module in ChimeraX against the Fn PDB structure. RMSD is used in protein structure analysis to measure the degree of atomic displacement from alignment positions, and smaller RMSD values after mutation were selected to determine the *R* or *K* values for the mutation sites.

### Construction and purification of Fn nanocage

The gene sequence for wild-type Fn was retrieved from the NCBI. Following the synthesis of the gene sequence of Fn and Fn+ protein by Genscript Inc., the target fragment was subsequently ligated into the pET28a (+) plasmid. Following transformation into BL21(DE3) competent cells, the cells were cultured in LB liquid medium at 37 °C, with agitation at 220 rpm. Genetic Code Expansion (GCE) technology allows termination codons (usually the amber codon UAG) to execute encoding functions, introducing novel amino acids. By transforming the plasmids expressing MjTyrRS aminoacyl-tRNA synthetase and transport RNA into expressing bacterial strains, the nonsense codons (usually the amber codon UAG) can perform encoding, introducing unnatural amino acids at corresponding sites, and endowing proteins with extra biological activities. As shown in Supplementary Fig. 6A, the enzyme introducing 4-boronophenylalanine was co-transformed into the expression system with the plasmids. A final concentration of 10% (v/v) glycerol was added to the freshly cultured bacterial solution, mixed, and the glycerol bacteria were stored at −80 °C for future use.

To prepare pFn+, engineered bacteria containing the expression plasmid were cultured at 37 °C and 220 rpm until the OD_600_ reached 0.6–0.8. Then, IPTG (Aladdin, I104812) was added to a final concentration of 1 mM, and the culture continued for an additional 6 h to induce protein expression. The culture medium was further centrifuged at 4 °C and 8000 × *g* for 15 min to collect the bacterial cells. Bacterial cell pellet was resuspended in buffer (20 mM Tris-HCl, 1 M NaCl, pH 8.0) at a mass-to-volume ratio of 1:20, with the addition of 0.5% (v/v) Triton X-100. The suspension was mixed until no visible clumps remained and subjected to ultrasonic disruption on ice. The resulting lysate was centrifuged at 4 °C and 12,000 × *g* for 15 min to collect the protein pellet. The protein pellet was resuspended in an equal volume of inclusion body wash buffer (20 mM Tris-HCl, 500 mM NaCl, 2 M Urea, pH 8.0) and centrifuged again at 4 °C and 12,000 × *g* for 10 min to wash the sample three times. Then, the washed pellet was then resuspended in a buffer (20 mM Tris-HCl, 500 mM NaCl, 8 M Urea, pH 8.0) and stirred thoroughly for 60 min to ensure complete dissolution. Sample solution was centrifuged at 4 °C and 12,000 × *g* for 20 min, and the supernatant containing the dissolved protein was collected. Protein purification was performed using a Ni^2+^ affinity column (Smart-Lifesciences, China), followed by dialysis into storage buffer (20 mM Tris-HCl, 150 mM NaCl, 20 mM imidazole, pH 7.6). The dialyzed sample was concentrated using a 100 kDa molecular weight cut-off centrifugal ultrafiltration (Beyotime, FUF590). The concentration of the purified protein was measured using a BCA assay kit (Thermo Scientific, 23225), and the protein was stored at −80 °C.

### Study on the cellular uptake mechanism of fluorescent protein delivery complex

The cellular uptake mechanism of pFn+@GFP was investigated by studying the effect of inhibitors of different cellular uptake mechanisms on the efficiency of pFn+@GFP cellular uptake. MDA-MB-231 cells were seeded in a 24-well plate with 30% of the bottom area covered with cells, and the cells were gently shaken and placed in a constant temperature cell incubator at 37 °C and 5% CO_2_ overnight. The next day, the cells were cultured at 4 °C for 30 min, or with heparan sulfate (MedChemExpress, HY-101916), nystatin (MedChemExpress, HY-17409), chlorpromazine (MedChemExpress, HY-12708), dynasore (MedChemExpress, HY-15304), αCD71 IgG (Abcam, ab214039), or cytochalasin D (MedChemExpress, HY-N6682). After incubation in a constant temperature cell incubator at 37 °C and 5% CO_2_ for 30 min, the cells were washed three times with PBS. Each well was added with pFn+@GFP, and after incubation with the cells for different times, the cells were washed three times with PBS to remove the proteins that were not taken up. The adherent cells were digested with trypsin, the cells were resuspended in PBS, and then the cells were collected by centrifugation at room temperature at 1000 × *g*, washed three times with PBS, and a single cell suspension was prepared. The effect of different inhibitors on the cellular uptake efficiency of pFn+@GFP was detected by flow cytometry, and the cellular uptake mechanism was analyzed.

### Preparation and characterization of supercharged cavity proteins and protein complex

To verify the universality of pFn+ in delivering proteins, we selected a range of proteins with different physicochemical properties and biological activities, including GFP (MedChemExpress, HY-P71461), β-Gal (Beyotime, RG0039), HRP (Abcam, ab7403), Saporin (Sigma-Aldrich, S9896), αGFP IgG (Proteintech, 66002-1-Ig), αALK IgG (Proteintech, 60321-1-Ig), αLamin-B1 IgG (Abcam, ab16048), Cas9 proteins (Beyotime, D0511M) and Cas9-NLS proteins (Beyotime, D0513M). Initially, the target protein was gently added to an appropriate concentration of pFn+ and incubated at room temperature for 30 min. During this period, the suspension was gently pipetted to ensure the formation of uniform complex. Subsequently, the size and zeta potential of the complex were measured using a Zetasizer Nano ZS90 (Malvern Instruments, UK), and their morphology was observed with a HT7700 microscope (Hitachi, Japan).

### In vitro delivery of Cas9/sgRNA complex

*Cleavage activity assay*: the sgRNA at 30 nM was incubated with 300 nM Cas9 protein in an EP tube at room temperature for 30 min, followed by the addition of 2 μg of PCR-amplified GFP gene fragments. The mixture was then subjected to a reaction in a 37 °C metal bath for 1.5 h, followed by 2% (w/v) agarose gel electrophoresis to detect the in vitro cleavage activity of the Cas9/sgRNA complex. The sgRNA, synthesized by GenScript, was used for gene editing of the GFP gene, with the specific sequence being: 5′-CAG CAG AAC ACC CCC AUC GGC GUU UUA GAG CUA GAA AUA GCA AGU UAA AAU AAG GCU AGU CCG UUA UCA ACU UGA AAA AGU GGC ACC GAG UCG GUGC-3′. To obtain the target gene GFP for in vitro cleavage activity testing, the GFP gene fragment was amplified from a plasmid expressing GFP using PCR. The PCR reaction system and primer sequences are as follows: GFP-F: 5′-ATG GTG AGC AAG GGC GAGG-3′ GFP-R: 5′-CTT GTA CAG CTC GTC CAT GCC GA-3′.

*Delivery and localization analysis of Cas9 protein*: the Cas9-NLS protein, tagged with a nuclear localization sequence at the C-terminus, can be localized within the nucleus after being taken up by cells. MDA-MB-231 cells were seeded in 35 mm laser confocal glass-bottom culture dishes, covering 15% of the bottom area. Three hundred microliters of the cell suspension was added to the glass-bottom imaging center, gently shaken, and then cultured overnight at 37 °C and 5% CO_2_. The next day, when the cell coverage area reached 30–40%, pFn+@Cas9 (molar ratio of 1:8 for pFn+: Cas9, 1 μM Cas9) was added to the dish and incubated for 24 h. Cells were then washed three times with PBS to remove uninternalized proteins. The cell nuclei were stained with 5 μg/mL Hoechst 33342 staining solution, protected from light, and stained for 20 min in a 37 °C, 5% CO_2_ incubator. After washing three times with PBS, the intracellular localization of the pFn+@Cas9-delivered Cas9 protein was observed using a laser confocal microscope.

*Flow cytometry for assessing gene editing efficiency*: HEK-293T-GFP cells were seeded in a 24-well plate and incubated for 24 h. The cells were then treated with RNP, pFn + 216, PULSin@RNP, CMAX@RNP, and pFn+216@RNP, the molar ratio of 1:8 for pFn + 216:RNP, followed by overnight incubation. The medium was replaced with fresh medium containing 10% FBS. After an additional 48 h of incubation at 37 °C. Cells were washed three times with PBS to remove unincorporated proteins, digested with trypsin, resuspended in PBS, and then centrifuged at 1000 × *g* at room temperature to collect the cells. After three washes with PBS, a single cell suspension was prepared. Flow cytometry was used to measure the pFn+216@RNP to evaluate the gene editing efficiency in cells.

*T7E1 assay*: HEK-293T-GFP cells were seeded in a 6-well plate, covering 30% of the bottom area, gently shaken, and then cultured overnight at 37 °C and 5% CO_2_. The next day, pFn+@Cas9/sgRNA was added and incubated for 24 h. Cells were washed with PBS to remove uninternalized proteins, then the medium was replaced with fresh complete culture medium and continued to culture for another 48 h. After culturing, cells were digested with trypsin, centrifuged to enrich, and genomic DNA was extracted using a genomic DNA extraction kit. The GFP gene was amplified from the genomic DNA, and the gene editing efficiency of the Cas9/sgRNA delivered into the cells was detected using the T7E1 assay.

*In vitro sequencing*: HEK-293T-GFP cells were seeded in a 24-well plate and incubated for 24 h. The cells were then treated with pFn+216@RNP, followed by overnight incubation. The medium was replaced with fresh medium containing 10% FBS. After an additional 48 h of incubation at 37 °C, the cells were collected and processed using a universal genomic DNA kit (Tiangen, China) to harvest genomic DNA. The sgRNA-targeted genomic loci were amplified, purified through gel extraction (Vazyme, China), and subsequently analyzed using T7E1 cleavage assay. Briefly, 200 ng of the purified polymerase chain reaction (PCR) product was denatured and reannealed in 2 μl of NEBuffer 2 (10×) using the following protocol: 95 °C, 5 min; 95° to 85 °C, −2 °C/s; 85° to 25 °C, −0.1 °C/s; and then held at 4 °C. Then, 1 μl of T7E1 (NEB, China, B7002S) was added to the annealed PCR products and incubated at 37 °C for 1 h. Products were analyzed on 2% agarose gels and imaged with a Chemiluminescence imaging system (Tanon). PCR products with mutations indicated by the T7E1 assay were subjected to DNA sequencing and subcloned into T-clone vectors (Vazyme, China). Colonies were picked randomly and further analyzed by Sanger sequencing using an M13F primer (Sangon Biotech).

### Intracellular delivery of antibody/Trim21 complex

For the preparation of the Trim-away complex, an equimolar ratio of Trim21 protein (MedChemExpress, HY-P71791) and GFP antibody were incubated at room temperature for 30 min, with gentle vortexing to ensure thorough mixing of the system. HEK-293T-GFP cells were seeded in a 35 mm laser confocal glass-bottom dish covering 15% of the bottom area. After adding 300 μL of cell suspension to the glass-bottom imaging center and gently shaking, the cells were incubated overnight at 37 °C and 5% CO_2_. The pFn+@Trim-away was added to the dish, with separate pFn+@Trim-away and the commercial protein transfection reagent PULSin used as controls. After incubation for 24 h at 37 °C and 5% CO_2_, uninternalized proteins were washed off with PBS. Cells were stained with 5 μg/mL Hoechst 33342 live-cell dye for 20 min in the dark at 37 °C and 5% CO_2_. The samples were gently washed three times with sterile PBS, each for 5 min, to remove residual staining solution, and the change in green fluorescence intensity in HEK-293T-GFP cells was observed to verify the GFP knockdown capability of pFn+@Trim-away. Cells were collected and analyzed to assess changes in GFP protein levels in different groups of HEK-293T-GFP cells, further verifying the knockdown capability of pFn+@Trim-away.

### In vivo mouse models and treatments

Balb/c mice used in this study were housed under specific pathogen-free (SPF) conditions with a 12/12-h light/dark cycle, relative humidity of 45-55% and controlled room temperature of 22–25 °C. All animals received humane care according to the criteria outlined in the Guide for the Care and Use of Laboratory Animals. Animal protocols were approved by the Ethics Committee of China Pharmaceutical University (the ethics approval number: 2023-06-023). According to national and institutional guidelines, the maximum tumor volume and weight allowed was 1.5 cm^3^ and 10% of the body weight of the mouse. Mice were euthanized when the tumor burden exceeded the threshold. For the H3122/Ale subcutaneous tumor model, female mice (6–8 weeks) were inoculated with H3122/Ale cells (1 × 10^6^) in the right inguinal region. When the tumor size was about 100 mm^3^, it was recorded as day 0. The tumor-bearing Balb/c mice were randomly assigned to groups for in vivo anti-tumor studies. The body weight of mice in each group was maintained at the same level as that of mice in the same batch. Mice were administered intratumoral injections of saline, pFn + 216, Trim21/αALK, PULSin@Trim21/αALK, or pFn+216@Trim21/αALK complex per mouse (equivalent to 1 μg αALK IgG) every 4 days. In the pFn+216@Trim21/αALK complex, the molar ratio of the three components is 1:8:8 (pFn + 216:Trim21:IgG). The Alectinib group was treated with oral Alectinib (20 mg/kg/day). In the process of treatment, the body weight and tumor volume of the mice were measured every 2 days, calculating formula for Volume = Wildth^2^ × Length/2. After 28 days of treatment, mice were euthanized, tumors were harvested for subsequent assays.

### In vivo delivery of Cas9/sgRNA complex

*In vivo imaging*: Ai14 female mice (6–8 weeks) were intravenously injected via the tail vein with PBS, RNP, PULSin@RNP, or pFn+216@RNP. The Cas9 protein was labeled with Cy7 for tracking purposes. Luminescence intensity was monitored at various time points using the IVIS imaging system. Twenty-four hours post-injection, the mice were euthanized, and major organs (heart, liver, spleen, lungs, and kidneys) were harvested for ex vivo fluorescence imaging using the IVIS near-infrared fluorescence imaging system.

*Flow cytometry*: to evaluate the drug uptake capacity of different liver cell populations, primary hepatocytes were isolated from the livers of treated mice. The cells were stained with PE‑conjugated anti‑ASGR1 antibody (Proteintech, PE-11739,1 µL/test), PE‑conjugated anti‑F4/80 antibody (BD Biosciences, 565410,5 µL/test), and FITC‑conjugated anti‑CD31 antibody (BioLegend, 102405,2 µL/test) for 30 min, followed by analysis using flow cytometry and FlowJo software.

*In vivo gene editing*: the Cas9 nuclease was complexed with synthetic sgRNA to form RNP complexes targeting the excision of the SV40 polyA sequence. The pFn+216@RNP formulation was prepared as described previously for intravenous administration. Four groups of Ai14 mice (*n* = 3 per group) were injected with 100 μL of either PBS (negative control), RNP, PULSin@RNP, or pFn+216@RNP solutions (6 mg Cas9 equivalent/kg). TdTomato expression in the organs and tissues was initially quantified using the IVIS system with an excitation wavelength of 554 nm and an emission wavelength of 581 nm. Subsequently, the mice were euthanized, and their major organs and tissues (heart, liver, spleen, lungs, and kidneys) were either fixed in 4% neutral buffered formalin and stored at 4 °C or snap-frozen in liquid nitrogen and maintained at −80 °C for further analysis.

*Immunofluorescence staining*: TdTomato expression was further investigated via immunofluorescence staining. The collected tissues were embedded in optimal cutting temperature compound blocks, which were then sectioned into 5 µm‑thick slices. Sections were stained with αRFP primary antibody (Abcam, ab152123, 1:1000) for tdTomato. Hepatocytes were stained using αHSA antibody (Abcam, ab191200, 1:100). Endothelial cells were stained with an αCD31 antibody (Abcam, ab56299, 1:400). Macrophages/Kupffer cells were stained with an αF4/80 antibody (Abcam, ab6640, 1:100). All sections were further incubated with secondary antibodies (1:1000) and DAPI (1:1000). After mounting on slides, the sections were imaged under a microscope.

### Statistics and reproducibility

Confocal microscopy images of cellular uptake and colocalization (Fig. 4A, D) were repeated five times independently with similar results, and representative images from each group were shown. Cell staining images for β-galactosidase activity and immunostaining images (Fig. 5A, B, J) as well as T7E1 assay images (Fig. 5G) were repeated five times independently with similar results, and representative images from each group were shown. Immunofluorescence staining of liver sections (Fig. 6H–J) was repeated five times independently with similar results, and representative images from each group were shown. Confocal microscopy images of intracellular antibody delivery (Fig. 7B) were repeated five times independently with similar results, and representative images from each group were shown.

### Statistical data analysis

All quantitative data are expressed as the mean ± SEM. GraphPad Prism 10.5 (GraphPad Software Inc.) was used for statistical analyses. Declared group size (*n*) refers to the number of independent values rather than technical replicates, and statistical analyses were undertaken only where group size was at least *n* = 3. The group size is the number of independent values, and statistical analysis was done using these independent values, and there were no outliers in the data analysis. Unpaired two-tailed Student’s *t* test for comparisons between two groups, one-way or two-way ANOVA with Dunnett’s, Tukey’s, or Sidák’s multiple comparisons test for multi-group analyses.

### Reporting summary

Further information on research design is available in the Nature Portfolio Reporting Summary linked to this article.

## Supplementary information

Supplementary Information

Description of Additional Supplementary Files

Supplementary Movie 1

Reporting Summary

Transparent Peer Review File

## Source data

Source Data

## Data Availability

All relevant data supporting the findings of this study are available within the article and its Supplementary Information files. The Source data underlying Figs. 2–7 and Supplementary Figs. 2–16, 18, 20–27 are provided as a Source data file. Source data are provided with this paper.
