# Supplementary material for: Supercharged ferritin nanocages enable universal cytosolic protein delivery
Source: Nat Commun. 2026 Jun 9;17:7319. doi: 10.1038/s41467-026-74247-x (PMC13402336; doi:10.1038/s41467-026-74247-x)
Supplement: Supplementary file 1 — Supplementary Information [file 41467_2026_74247_MOESM1_ESM.pdf]

Supplementary information

Supercharged ferritin nanocage enable universal cytosolic protein delivery

*Dingkang Liu[#1,2], Hong Luo[#1], Qingzhou Lu[#1], Lichao Yu[1], Minjiang Chen[3], Wenbing Yao[1], Pan Hu[2], Lubin Liu[2], Wei Liu[*1], Xiangdong Gao[*1], Jiansong Ji[*3], Jun Yin[*1,3]*

[1]Jiangsu Key Laboratory of Druggability of Biopharmaceuticals and State Key Laboratory of Natural Medicines, School of Life Science and Technology, China Pharmaceutical University, Nanjing 210009, China.
[2]Department of Obstetrics and Gynecology, Women and Children's Hospital of Chongqing Medical University, No.120 Longshan Road, Yubei District, Chongqing, 401147, China.
[3]Zhejiang Key Laboratory of Imaging and Interventional Medicine, Zhejiang Engineering Research Center of Interventional Medicine Engineering and Biotechnology, the Fifth Affiliated Hospital of Wenzhou Medical University, Lishui 323000, China.

*Corresponding authors:
Jun Yin, e-mail: junyin@cpu.edu.cn., telephone: +86-13770339132., we designate this one to further communicate with the Editorial and Production offices.
Jiansong Ji, e-mail: jjstcty@wmu.edu.cn.
Xiangdong Gao, e-mail: xdgao@cpu.edu.cn.
Wei Liu, e-mail: liuwei@cpu.edu.cn.

[#]These authors made equal contributions to this work.

Supplementary Methods

Identification of Recombinant Proteins

Size Exclusion Chromatography-High Performance Liquid Chromatography (SEC-HPLC): Purified protein samples were filtered using a 0.22 μm filter head and used for SEC-HPLC analysis. The target protein was diluted to 0.5 mg/mL, and the Zenix-C 300 column (Sepax Technologies, USA) was used to analyze the hydrodynamic volume and purity of the protein. The mobile phase was a pH 8.0, 50 mM Tris-HCl buffer, the flow rate was set to 0.5 mL/min, and the sample detection time was 40 min. The standard protein was used as a control sample to determine the peak time, and the standard curve was obtained by plotting against the corresponding standard protein molecular weight. The peak time of the target protein was substituted into the standard curve to calculate the hydrodynamic volume of this protein.

Dynamic Light Scattering (DLS): Purified protein samples were diluted to a total volume of 1 mL of 0.3 mg/mL solution, used for DLS determination of the hydration hydrodynamic diameter and surface potential. When measuring the hydration hydrodynamic diameter, the protein sample was slowly added to a clean quartz colorimetric dish to ensure no bubble formation, and then the Zetasizer Nano ZS90 (Malvern Instruments, UK) was used to detect the particle size of the complex. Measurements were taken three times, each time for 10 s. When measuring the surface potential of nanoparticles, 1 mL of protein sample was slowly added along one end of the potential dish, ensuring no bubbles and that both ends of the electrode were submerged and the liquid level was flat, then the potential was measured.

Transmission Electron Microscopy (TEM): Purified protein samples were diluted to a total volume of 10 μL of 0.3 mg/mL solution, used for observation and detection by TEM. Firstly, a small copper mesh used as a carrier was carefully picked up with tweezers, then one side was lightly pressed down on the copper mesh, and the protein sample was added dropwise from above, ensuring that the copper mesh was immersed in the protein solution, and incubated for 5 min. Then, the protein solution was absorbed from the side using blotting paper, phosphomolybdic acid staining solution was added for staining for 3 min, and then the staining solution was absorbed from the side using blotting paper. The staining operation was repeated once, and after absorbing the staining solution, the sample was dried using a drying lamp and observed under a HT7700 microscope (Hitachi, Japan).

Study on the Cellular Uptake Efficiency of Fluorescent Proteins

Fluorescence Spectral Scanning of the Complex: The Infinite M200 fluorometer (Tecan Trading AG, Switzerland) was used to detect the fluorescence spectra of the complex formed by pFn+ and GFP. A solution containing 0.5 mg/mL GFP in the complex was taken in a volume of 200 μL and placed in a black 96-well plate to measure its fluorescence absorption and emission spectra. A solution of GFP at 0.5 mg/mL served as the control. The spectra were plotted with wavelength as the x-axis and absorption or emission intensity as the y-axis to compare the impact of the formation of the pFn+@GFP on the fluorescence characteristics of GFP.

Study of Cellular Uptake Efficiency: MDA-MB-231 cells were seeded in a 24-well plate covering 30% of the bottom area and gently shaken before being incubated overnight at 37°C and 5% CO_2. The next day, supercharged cavity protein and GFP were incubated in an EP tube for 30 min, then added to the cells and incubated for 12 h. PULSin was used as a positive control for the delivery of fluorescent proteins. Cells were washed three times with PBS to remove unincorporated proteins, digested with trypsin, resuspended in PBS, and then centrifuged at 400 x g at room temperature to collect the cells. After three washes with PBS, a single cell suspension was prepared. Flow cytometry was used to measure the cellular uptake efficiency of pFn+@GFP.

Intracellular Localization Study: Confocal laser scanning microscopy (CLSM) was performed using LSM800 (Zeiss, Germany). Endosomal markers were used to explore the changes in the intracellular transport pathways during the intracellular delivery process of pFn+@GFP. MDA-MB-231 cells were seeded in a 35 mm laser confocal glass-bottom culture dish covering 15% of the bottom area. After gently shaking, 300 μL of the cell suspension was added to the center of the glass bottom and incubated overnight at 37°C and 5% CO_2. The pFn+@GFP was prepared, and the pFn+@GFP was added per well and incubated with the cells for 12 h. Cells were washed three times with PBS to remove unincorporated proteins. Immunofluorescence staining was used to identify the colocalization of intracellular GFP with lysosomes using the Lysotracker Red.

The effect of serum on the complex formed by supercharged cavity protein and fluorescent protein

MDA-MB-231 cells was seeded in a 24-well plate covering 30% of the bottom area. The cells were gently shaken and then incubated overnight at 37°C and 5% CO_2. Next day, when the cells covered 60% to 70% of the plate area, the cell culture medium was replaced with Opti-MEM, complete medium containing 10% (*v/v*) FBS, or complete medium containing 100% (*v/v*) FBS. Each well was then added with the pFn+@GFP, and incubated with the cells for 12 h. After incubation, the cells were washed three times with PBS to remove unabsorbed proteins. The adherent cells were then digested with trypsin, resuspended in PBS, and centrifuged at 400 x g at room temperature to collect the cells. The cells were washed three times with PBS and resuspended to form a single-cell suspension. Flow cytometry was used to detect the effect of serum content in the culture medium on the cellular uptake efficiency of pFn+@GFP.

Intracellular Delivery and Activity Assay of β-Galactosidase

β-Galactosidase can react with the substrate X-Gal to produce a deep blue product, making it easy to observe the blue-stained cells under an optical microscope. The process for intracellular delivery and activity detection is as follows: The prepared pFn+@β-Gal (molar ratio of 1:8 for pFn+:β-Gal, 1 μM β-gal) needs to be tested to assess the impact of the complex on enzyme activity. A β-Gal activity assay kit was used to measure the activity of β-Gal within the complex and evaluate the effect of pFn+ on β-Gal enzyme activity. MDA-MB-231 cells were seeded in a 24-well plate covering 30% of the bottom surface area, gently shaken, and then incubated overnight at 37°C with

5% CO_2. Next day, the pFn+@β-Gal was added to the 24-well plate, along with separate β-Gal and the commercial protein transfection reagent PULSin as controls. The cells were incubated for 24 h at 37°C with 5% CO_2, after which uninternalized proteins were washed away with PBS. Then, 500 μL of β-Gal staining solution was added, and cells were fixed at room temperature for 10 min. The supernatant was then removed, and the cells were washed three times with PBS, 3 min each time. After removing the PBS, 500 μL of staining working solution was added to each well. The staining of the cells was observed using an optical microscope.

Intracellular Delivery and Activity Detection of Horseradish Peroxidase

Horseradish peroxidase (HRP) can also react with substrates to produce color, and its intracellular delivery and activity detection process are as follows: The pFn+@HRP (molar ratio of 1:8 for pFn+:HRP, 1 μM HRP) was prepared to examine its impact on enzyme activity using an HRP activity assay kit to evaluate the influence of pFn+ on HRP enzyme activity. MDA-MB-231 cells were seeded in a 24-well plate covering 30% of the bottom area, gently shaken, and then incubated overnight at 37°C and 5% CO_2. The next day, the pFn+@HRP was added to the 24-well plate, along with separate HRP and the commercial protein transfection reagent PULSin as controls and incubated for 24 h at 37°C and 5% CO_2. After incubation, uninternalized proteins were washed off with PBS. 500 μL of 4% paraformaldehyde was added to fix the cells at room temperature for 20 min. The supernatant was then aspirated, and the cells were washed three times with PBS, each wash lasting 3 min. After aspirating the PBS, 500 μL of DAB working solution was added to each well and incubated for 5 min. Cells were then observed under an optical microscope for staining. Then, cells were again fixed with 500 μL of 4% paraformaldehyde at room temperature for 20 min. After removing the supernatant and washing the cells three times with PBS, 500 μL of TMB working solution was added to each well and incubated for 10 min. The reaction was stopped with a stop solution, and the absorbance of the samples at 450 nm was measured with a Spectra Max I3X microplate reader (Molecular devices, USA).

Intracellular Delivery and Activity Assay of Toxic Proteins Saporin and RNase A

The process for the intracellular delivery and activity assay of the toxic proteins Saporin and RNase A is as follows: MDA-MB-231 cells were seeded in a 96-well plate covering 30% of the bottom area. The cells were gently shaken and then incubated overnight at 37°C and 5% CO_2. Then, add complex of pFn+@Saporin (molar ratio of 1:8 for pFn+:Saporin, 1 μM Saporin) or pFn+@RNase A (molar ratio of 1:8 for pFn+:RNase A, 1 μM RNase A) to the 96-well plate. As controls, use Saporin or RNase A alone and a commercial protein transfection reagent PULSin. Incubate at 37°C and 5% CO_2, then wash off uninternalized proteins with PBS. Replace the medium with complete medium containing 10% (*v/v*) FBS and continue to incubate for 24 h at 37°C and 5% CO_2. Add 10 μL of 5 mg/mL MTT solution to each well and incubate for an additional 4 h. Following this, introduce 100 μL of DMSO to each well, ensuring thorough mixing before further incubation. After gently shaking for 10 min, confirm that the purple precipitate has dissolved by examining under a

standard optical microscope. Finally, measure the absorbance at 570 nm using a Spectra Max I3X microplate reader (Molecular Devices, USA) to evaluate cell viability post-treatment.

Intracellular delivery of the antibody IgG

The antibody IgG (Huabio, China, Catalog: HA1027) was labeled with FAM fluorophore. pFn+@IgG (molar ratio of 1:8 for pFn+:IgG, 1 μM IgG) was added to a 24-well plate, alongside standalone IgG and the commercial protein transfection reagent PULSin as controls. The samples were incubated in a constant temperature incubator at 37°C with 5% CO_2 for 24 h, after which any uninternalized protein was washed off with PBS. Cells in the 24-well plate were digested with trypsin. After digestion was terminated, cells were collected by centrifugation and washed three times with PBS. The cell uptake efficiency of the pFn+@IgG was then analyzed using flow cytometry after filtering. Lamin-B1, a type of nuclear lamina protein primarily located on the nuclear membrane, was used to verify the intracellular delivery and nuclear membrane localization capability of αLamin-B1 (Huabio, China, Catalog: HA601289) following delivery via pFn+. Initially, αLamin-B1 was labeled with FITC. MDA-MB-231 cells were seeded in a 35 mm laser confocal glass-bottom dish covering 15% of the bottom area. 300 μL of the cell suspension was gently added to the center of the glass-bottom for imaging, and the cells were gently shaken before being placed in a constant temperature incubator at 37°C with 5% CO_2 overnight. pFn+@αLamin-B1 was then added to the dish. Controls include standalone αLamin-B1 and the commercial protein transfection reagent PULSin. After incubating for 24 h at 37°C with 5% CO_2, uninternalized protein was washed off with PBS. Cells were stained with 5 μg/mL Hoechst33342 live-cell nuclear stain, kept in the dark, and stained for 20 min in the constant temperature incubator. The samples were gently washed three times with sterile PBS, each for 5 min, to remove any residual stain. The antigen recognition ability of αLamin-B1 in living cells was then observed using a laser confocal microscope.

Preliminary Study on the Biosafety of Supercharged Cavity Proteins

Cytotoxicity assay: MDA-MB-231, C6, A549, PC12, BEL-7402 and Jurkat cells were seeded in a 96-well plate covering 30% of the bottom area. The cells were gently shaken and then incubated overnight at 37°C with 5% CO_2. Various concentrations of pFn+ were added to the 96-well plate and incubated for 24 h. Then, 10 μL of 5 mg/mL MTT solution was added to each well and incubated for another 4 h. Subsequently, 100 μL of DMSO was added to each well, mixed appropriately, and incubated with occasional shaking for 10 min until the purple precipitate was completely dissolved under a conventional optical microscope. The absorbance at 570 nm was measured to assess the cytotoxicity using a Spectra Max I3X microplate reader (Molecular Devices, USA).

Hemolytic activity assay: Blood was collected from healthy BALB/c mice via eyeball removal into anticoagulant tubes, then transferred to a triangular flask containing glass beads and gently shaken for 15 min to defibrinate the blood. After washing with PBS and centrifuging at 400 x g for 15 min at room temperature, the enriched red blood cell pellet was collected and gently washed with PBS

until the supernatant showed no signs of hemolysis. The red blood cells were resuspended in PBS to prepare a 2% (*v/v*) working solution. Different concentrations of pFn+, along with PBS and 0.1% (*v/v*) Triton X-100 as controls, were added to the red blood cell working solution. After mixing, the samples were incubated in a 37°C water bath, and hemolysis was observed and recorded periodically. The absorbance of each sample at 540 nm was measured using a Spectra Max I3X microplate reader (Molecular Devices, USA) to determine the extent of red blood cell destruction and calculate the hemolytic activity of pFn+.

Transcriptome analysis: For each sample, total RNA was extracted with TRIzol reagent (Invitrogen, China, Catalog: 15596026CN). Sequencing libraries were prepared using 1 μg of total RNA with a RIN value of 7 or higher. Next-generation sequencing library preparation was performed under the manufacturer's protocol (NEBNext® Ultra™ RNA Library Prep Kit for Illumina®). Libraries with different exponents were then multiplexed and imported onto the Illumina HiSeq instrument following the instructions provided. Sequencing was performed using 2×150 bp paired ends (PE); analysis of images and bases was performed on HiSeq Control Software (HCS) + OLB + GAPipeline-1.6 (Illumina). Sequences were processed and analyzed by Azenta Life Science (Suzhou, China). The raw RNA-seq data was uploaded to NCBI BioProject database (Accession number: PRJNA1294381).

***In vivo* safety assessment:** BALB/c mice were randomly allocated into four groups and administered pFn+216 daily via intraperitoneal injection at doses of 0, 1, 5, and 25 mg/kg for 14 consecutive days. On day 15, all mice were euthanized. Peripheral blood was collected, and serum samples were obtained for cytokine analysis. Levels of IL-6 (Catalog: EM0004), IL-1β (Catalog: EM0029), and TNF-α (Catalog: EM0010) were quantified using commercial ELISA kits (Huabio, China) according to the manufacturer's instructions. Major organs were harvested, fixed in 4% paraformaldehyde, embedded in paraffin, and subjected to histological examination by hematoxylin and eosin (H&E) staining.

Establishment of the H3122/Ale cell line

The parental H3122 cells were seeded into a 12-well plate and grown to approximately 90% confluence, and the first dose of the chemotherapeutic drugs was then applied at a concentration of 1 nM. The medium was changed after 4 h of drug exposure, and the cells that were alive 24 h after the removal of the drug were considered resistant cells. The chemotherapeutic drug-resistant cells were collected and cultured until they resumed proliferating and established stable clones. The drug-resistant cells were further inoculated into a 12-well plate, and the above process was performed until the cells no longer exhibited significant apoptosis during exposure to increasing concentrations of the drugs (the concentration of the drug was increased by 50 nM).

Supplementary Figures

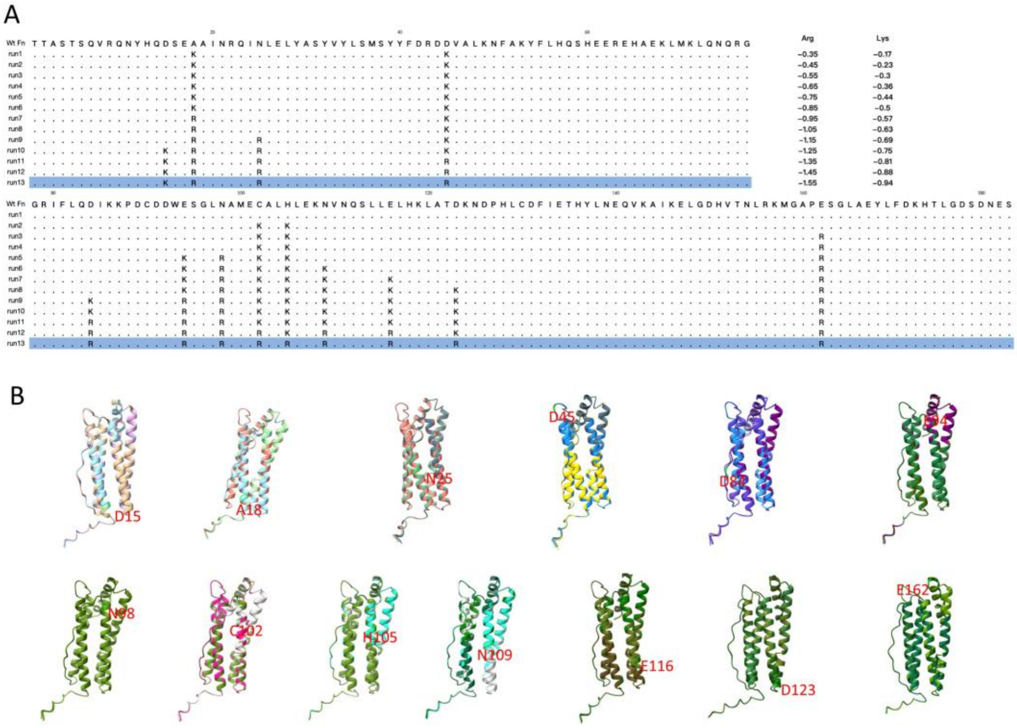

C

Site / RMSD	D15	A18	N25	D45	D84	E94	N98	C102	H105	N109	E116	D123	E162
R	0.379	0.095	0.168	0.155	0.133	0.130	0.121	0.107	0.111	0.115	0.077	0.122	0.133
K	0.371	0.129	0.205	0.194	0.099	0.152	0.158	0.094	0.089	0.110	0.114	0.095	0.162

Supplementary Fig. 1 Surface mutation site predicting for Fn nanocage.

A. Potential mutation sites were found using the Rosetta fixbb module. The reference energy weights for Arg and Lys were set to different values for each run (as shown to the right of the sequence) to create sequences with increasing numbers of positively charged residues. For each run, the sequence with the lowest total Rosetta energy is shown. The 13 potential mutation sites ultimately identified are highlighted in blue. **B.** Structural representations of single mutations at different sites of Arg and Lys predicted by AlphaFold2. **C.** The R and K mutations at the same mutation site were aligned with the original structure using the Matchmaker tool in Chimera X to calculate the Root Mean Square Deviation (RMSD). The values of R and K at the mutation site were determined by selecting the smaller RMSD value.

A

Fn	Mutation site	Surface net charge
WT Fn	Wild Type	-168
Fn-120	D15K	-120
Fn-72	D15K/D45R	-72
Fn-24	D15K/D45R/D84K	-24
Fn+24	D15K/D45R/D84K/E94R	+24
Fn+72	D15K/D45R/D84K/E94R/E116R	+72
Fn+120	D15K/D45R/D84K/E94R/E116R/D123K	+120
Fn+168	D15K/D45R/D84K/E94R/E116R/D123K/E162R	+168
Fn+192	D15K/D45R/D84K/E94R/E116R/D123K/E162R/A18R	+192
Fn+216	D15K/D45R/D84K/E94R/E116R/D123K/E162R/A18R/N25R	+216
Fn+240	D15K/D45R/D84K/E94R/E116R/D123K/E162R/A18R/N25R/N98R	+240
Fn+264	D15K/D45R/D84K/E94R/E116R/D123K/E162R/A18R/N25R/N98R/C102K	+264
Fn+288	D15K/D45R/D84K/E94R/E116R/D123K/E162R/A18R/N25R/N98R/C102K/H105K	+288
Fn+312	D15K/D45R/D84K/E94R/E116R/D123K/E162R/A18R/N25R/N98R/C102K/H105K/N109K	+312

B

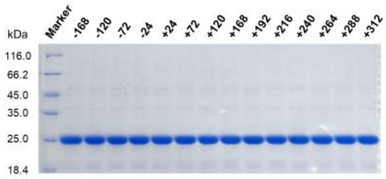

C

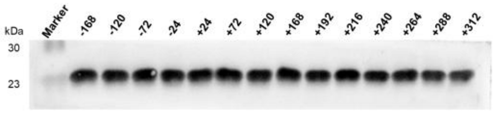

Supplementary Fig. 2 Construction and purification of Fn nanocage.

A. The mutation sites of Fn+ and the calculated theoretical net surface charge. **B.** Fn-168 to Fn+312 were analyzed through SDS-PAGE. **C.** Western blot analysis for protein identification. The PVDF membrane was stained with anti-Fn antibody to confirm the identity of ferritin protein.

A

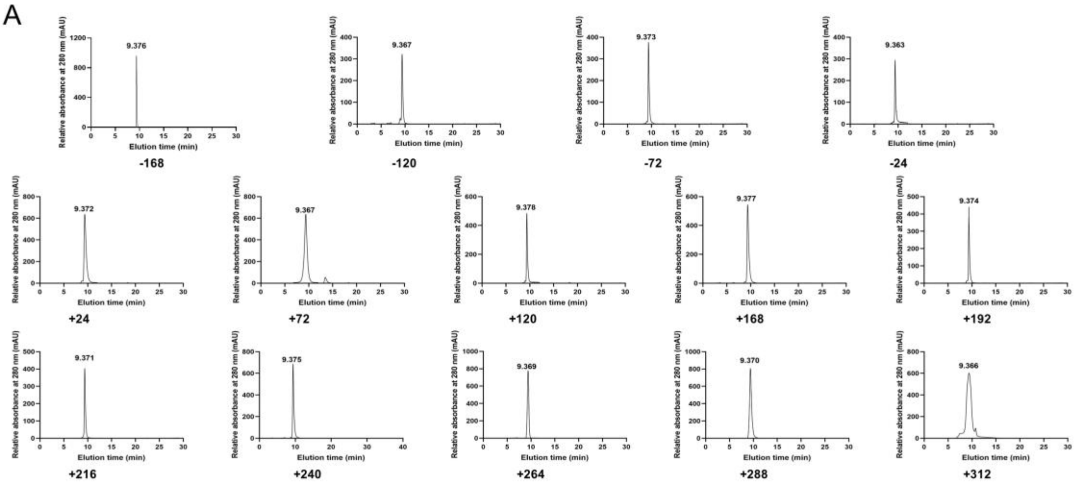

B

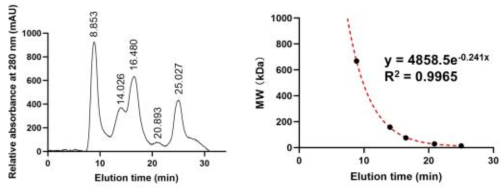

Standard	Elution time (min)	Theoretical MW (kDa)
Thyrogloubin	8.853	669
γ-globulin	14.026	158
Conalbumin	16.480	75
Carbonic anhydrase	20.893	29
Ribonuclease A	25.027	13.7

C

Sample	Elution time (min)	Apparent MW (kDa)
-168	9.376	507.18
-120	9.367	508.28
-72	9.373	507.55
-24	9.363	508.77
+24	9.372	507.67
+72	9.367	508.28
+120	9.378	506.94
+168	9.377	507.06
+192	9.374	507.43
+216	9.371	507.79
+240	9.375	507.30
+264	9.369	508.04
+288	9.370	507.92
+312	9.366	508.41

Supplementary Fig. 3 SEC-HPLC analysis of Fn-168 to Fn+312.

A. The purity and apparent molecular weight of Fn-168 to Fn+312 were analyzed using SEC-HPLC in a 150 mM phosphate buffer (pH 7.4). **B.** SEC-HPLC of standard proteins. The relationship between the elution time and the labeled proteins (thyroglobulin 670 kDa, BSA 66 kDa, ovalbumin 44 kDa, ribonuclease A 13.7 kDa, and vitamin B12 1.35 kDa) was established and fitted using a nonlinear regression model. **C.** A list of calculated apparent molecular weights for Fn-168 to Fn+312.

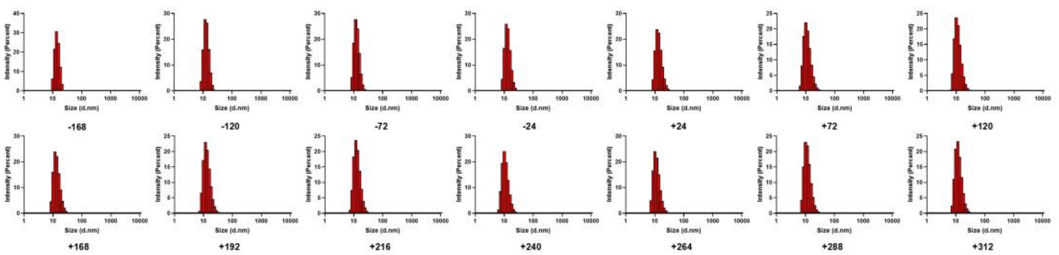

Supplementary Fig. 4 Particle size and morphology of Fn-168 to Fn+312.

The figure presents a panel of dynamic light scattering (DLS) intensity distributions for engineered ferritin (Fn) nanocage variants in 150 mM sodium phosphate buffer (pH = 7.4).

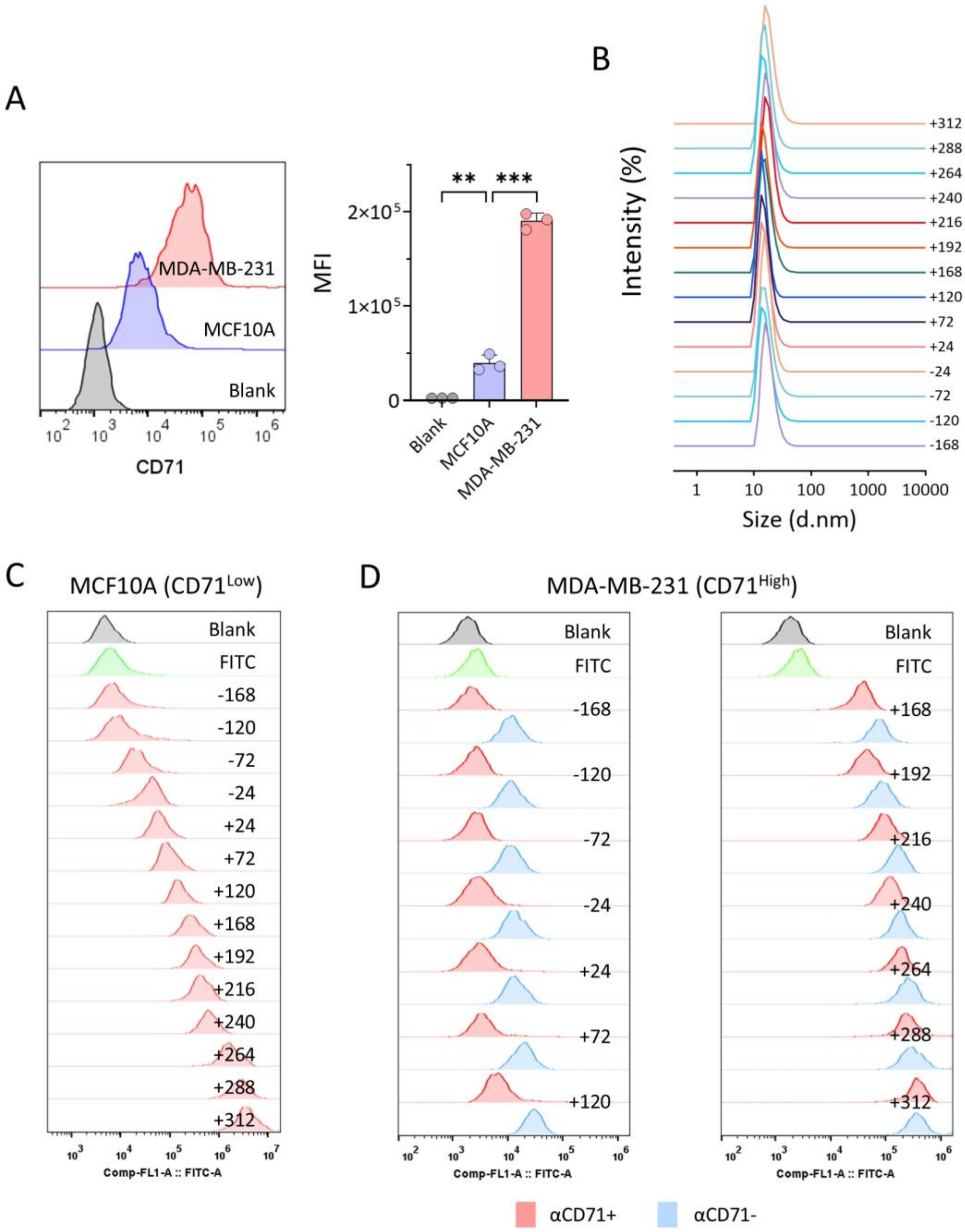

Supplementary Fig. 5 The relationship between Fn+ cellular uptake and CD71 expression. **A.** The expression levels of CD71 in MCF10A and MDA-MB-231 cells (n=3 independent samples). Data are presented as mean ± SEM; statistical significance among groups was determined by one-way ANOVA with Tukey's multiple comparisons test (**P < 0.01, ***P < 0.001). **B.** Particle size of FITC-labeled Fn+ was detected by DLS. **C.** Cellular uptake efficiency of Fn+. FITC-labeled proteins at 1 μM were incubated with MCF10A cells at 37°C for 12 h. The MFI was analyzed using flow cytometry. **D.** Cellular uptake efficiency of Fn+. FITC-labeled proteins at 1 μM were incubated with MDA-MB-231 cells at 37°C for 12 h. The CD71 receptor was blocked by using an anti-CD71 antibody, and the MFI was analyzed using flow cytometry.

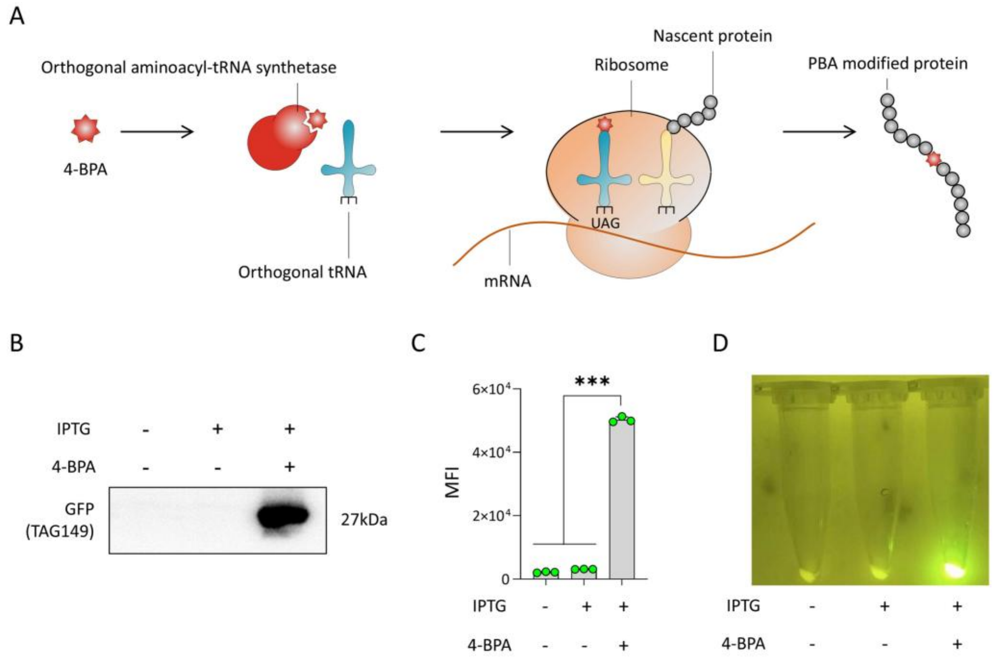

Supplementary Fig. 6 Evaluation of PBA incorporation efficiency via GFP(TAG149).

A. To incorporate the unnatural amino acid 4-boronophenylalanine (4-BPA) into the protein, 4-BPA is specifically recognized and attached by an "orthogonal" aminoacyl-tRNA synthetase to an orthogonal amber suppressor tRNA. This tRNA is decoded at the introduced amber codon (UAG) during the translation process, facilitating the encoding of 4-BPA into the supercharged ferritin. **B.** Immunoblot analysis of GFP (TAG149) incorporating PBA using the pMjTyrRS/tRNA. **C-D.** Comparison of GFP fluorescence intensity in BL21 (DE3) expressing strains with and without the addition of PBA (n=3 independent samples). Data are presented as mean ± SEM; statistical significance was determined using one-way ANOVA followed by Dunnett's multiple comparisons test (***P < 0.001).

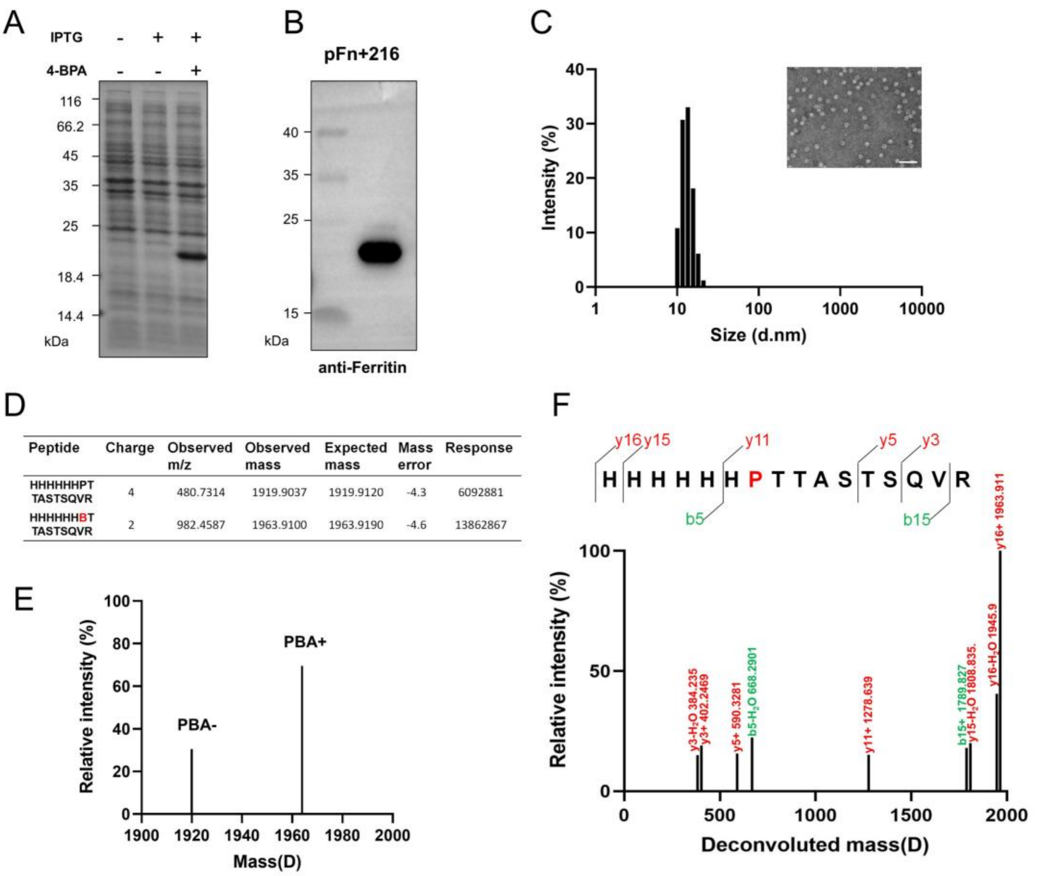

Supplementary Fig. 7 Characterization of PBA incorporation into pFn+ by mass spectrometry. **A.** Comparison of SDS-PAGE for BL21 (DE3) expressing pFn+ with and without the addition of PBA, using pFn+216 as an example. **B.** Immunoblot analysis of PBA incorporation into pFn+ using pMjTyrRS/tRNA and anti-ferritin antibodies. **C.** Determination of the size and morphology of pFn+ by DLS and TEM (bar, 50 nm). **D.** Q-TOF mass spectrometry analysis of PBA-related peptide fragments resulting from trypsin digestion of pFn+. **E.** Analysis of the incorporation efficiency of PBA-related peptide fragments from trypsin-digested pFn+. **F.** Secondary mass spectrometry of PBA-related peptide fragments from trypsin-digested pFn+. The red P indicates PBA.

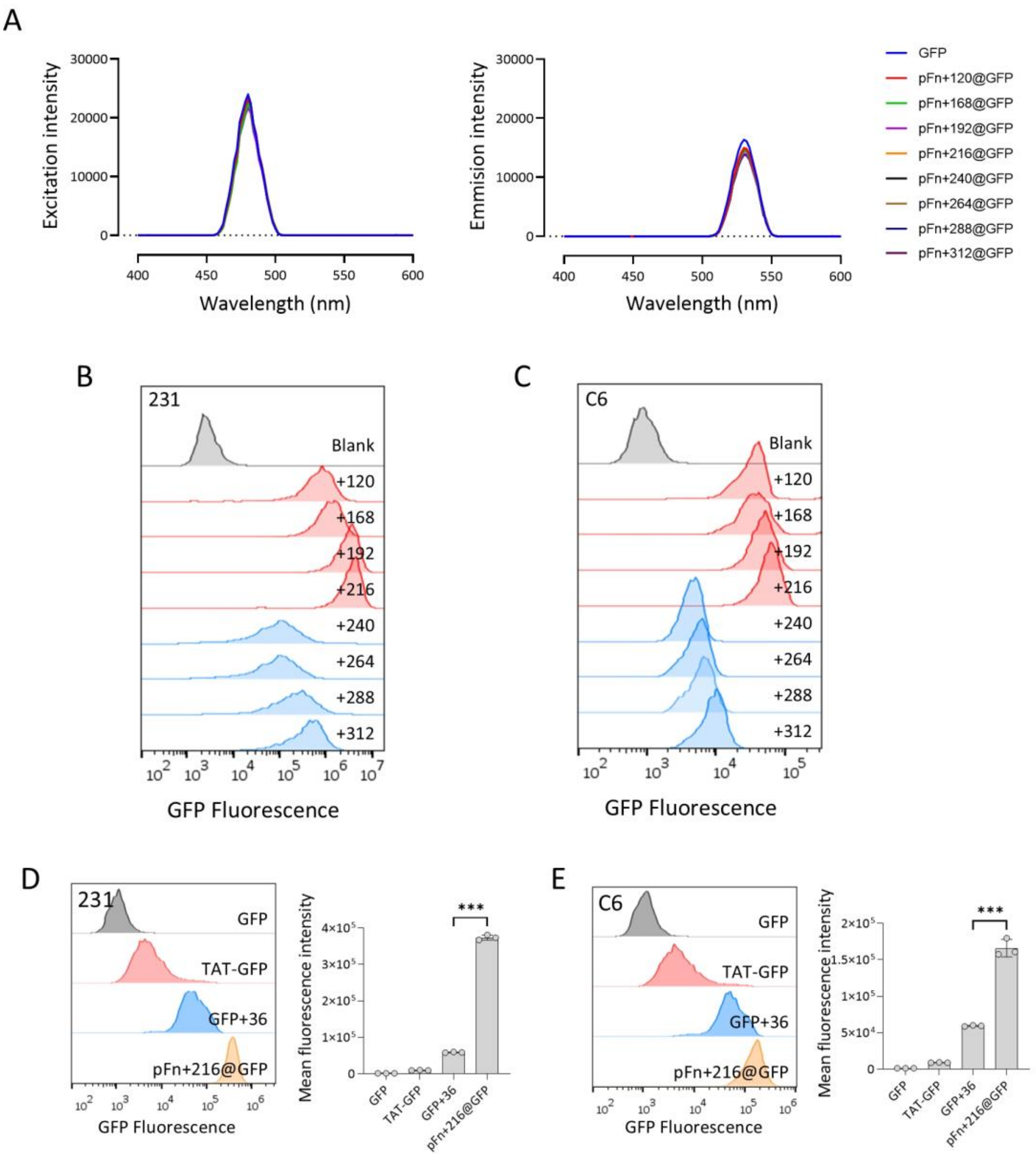

Supplementary Fig. 8 Analysis of cellular uptake efficiency of the pFn+@GFP complex.

A. Representative excitation and emission spectra of the pFn+@GFP (+120~+312) complex, with GFP as a control. **B-C.** Flow cytometric analysis of the uptake efficiency from Fn+120 to Fn+312 in MDA-MB-231 (B) and C6 (C) cells. **D-E.** Flow cytometry analysis of GFP fluorescence intensity in MDA-MB-231 cells (D) and C6 cells (E) after treatment with different formulations (n=3 independent samples). Data are presented as mean ± SEM; statistical significance was determined using one-way ANOVA followed by Sid'k's multiple comparisons test (***P < 0.001).

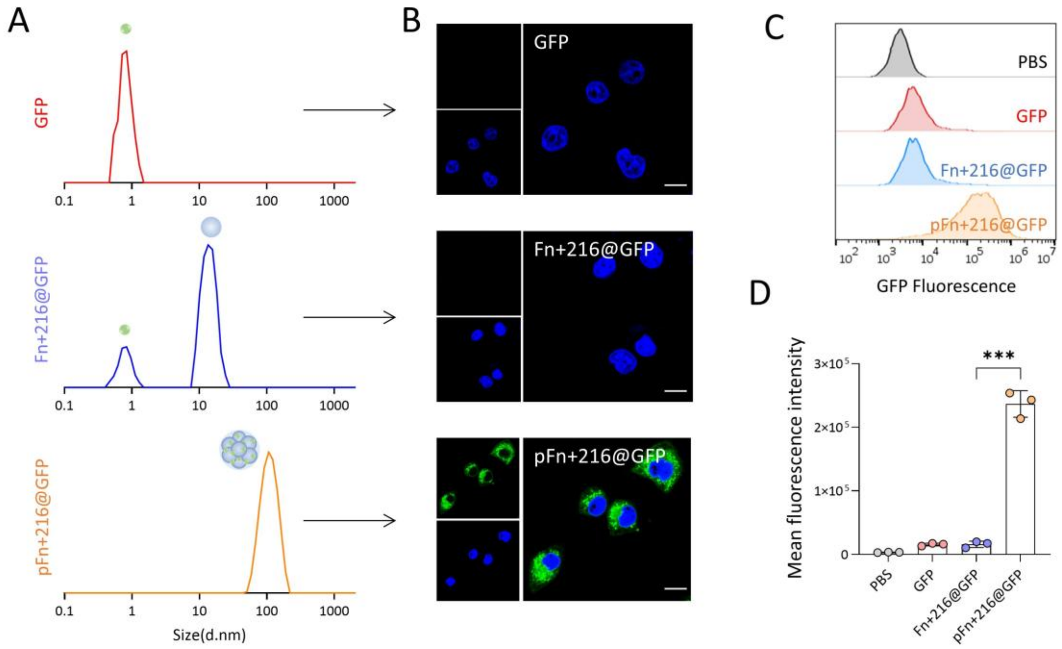

Supplementary Fig. 9 Comparison of PBA with and without modification.

A. DLS analysis of GFP, Fn+216@GFP, and pFn+216@GFP. **B-D.** Confocal Microscopy (B) and Flow Cytometry Analysis (C-D) of GFP Delivery Efficiency by pFn+216 with/without PBA Modification (n=3 independent samples). Scale bar, 10 µm. Data are presented as mean ± SEM; statistical significance was determined using one-way ANOVA followed by Sid'k's multiple comparisons test (***P < 0.001).

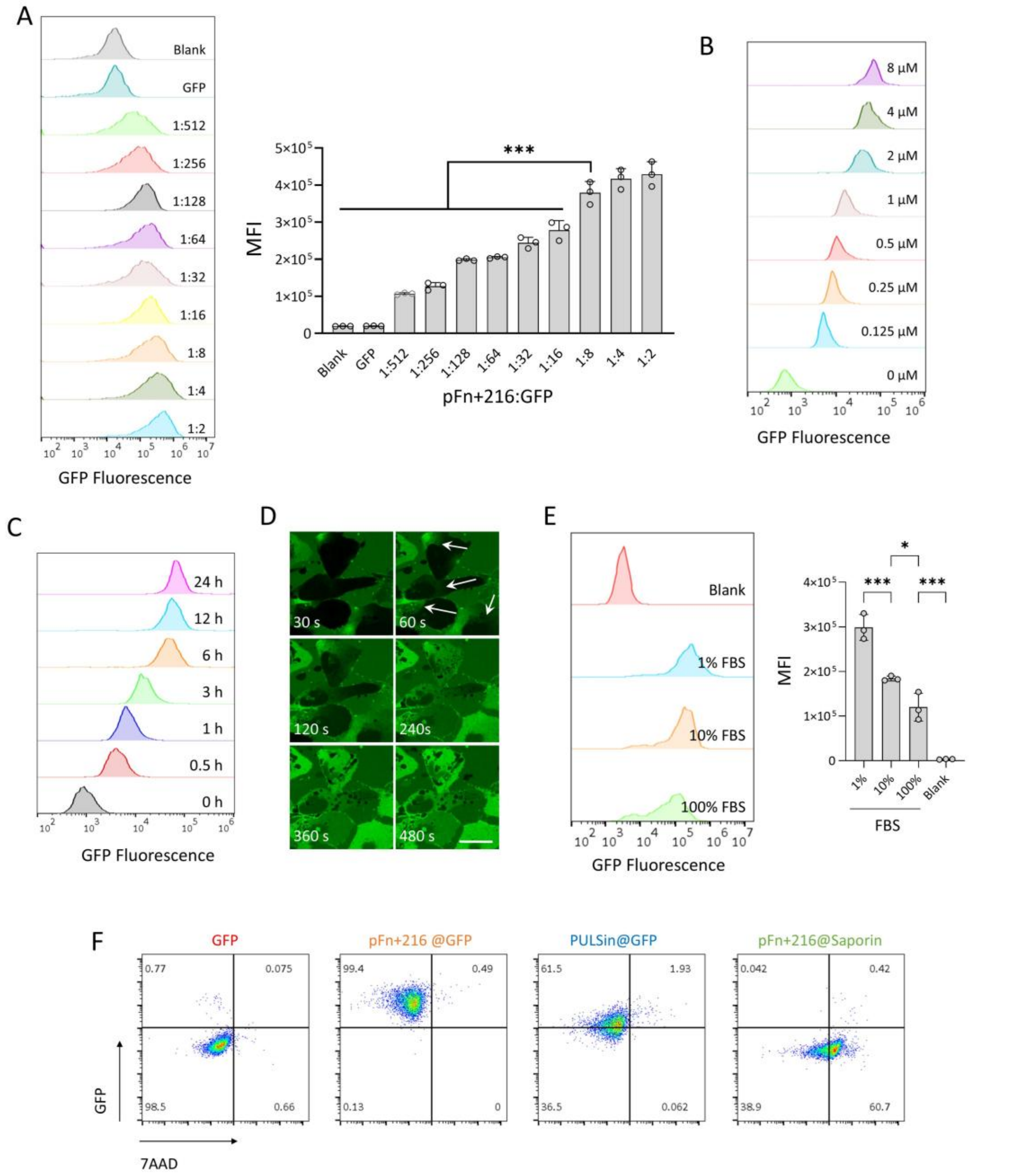

Supplementary Fig. 10 Exploration of the optimal cellular uptake protocol for pFn+216@GFP. **A.** Assessment of cellular uptake efficiency of complexes incubated with varying ratio of pFn+216@GFP for 1 h, as analyzed by flow cytometry. The concentration of each preparation was adjusted to the GFP (1 μM) equivalent concentration (n=3 independent samples). Data are presented as mean ± SEM; statistical significance was determined using one-way ANOVA followed by Dunnett's multiple comparisons test (**P < 0.001). **B-C.** Flow cytometric analysis of MDA-MB-231 cells incubated with different concentrations of pFn+216@GFP for 1 h (B) or incubated for varying durations (C) to evaluate cellular uptake efficiency. **D.** Time-dependent cellular uptake experiment of pFn+216@GFP. Scale bar, 20 μm. **E.** Flow cytometric results following 12 h of treatment with pFn+216@GFP (8 μM GFP) on MDA-MB-231 cells in different serum concentrations (n=3 independent samples). Data are presented as mean ± SEM; statistical significance was determined using one-way ANOVA with Tukey's multiple comparisons test (***P

< 0.001). **F.** After 12 h of treatment, cells were harvested and stained with 7-aminoactinomycin D (7-AAD) for 5 minutes prior to flow cytometric analysis. The Saporin protein was used as a pro-apoptotic control.

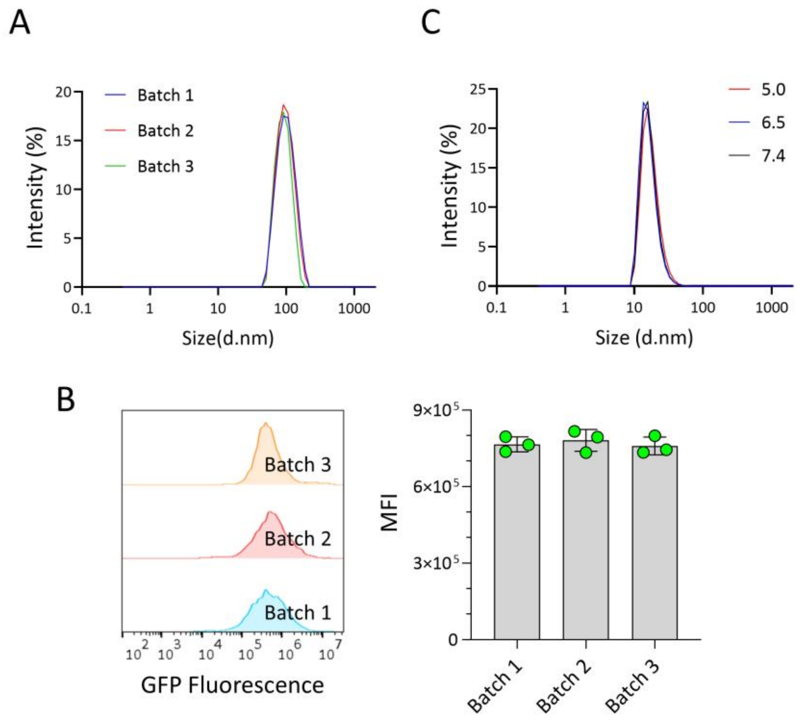

Supplementary Fig. 11 Batch Reproducibility and pH Stability of pFn+216.

A. DLS analysis of pFn+216@GFP complexes from three batches. **B.** Flow cytometry histograms of GFP fluorescence intensity in MDA-MB-231 cells treated with pFn+216@GFP complexes from three batches. **C.** Particle size of pFn+216 at different pH levels was detected by DLS. Data are presented as mean ± SEM (n=3 independent samples).

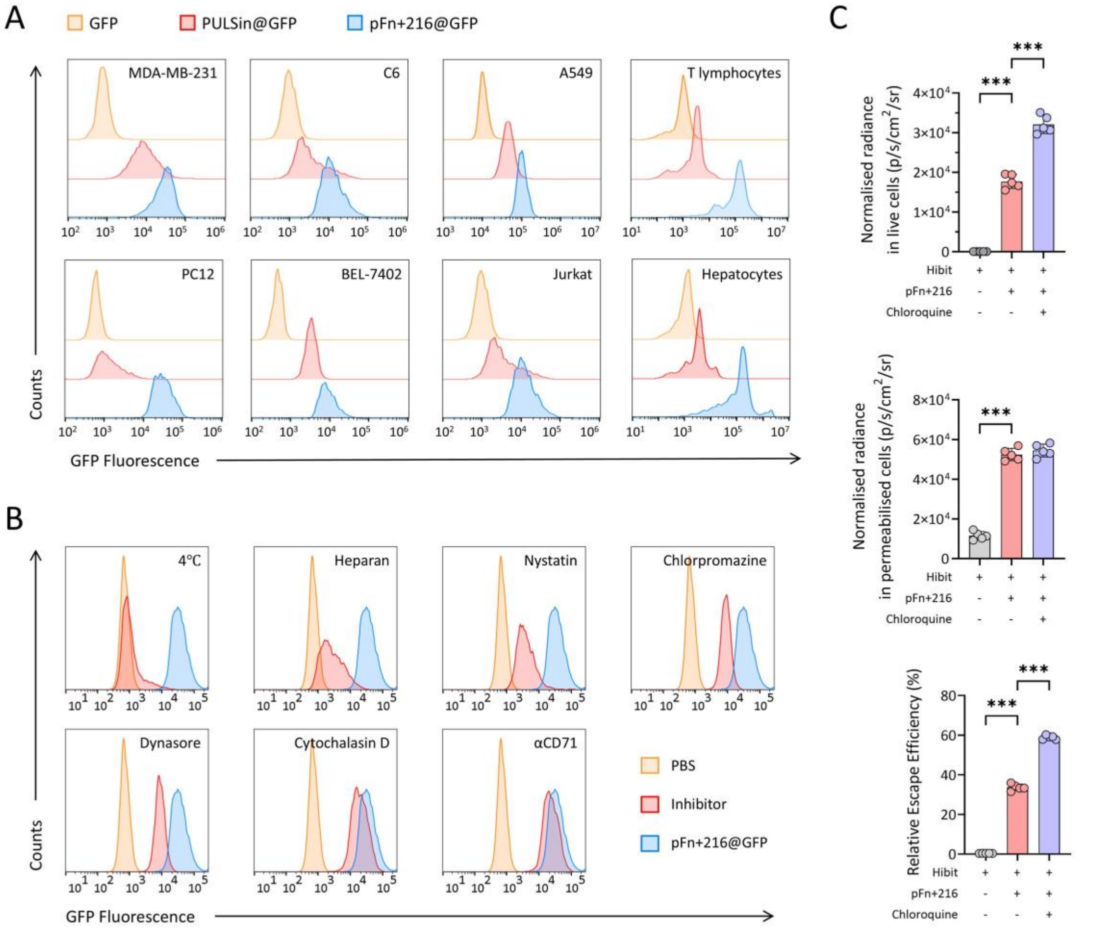

Supplementary Fig. 12 Evaluating the intracellular delivery mediated by pFn+216.

A. Flow cytometry analysis of MDA-MB-231, C6, A549, PC12, BEL-7402, Jurkat, primary T lymphocytes (mouse) and primary hepatocytes (mouse) cells incubated with PULSin@GFP and pFn+216@GFP. **B.** The effect of inhibitors on the internalization of GFP delivered by pFn+216 was analyzed. (**C**) Evaluation of lysosomal escape efficiency of pFn+216@HiBit using the HiBiT-LgBiT assay (n=5 independent samples). Data are presented as mean ± SEM; statistical significance was determined using one-way ANOVA with Tukey's multiple comparisons test (***P < 0.001).

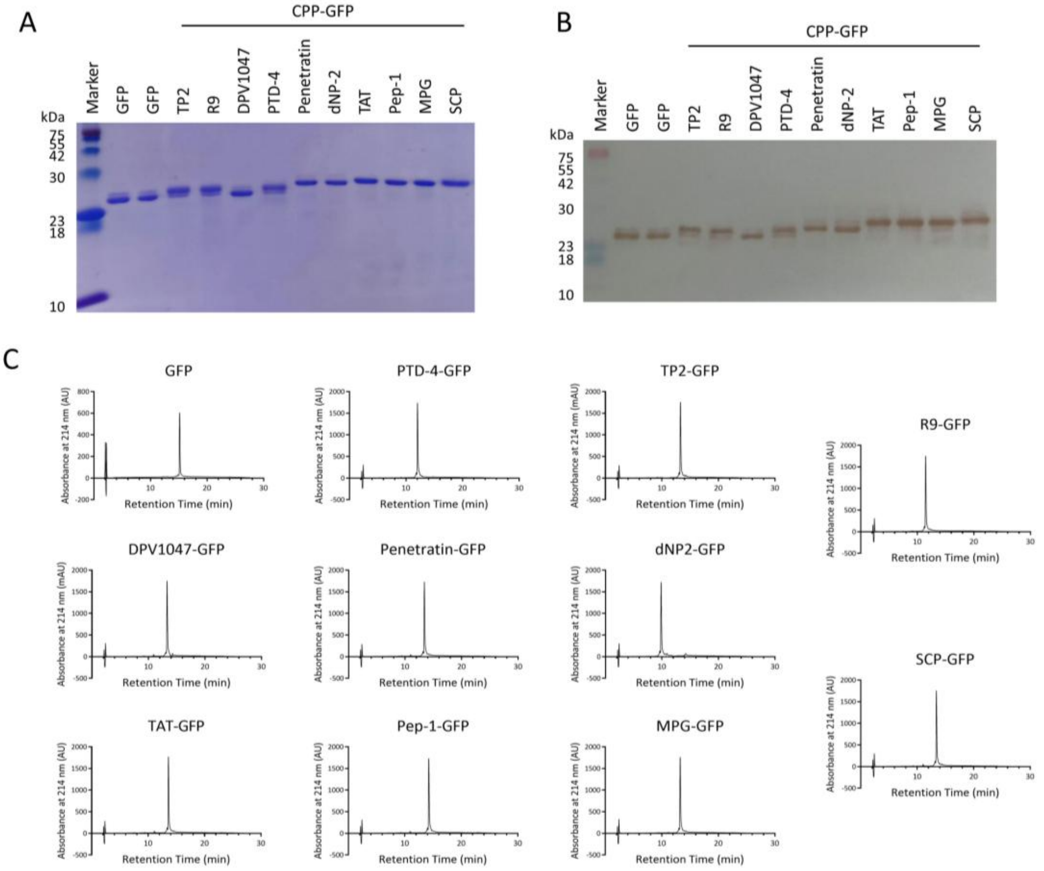

Supplementary Fig. 13 Characterization of recombinant CPP-GFP fusion proteins.

All recombinant proteins, the series of cell-penetrating peptide (TP2, R9, DPV1047, PTD-4, Penetratin, dNP2, TAT, Pep-1, MPG, SCP) fused GFP constructs, were constructed, expressed, and purified in this study. The figure presents a comprehensive characterization to confirm the successful production, purity, and identity of these proteins. **A.** SDS-PAGE gel electrophoresis of a series of CPP-GFP proteins (10 ng/lane). A single, predominant band at the expected molecular size is observed for each CPP-GFP fusion protein and the GFP control, demonstrating successful expression and high purity after purification. **B.** Recombinant proteins from (A) were immunoblotted using a primary antibody against GFP. A specific immunoreactive signal is detected for all samples, confirming that the purified proteins are the intended GFP-containing fusions. **C.** RP-HPLC analysis of a series of CPP-GFP proteins. Each sample displays a single, sharp major peak, indicating a homogeneous preparation. The distinct retention times for different constructs reflect their varying hydrophobicity profiles.

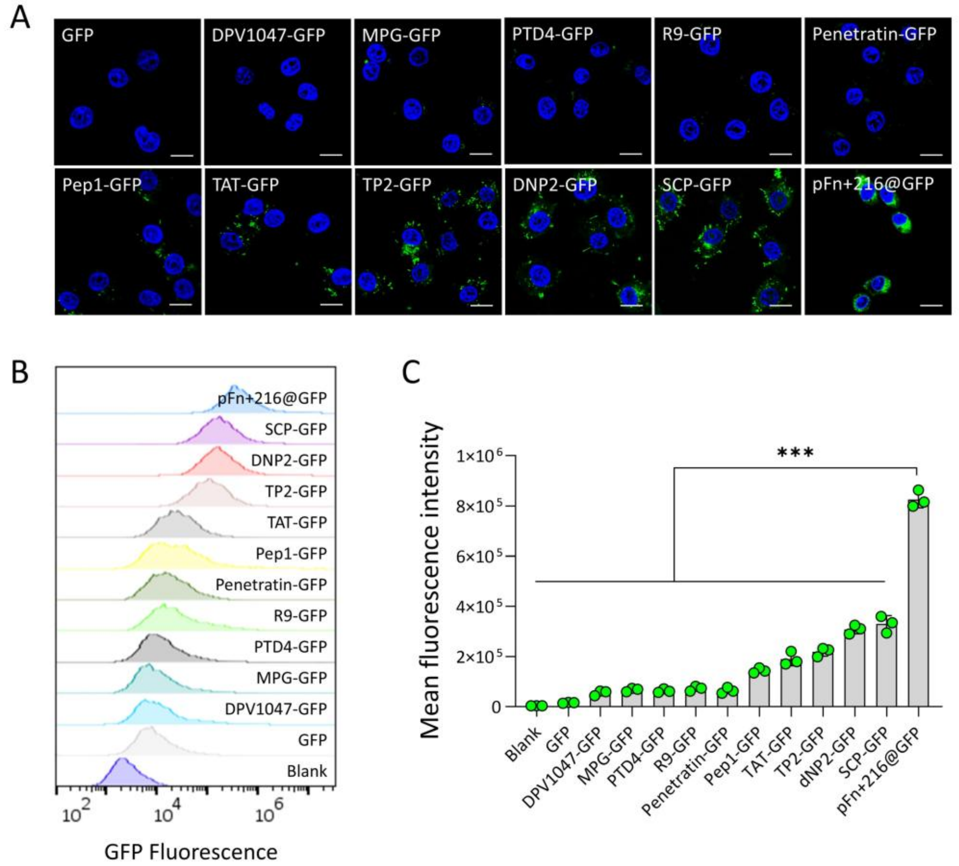

Supplementary Fig. 14 Comparison of delivery efficiency across different cell-penetrating peptides.

A-C. Confocal images (A) and flow cytometry (B-C) analysis of MDA-MB-231 cells treated with a series of cell-penetrating peptides and pFn+216 for 12 h. The doses of GFP were 8 μM in each well (n=3 independent samples). Scale bar, 10 μm. Data are presented as mean ± SEM; statistical significance was determined using one-way ANOVA with Dunnett's multiple comparisons test (***$P < 0.001$).

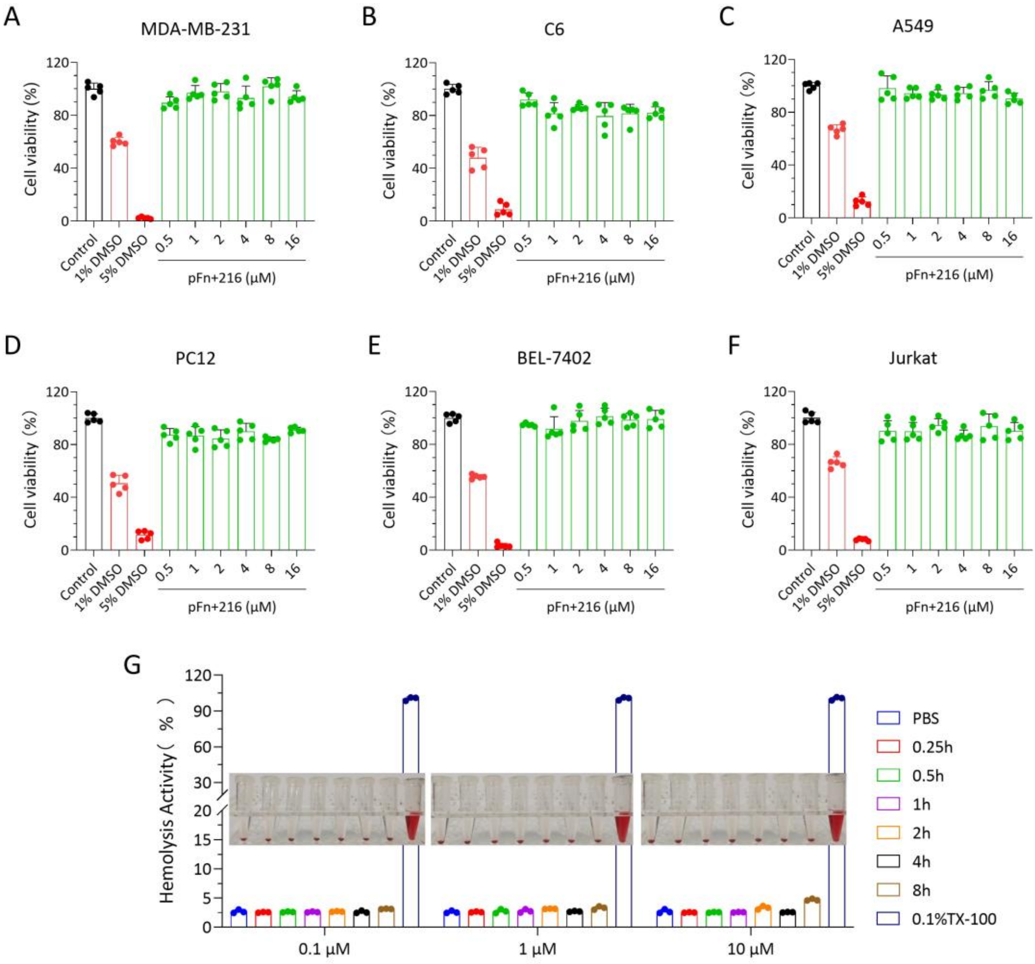

Supplementary Fig. 15 In vitro safety assessment of pFn+216.

A-F. Cytotoxicity of pFn+216. The MTT assay was employed to evaluate the cytotoxic effects of pFn+216 on MDA-MB-231 (A), C6 (B), A549 (C), PC12 (D), BEL-7402 (E), and Jurkat (F) cells after a 24 h incubation with varying concentrations of pFn+216. DMSO was used as a positive control. Cell viability (%) was determined by comparing the treated samples with the control samples (untreated cells, considered as 100% viable) (n=5 independent samples). **G.** Hemolytic toxicity of pFn+216. The hemolytic activity of pFn+216 was assessed at different concentrations and incubation times. PBS and Triton X-100 (0.1%) served as negative and positive control groups, respectively (n=3 independent samples). Data are presented as mean ± SEM.

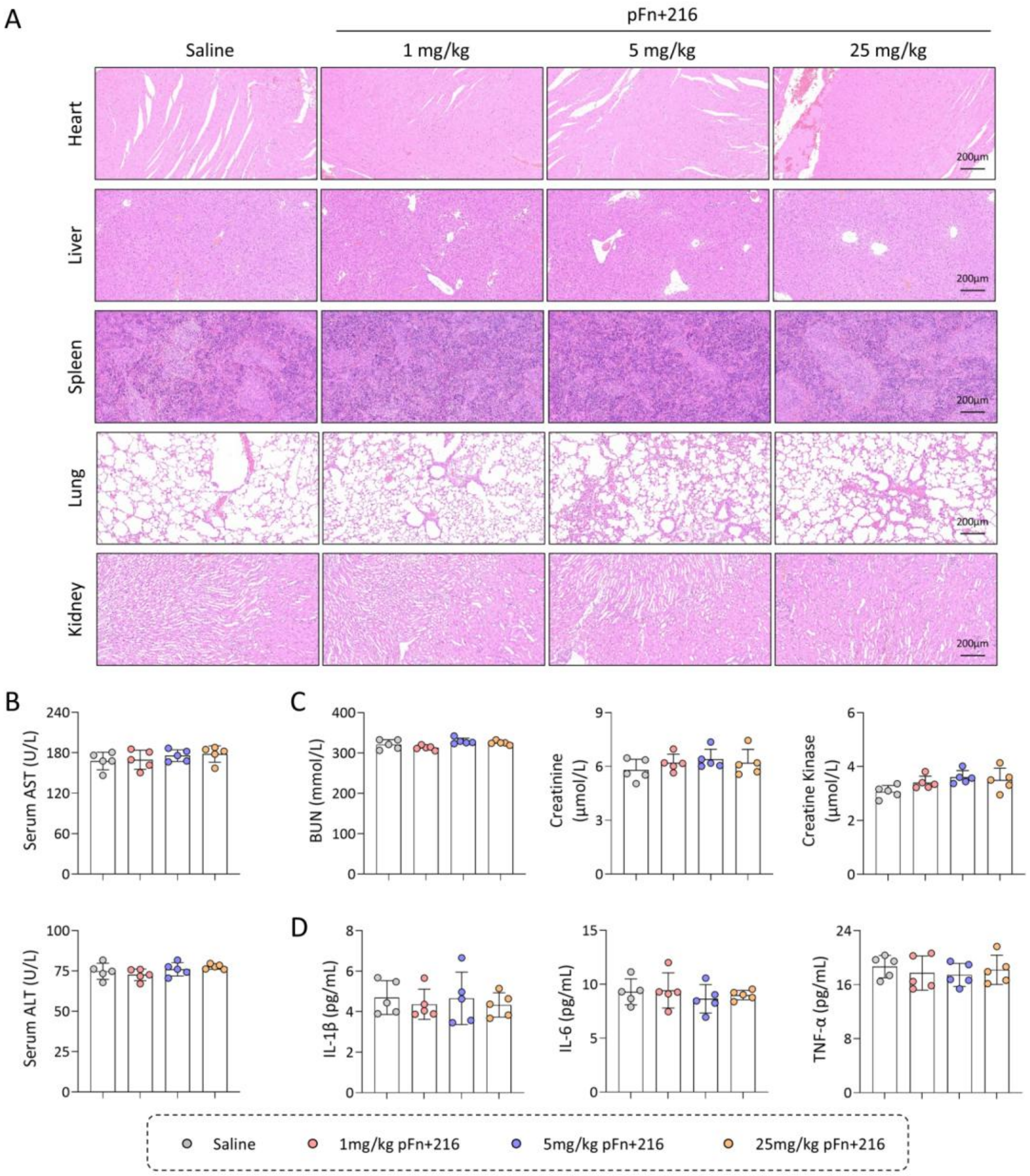

Supplementary Fig. 16 *In vivo* safety assessment following long-term administration of pFn+216.

Mice were administered pFn+216 daily via injection at doses of 1 mg/kg, 5 mg/kg, and 25 mg/kg for 14 days. **A.** Representative H&E-stained sections of major organs from mice. **B.** Evaluation of liver function: Alanine aminotransferase (ALT) and Aspartate aminotransferase (AST) levels were measured. **C.** Evaluation of kidney function: Blood urea nitrogen (BUN), Creatine Kinase (CK), and Creatinine levels were determined. **D.** Serum concentrations of IL-6, IL-1β, and TNF-α were quantified by ELISA. Data are presented as mean ± SEM (n=5 independent samples).

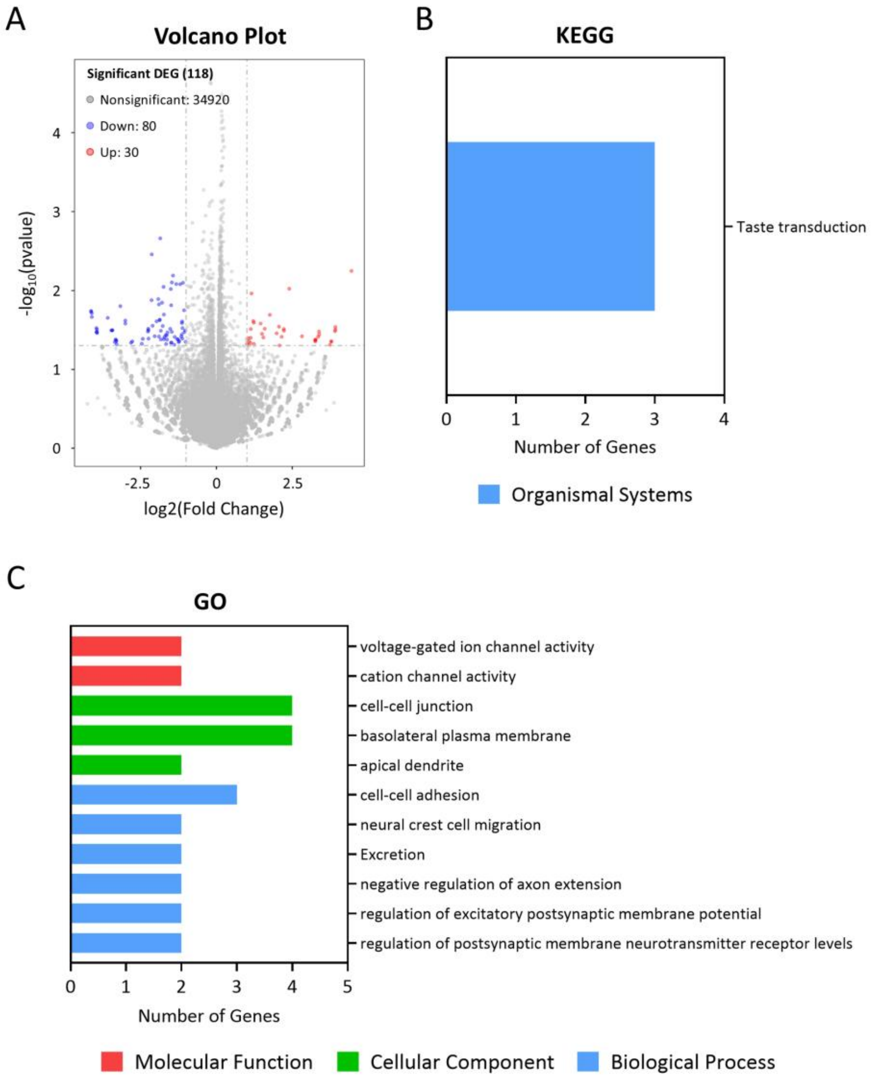

Supplementary Fig. 17 Transcriptome analysis after pFn+216 administration.

A. Volcano plot showing differentially expressed genes in MDA-MB-231 cells treated with pFn+216. Differential gene expression was filtered using thresholds of $p < 0.05$ and $|log_2FC| > 1$.

B-C. Functional enrichment analysis of differentially expressed genes: (B) KEGG pathway analysis ($p < 0.05$); (C) Gene Ontology (GO) terms ($p < 0.05$).

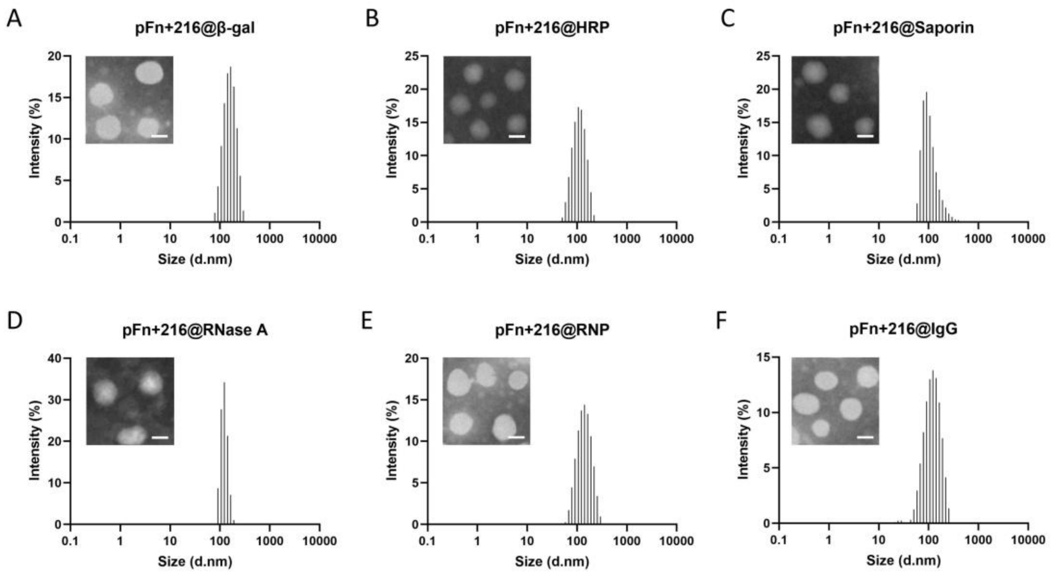

Supplementary Fig. 18 Characterization of different pFn+216/Cargos complexes.

The size of the complexes composed of pFn+216@β-gal, pFn+216@HRP, pFn+216@Saporin, pFn+216@RNase A, pFn+216@RNP, and pFn+216@IgG (molar ratio of pFn+216 and Cargo is 1:8) was determined. The size and morphology of the nanocomposite were assessed using DLS and TEM. Scale bar, 100 nm.

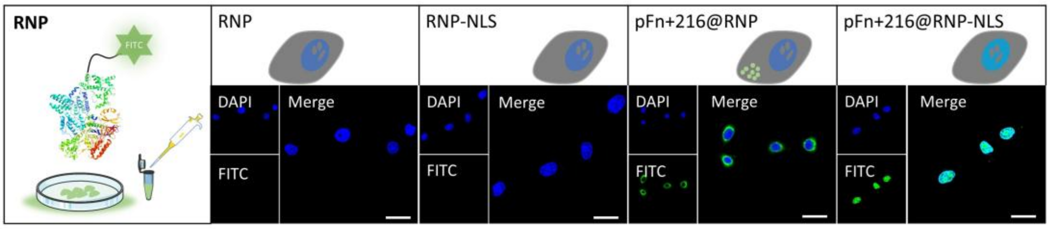

Supplementary Fig. 19 Cytoplasmic delivery of the Cas9/sgRNA complex RNP mediated by pFn+216.

Cytoplasm-released RNP can enter the nucleus via nuclear localization sequences to exert gene editing effects. Confocal images of FITC-labeled RNP complex (1 μM RNP) delivered by pFn+216. After 24 h of incubation, cells were fixed, permeabilized, and stained with DAPI (blue) to visualize nuclei. Scale bar, 20 μm.

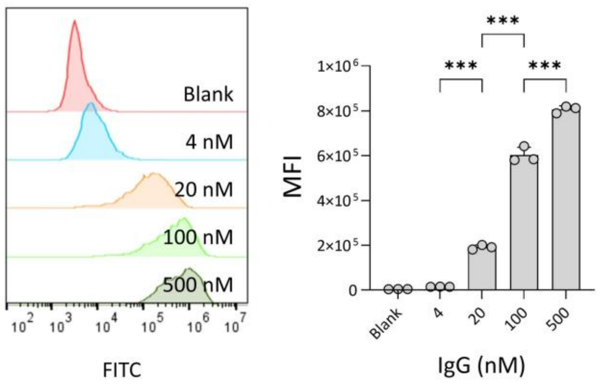

Supplementary Fig. 20 Flow cytometric analysis of intracellular delivery of antibodies via pFn+216.

Flow cytometric analysis of complexes formed between different concentrations of FITC-labeled IgG (Non-specific IgG) and pFn+216 after incubation with live MDA-MB-231 cells for 24 h, along with changes in MFI(n=3 independent samples). Data are presented as mean ± SEM; statistical significance was determined using one-way ANOVA with Tukey's multiple comparisons test (***P < 0.001).

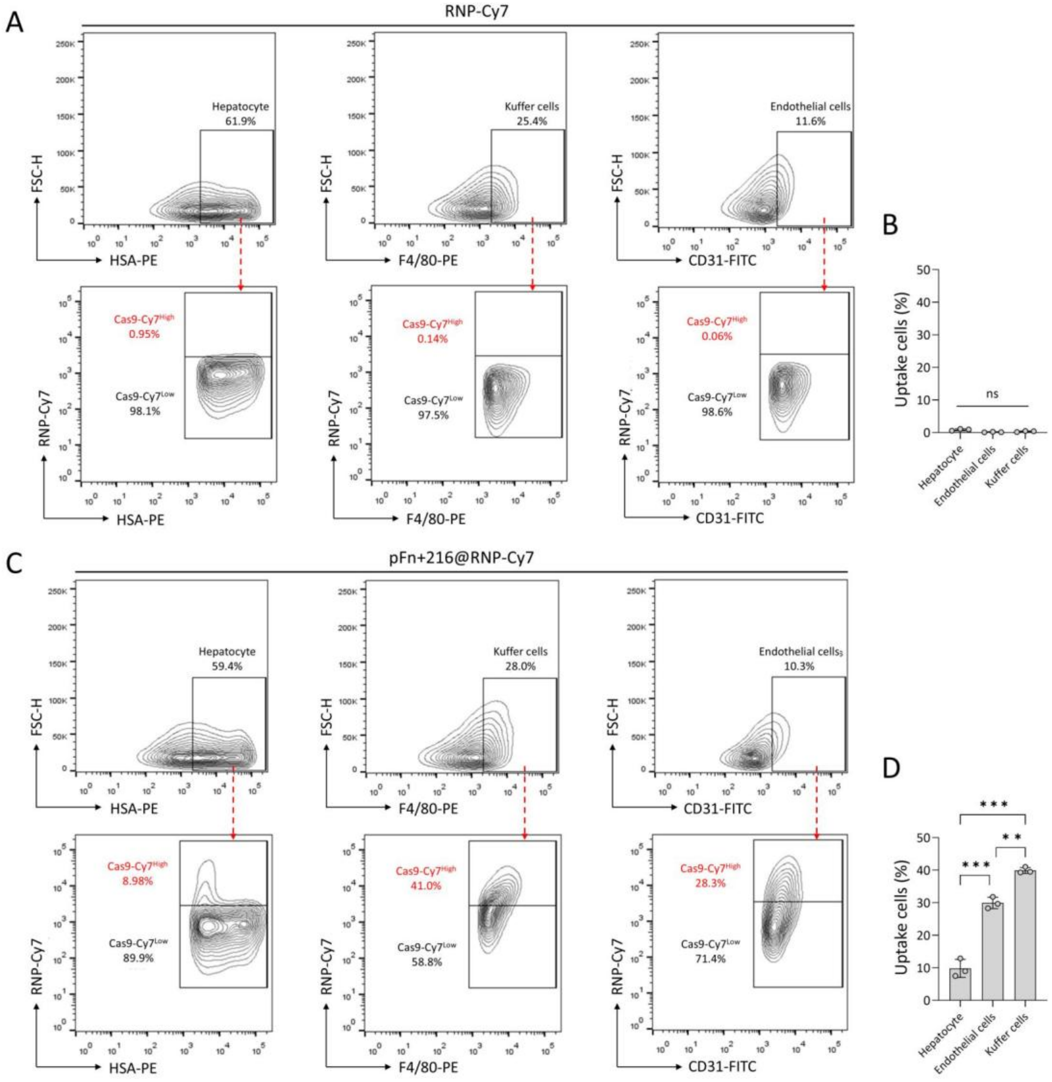

Supplementary Fig. 21 *In vivo* uptake of pFn+216@RNP by liver cell populations.

Primary mouse liver cells were isolated 24 hours after a single intravenous injection of pFn+216@RNP nanoparticles (Cy7-labeled Cas9). Cells were co-stained with fluorescent antibodies against specific cell markers: HSA (for hepatocytes), F4/80 (for Kupffer cells), and CD31 (for endothelial cells), and analyzed by flow cytometry. **A&C.** (Top panels) Gating plots for live cells, with target populations (Hepatocytes, Kupffer cells, Endothelial cells) outlined and labeled with their percentage of total live cells. (Bottom panels) Corresponding histograms showing Cy7 fluorescence intensity (indicating RNP uptake) within the respective gated cell populations. **B&D.** The percentages of cells with high Cy7 signal ($Cy7^{High}$) are indicated in red. Quantitative analysis (n=3 independent animals) of the percentage of $Cy7^{High}$ cells within each cell population. Kupffer cells exhibited the highest uptake efficiency (n=3 independent animals). Data are mean ± SEM; statistical significance was determined using one-way ANOVA with Tukey's multiple comparisons test (***P < 0.001).

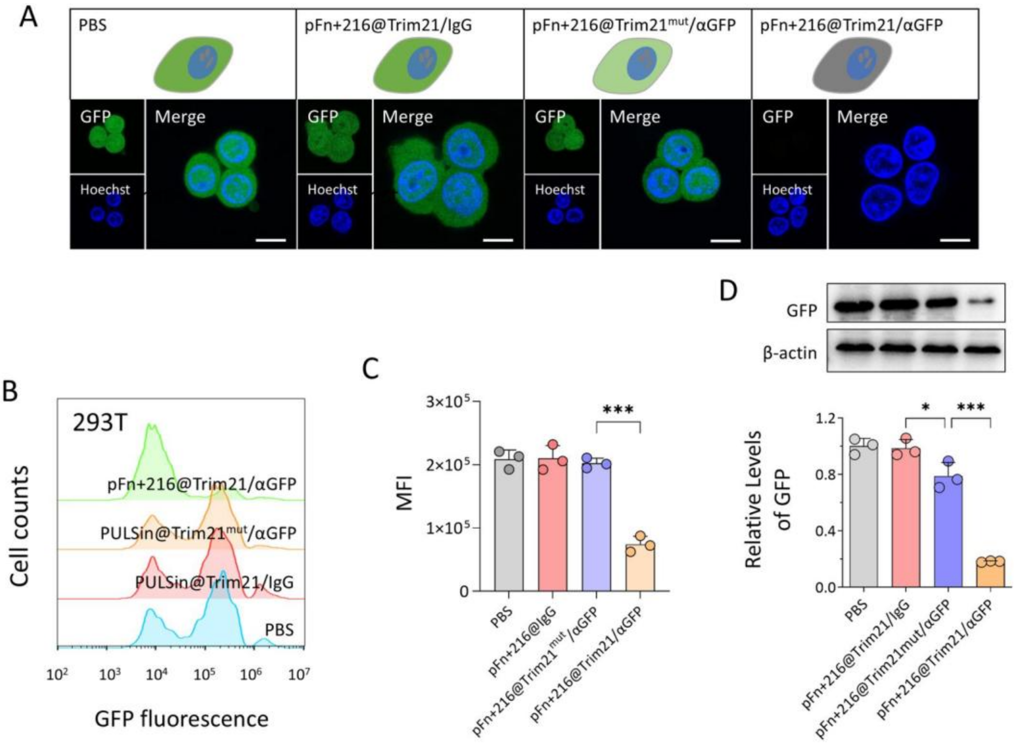

Supplementary Fig. 22 Control experiment for Trim-Away mechanism.

A. Representative live-cell confocal microscopy image of a monolayer of HEK-293T-GFP cells incubated with pFn+216@Trim21/αGFP for 24 h. Nuclei were stained with Hoechst 33342 (blue). Scale bar: 10 μm. **B-C.** Flow cytometric analysis showing the change in average fluorescence intensity of HEK-293T-GFP cells treated with pFn+216@Trim21/αGFP (n=3 independent samples). Data are presented as mean ± SEM; statistical significance was determined using one-way ANOVA with Sid'k's multiple comparisons test (***P<0.001). **D.** Immunoblot analysis showing targeted degradation of GFP protein in HEK-293T-GFP cells mediated by pFn+216@Trim21/αGFP. IgG served as an isotype control antibody; Trim21^{mut} was included as a catalytically inactive RING-domain mutant of Trim21 (C16A) (n=3 independent samples). Data are presented as mean ± SEM; statistical significance was determined using one-way ANOVA with Sid'k's multiple comparisons test (*P<0.05, ***P<0.001).

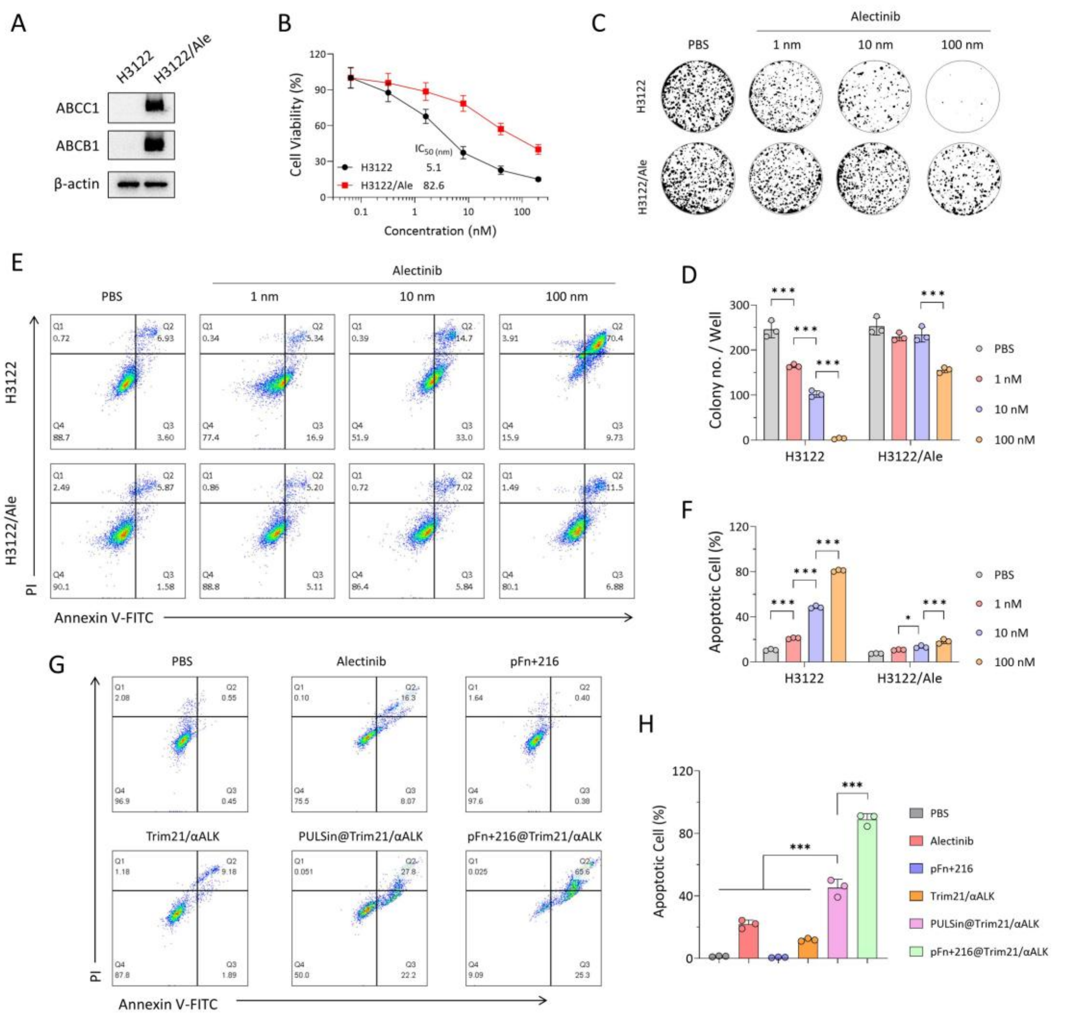

Supplementary Fig. 23 Establishment of multidrug-resistant tumor cells.

The establishment method of H3122/Ale cell line, as detailed in our previous work. The parental cells were seeded into a 12-well plate and grown to approximately 90% confluence, and the first dose of the chemotherapeutic drugs was then applied at a concentration of 1 nM. The medium was changed after 4 h of drug exposure, and the cells that were alive 24 h after the removal of the drug were considered resistant cells. The chemotherapeutic drug-resistant cells were collected and cultured until they resumed proliferating and established stable clones. The drug-resistant cells were further inoculated into a 12-well plate, and the above process was performed until the cells no longer exhibited significant apoptosis during exposure to increasing concentrations of the drugs (the concentration of the drug was increased by 50 nM). **A.** Western blot analysis showing upregulation of the efflux transporters ABCB1 and ABCC1 in H3122/Ale cells compared to parental H3122 cells. **B.** H3122/Ale cells were treated with the indicated concentrations of Alectinib for 48 h. Cell viability was measured by CCK8 assay (n=5 independent samples). **C-D.** Representative images **(C)** and quantification **(D)** of clonogenic survival assays for parental H3122 cells treated with Alectinib (1, 10, 100 nM), showing potent, dose-dependent inhibition (n=3 independent samples). Data are presented as mean ± SEM; statistical significance was determined using one-way ANOVA with

Tukey's multiple comparisons test (***P < 0.001). **E-F.** Flow cytometry analysis of apoptosis assessed by Annexin V-FITC and PI staining (E). Quantitative analysis of total apoptosis, calculated as the sum of early and late apoptotic cells (Q2 + Q3) from the flow cytometry data (F) (n=3 independent samples). Data are presented as mean ± SEM; statistical significance was determined using one-way ANOVA with Tukey's multiple comparisons test (*P < 0.05, ***P < 0.001). **G-H.** Flow cytometry analysis of apoptosis assessed by Annexin V-FITC and PI staining. H3122/Ale cells were treated as indicated for 48 h (G). Quantitative analysis of total apoptosis, calculated as the sum of early and late apoptotic cells (Q2 + Q3) from the flow cytometry data (H). Data are presented as mean ± SEM (n=3 independent samples). Data are presented as mean ± SEM; statistical significance was determined using one-way ANOVA with Dunnett's multiple comparisons test (***P<0.001).

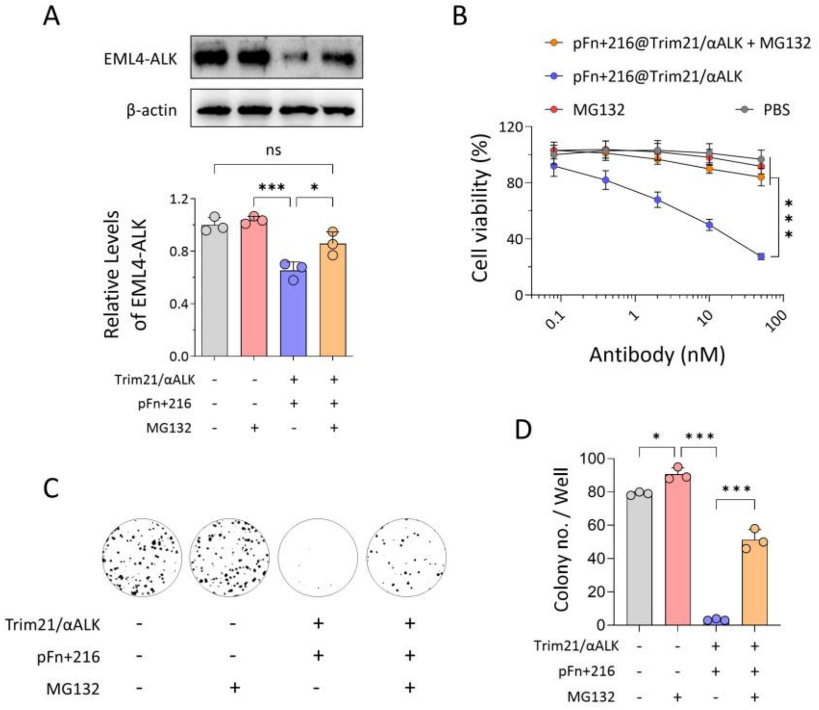

Supplementary Fig. 24 MG132 blocks the anti-tumor effect of pFn+216@Trim21/αALK.

A. Western blot analysis showing the expression levels of EML4-ALK and β-actin in H3122/Ale cells. Co-treatment with the proteasome inhibitor MG132 prevented the degradation of EML4-ALK induced by pFn+216@Trim21/αALK. Bar graphs provide quantitative densitometry analysis of the protein bands (n=3 independent samples). Data are presented as mean ± SEM; statistical significance was determined using one-way ANOVA with Tukey's multiple comparisons test (***P < 0.001). **B.** Cell viability assessed by CCK-8 assay. The cytotoxic effect of pFn+216@Trim21/αALK was significantly rescued by MG132 treatment, restoring cell viability over time (n=3 independent samples). Data are presented as mean ± SEM; statistical significance was determined using two-way ANOVA with Dunnett's multiple comparisons test (***P < 0.001). **C-D**. Representative images (C) and quantification (D) of clonogenic survival assays. MG132 co-

treatment almost completely abrogated the inhibition of colony formation caused by pFn+216@Trim21/αALK (n=3 independent samples). Data are presented as mean ± SEM; statistical significance was determined using one-way ANOVA with Tukey's multiple comparisons test (*P<0.05, ***P<0.001).

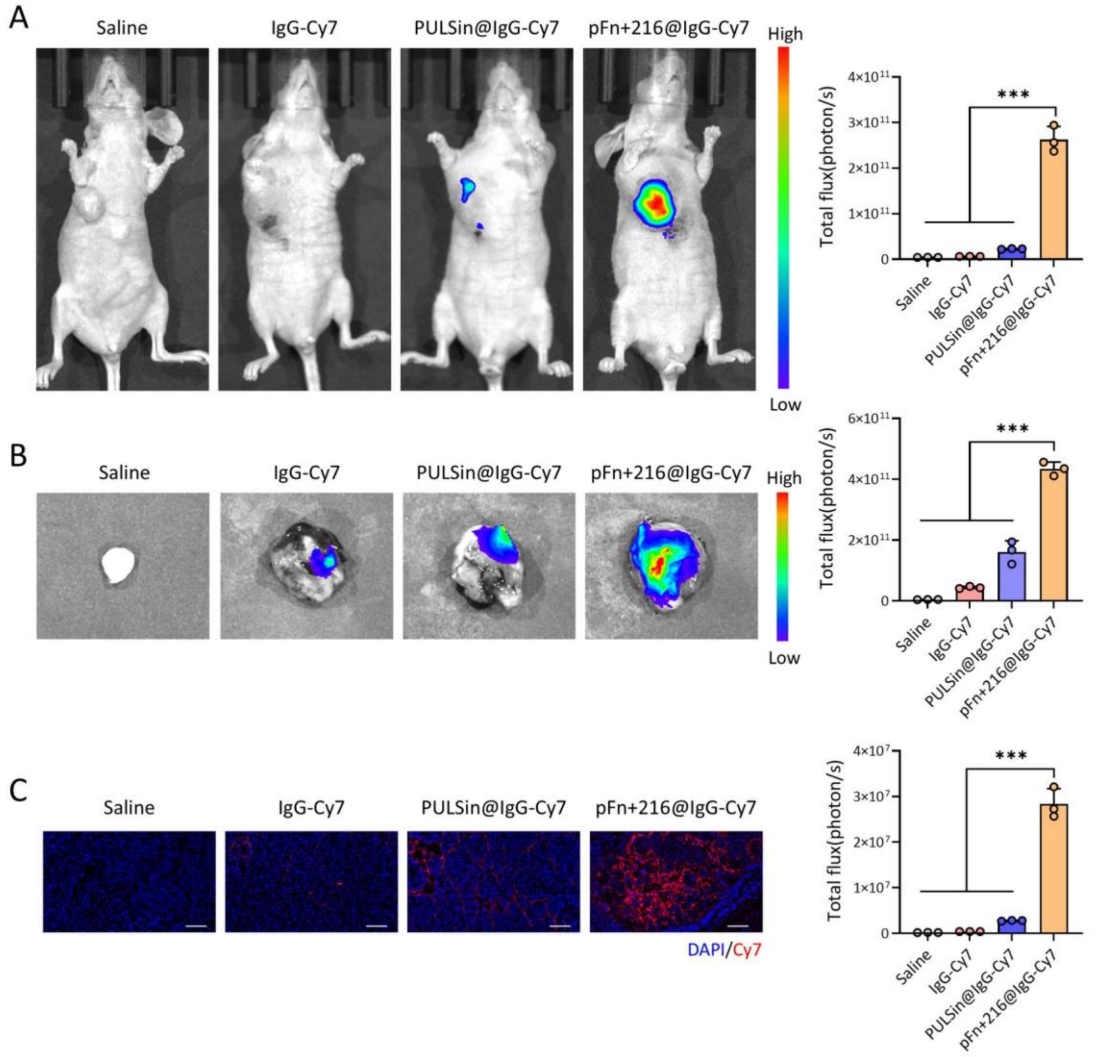

Supplementary Fig. 25 *In vivo* biodistribution and tumor delivery of pFn+216@IgG-Cy7.
A. *In vivo* fluorescence imaging of tumor-bearing mice at 12 hours post-intratumoral injection of the indicated formulations: Saline, IgG-Cy7 alone, PULSin@IgG-Cy7, and pFn+216@IgG-Cy7. The right panel shows the quantitative analysis of the total radiant efficiency within the tumor region, demonstrating significantly enhanced accumulation of the pFn+216-based formulation (n=3 independent animals). Data are presented as mean ± SEM; statistical significance was determined using one-way ANOVA with Dunnett's multiple comparisons test (***P<0.001). **B.** *Ex vivo* fluorescence imaging of excised tumors corresponding to the treatment groups shown in (A). The pFn+216@IgG-Cy7 group exhibits the most intense and focused fluorescence signal within the tumor tissue (n=3 independent animals). Data are presented as mean ± SEM; statistical significance

was determined using one-way ANOVA with Dunnett's multiple comparisons test (***P<0.001). **C.** Representative fluorescence micrographs of tumor tissue sections. Cell nuclei are stained with DAPI (blue). The delivered IgG-Cy7 (red) shows pronounced and widespread intracellular distribution only in the pFn+216@IgG-Cy7 treatment group (n=3 independent animals). Scale bar: 100 μm. Data are presented as mean ± SEM; statistical significance was determined using one-way ANOVA with Dunnett's multiple comparisons test (***P<0.001).

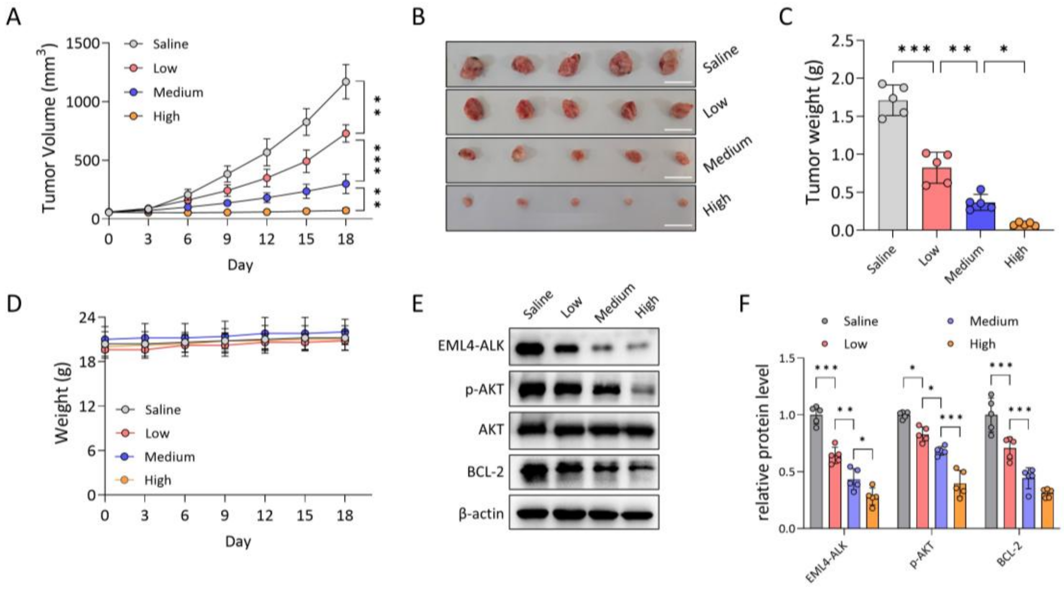

Supplementary Fig. 26 Dose-dependent antitumor efficacy of pFn+216@Trim21/αALK in H3122/Ale xenograft models.

Using the H3122/Ale subcutaneous tumor model, mice received intratumoral injections of pFn+216@Trim21/αALK complexes or saline every 3 days. Doses are expressed as the equivalent amount of αALK IgG per mouse: high (1 μg), medium (0.5 μg), and low (0.25 μg). **A.** Tumor growth curves over 18 days, showing a pronounced dose-dependent inhibition (n=5 independent animals). Data are presented as mean ± SEM; statistical significance was determined using two-way ANOVA with Tukey's multiple comparisons test (**P < 0.01, ***P < 0.001). **B.** Representative photographs of excised tumors from each treatment group at the study endpoint. Scale bar: 2 cm. **C.** Quantification of tumor weights at endpoint, consistent with the volume measurements (n=5 independent animals). Data are presented as mean ± SEM; statistical significance was determined using one-way ANOVA with Tukey's multiple comparisons test (*P < 0.05, **P < 0.01, ***P < 0.001). **D.** Mouse body weight changes over the treatment period, indicating no significant systemic toxicity associated with the treatment. **E-F.** Western blot analysis of p-AKT, total AKT, BCL-2, and EML4-ALK under different treatments (E), with protein expression levels determined by densitometry (F) (n=5 independent animals). Data are presented as mean ± SEM; statistical significance was determined using one-way ANOVA with Sid'k's multiple comparisons test (*P < 0.05, **P < 0.01, ***P < 0.001).

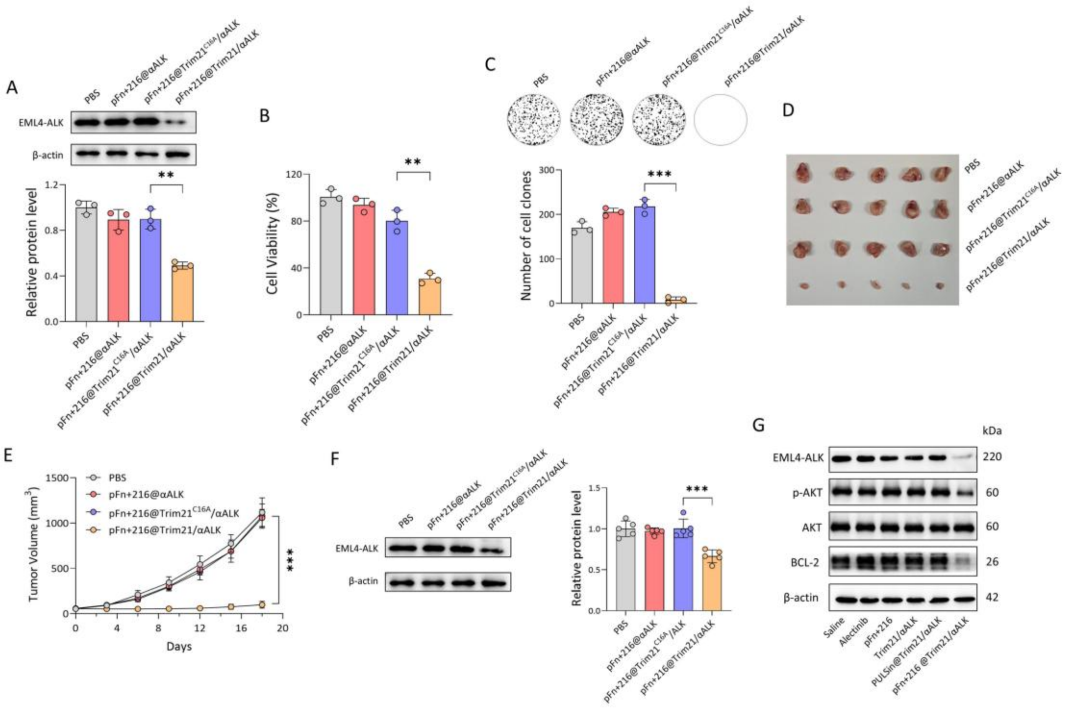

Supplementary Fig. 27 Trim21 catalytic activity is required for EML4-ALK degradation.

A. Western blot analysis of EML4-ALK protein levels in H3122/Ale cells treated with PBS, pFn+216@αALK, pFn+216@Trim21^{C16A}/αALK, or pFn+216@Trim21/αALK. Quantification of relative protein levels normalized to β-actin is shown below (n=3 independent samples). Data are presented as mean ± SEM; statistical significance was determined using one-way ANOVA with Sid'k's multiple comparisons test (**$P < 0.01$). **B.** Cell viability assay (CCK-8) of H3122/Ale cells treated with the indicated conditions (n=3 independent samples). Data are presented as mean ± SEM; statistical significance was determined using one-way ANOVA with Sid'k's multiple comparisons test (**$P < 0.01$). **C.** Representative images and quantification of clonogenic survival assay. H3122/Ale cells were treated as indicated, seeded at low density, and cultured for 14 days. Colonies were stained with crystal violet and counted (n=3 independent samples). Data are presented as mean ± SEM; statistical significance was determined using one-way ANOVA with Sid'k's multiple comparisons test (***$P < 0.001$). **D.** Representative images of tumor tissues harvested from H3122/Ale xenograft-bearing mice after 18 days of treatment with the indicated conditions. **E.** Tumor growth curves showing tumor volume over time. Mice bearing H3122/Ale xenografts were treated intravenously with PBS, pFn+216@αALK, pFn+216@Trim21^{C16A}/αALK, or pFn+216@Trim21/αALK (n=5 independent animals). Data are presented as mean ± SEM; statistical significance was determined using two-way ANOVA with Dunnett's multiple comparisons test (***$P < 0.001$). **F.** Western blot analysis of EML4-ALK protein levels in tumor tissues from xenograft experiments. Quantification of relative protein levels normalized to β-actin is shown below (n=5 independent animals). Data are presented as mean ± SEM; statistical significance was determined using two-way ANOVA with Sid'k's multiple comparisons test (***$P < 0.001$). **(G)**

Western blot analysis of p-AKT, total AKT, BCL-2, and EML4-ALK under different treatments.

Supplementary Table 1. Amino acid sequence of engineering proteins.

Name	Sequence	Ref
**TAT-GFP**	GRKKRRQRRR-GGGGS- [GFP Sequence]	[1]
**R9-GFP**	RRRRRRRRR-GGGGS- [GFP Sequence]	[1]
**MPG-GFP**	GALFLGFLGAAGSTMGAWSQPKKKRKV-GGGGS- [GFP Sequence]	[2]
**Penetratin-GFP**	RQIKIWFQNRRMKWKK-GGGGS- [GFP Sequence]	[3]
**dNP2-GFP**	KIKKVKKKGRKKIKKVKKKGRK-GGGGS- [GFP Sequence]	[4]
**TP2-GFP**	PLIYLRLLRGQF-GGGGS- [GFP Sequence]	[5]
**PTD4-GFP**	YARAAARQARA-GGGGS- [GFP Sequence]	[6]
**DPV1047-GFP**	VKRGLKLRHVRPRVTRMDV-GGGGS- [GFP Sequence]	[7]
**Pep1-GFP**	KETWWETWWTEWSQPKKKRKV-GGGGS- [GFP Sequence]	[8]
**SCP-GFP**	GSKAKPKKTKGSKAKPKKTKGTKAKPKSKKGTKKSKKAKP-GGGGS- [GFP Sequence]	[9]

Note: Amino acid sequence of CPP[1-9] and Flexible Linker.

Supplementary References:

[1] Zorko M, et al. Cell-penetrating peptides in protein mimicry and cancer therapeutics. Adv Drug Deliv Rev, 2022, 180: 114-144.

[2] Zhao Y, et al. Engineered Histidine-Rich Peptides Enhance Endosomal Escape for Antibody-Targeted Intracellular Delivery of Functional Proteins. Angew Chem Int Ed Engl, 2023, 62(38): e202304692.

[3] Kauffman W B, et al. Mechanism Matters: A Taxonomy of Cell Penetrating Peptides. Trends Biochem Sci, 2015, 40(12): 749-764.

[4] Lim S, et al. dNP2 is a blood-brain barrier-permeable peptide enabling ctCTLA-4 protein delivery to ameliorate experimental autoimmune encephalomyelitis. Nat Commun, 2015, 6: 8244-8256.

[5] Langel Ü. Cell-Penetrating Peptides and Transportan. Pharmaceutics, 2021, 13(7): 987-1017.

[6] Naskalska A, et al. Artificial Protein Cage Delivers Active Protein Cargos to the Cell Interior. Biomacromolecules, 2021, 22(10): 4146-4154.

[7] Samec T, Boulos J, Gilmore S, et al. Peptide-based delivery of therapeutics in cancer treatment. Mater Today Bio, 2022, 14: 100-116.

[8] Yan L H, et al. Enhanced Transdermal Absorption of Hyaluronic Acid via Fusion with Pep-1 and a Hyaluronic Acid Binding Peptide. Macromol Biosci, 2023, 23(3): e2200173.

[9] Wang Q, et al. Cytosolic Protein Delivery for Intracellular Antigen Targeting Using Supercharged Polypeptide Delivery Platform. Nano Lett. 2021 Jul 28;21(14):6022-6030.
