# Supplementary material for: Supercharged ferritin nanocages enable universal cytosolic protein delivery
Source: Nat Commun. 2026 Jun 9;17:7319. doi: 10.1038/s41467-026-74247-x (PMC13402336; doi:10.1038/s41467-026-74247-x)
Supplement: Supplementary file 2 — Description of Additional Supplementary Files [file 41467_2026_74247_MOESM2_ESM.pdf]

Description of Additional Supplementary Files

Supplementary Movie 1: Intracellular delivery of GFP mediated by supercharged ferritin nanocages in MDA-MB-231 cells. Images were acquired every 1 sec for 8 min by confocal microscopy.
