# Supplementary material for: Supercharged ferritin nanocages enable universal cytosolic protein delivery
Source: Nat Commun. 2026 Jun 9;17:7319. doi: 10.1038/s41467-026-74247-x (PMC13402336; doi:10.1038/s41467-026-74247-x)
Supplement: Supplementary file 5 — Transparent Peer Review File [file 41467_2026_74247_MOESM5_ESM.pdf]

nature portfolio

Peer Review File

Supercharged ferritin nanocages enable universal cytosolic protein delivery

Corresponding Author: Professor Jun Yin

This file contains all reviewer reports in order by version, followed by all author rebuttals in order by version.

Version 0:

Reviewer comments:

Reviewer #1

(Remarks to the Author)
In this paper, the authors developed an engineered, fully recombinant, supercharged protein nanocage called pFn+, which exhibits high cellular uptake efficiency, endosomal escape, and biosafety, enabling the delivery of various proteins such as enzymes and antibodies while maintaining their functionality in vivo. While the authors present substantial data supporting the efficacy of this supercharged interface delivery system, several critical concerns remain unaddressed. Therefore, I recommend rejection at this stage. My major concerns are as follows:
Major Concerns:
1. Introduction: Supercharged interface engineering strategies have been previously reported in both research (PMID: 30643229) and review articles (PMID: 38481692). However, the authors fail to discuss the limitations of supercharged protein-based delivery. Notably, as protein size increases, delivery efficiency typically decreases, which may lead to unintended consequences such as immune responses or altered biological functions. Furthermore, the in vivo study does not assess immune responses, and biosafety, biocompatibility of following injection.
2. In Vivo Study: The authors state that 20 mg/kg/day of Alectinib was orally administered, but they do not specify the amount of pFn+216@Trim21 used per mouse. Additionally, they fail to examine anti-ALK levels in mice or demonstrate whether the antibody successfully reaches the tumor.
3. Immune Response: Given that ferritin comprises a significant portion of the nanocage and has been highly mutated to carry a net charge, the authors should evaluate whether inflammation or immune responses occur in tumor-bearing mice upon administration.
4. Figure 7K and 7L: The authors do not provide quantitative analysis or statistical comparisons between different groups. This is necessary to support their conclusions.
5. Figure 6H: The authors should label images with the antibody name rather than the cell type. Additionally, further investigation into protein distribution and localization within the cytoplasm is needed. Are the proteins trapped in lysosomes, endosomes, or other organelles? What percentage of the protein is successfully released from the endosome?
6. Nanocage Size at Different pH Conditions: The authors should clarify the size of the nanocages and protein-loaded complexes under different pH conditions, as this could significantly impact their stability and delivery efficiency.
7. Primary Cell and Tissue Uptake: Although the study provides data in mice, protein-based delivery often struggles in primary cultured cells and solid tissues. The authors only examine localization in immortalized cell lines, yet primary cells exhibit markedly different uptake efficiencies. This discrepancy should be addressed.
8. Biodistribution and Toxicity (Figure 6C, 6D, 6F, 6G): The data show that 99.9% of the delivered proteins accumulate in the liver and kidney, with minimal presence in other organs. The authors must clarify how much anti-ALK antibody reaches the tumor. Additionally, the nearly complete tumor regression raises concerns—could this be due to pFn+216 toxicity? A control group receiving only pFn+216 should be included.
9. Selective TdTomato Expression (Figure 6F): The authors claim that pFn+216@RNP selectively induces tdTomato expression in specific cell types. However, in Figure 6F, tdTomato expression appears localized to specific areas rather than being randomly distributed throughout the liver. Given that the delivery was intravenous rather than direct liver injection, the authors should explain this pattern.
10. Gene Editing Efficiency (Figure 6H): The authors should assess gene editing efficiency across all major organs to determine the systemic effects of their delivery platform.

Reviewer #2

(Remarks to the Author)
The manuscript presents an innovative strategy for cytosolic protein delivery using supercharged, PBA-modified ferritin nanocages. The design combines several features: genetically encoded unnatural amino acids for reversible, covalent cargo conjugation; tunable surface charge to facilitate cellular uptake; and structural modularity to accommodate diverse cargo proteins. The delivery platform exhibits notable versatility, enabling efficient intracellular transport of various proteins including Cas9, antibodies, and Trim21 complexes. The incorporation of PBA for reversible cargo loading, combined with charge-tunable delivery behavior, is particularly interesting.
However, while the platform is conceptually strong and the data are promising, to meet the publication standards of Nature Communications, the authors need to address several concerns. A major concern is the delivery efficiency experiments lack comparison experiments with the previous methods. Without these comparisons, the practical advantage of this system remains unclear. Therefore, I recommend major revision prior to further consideration.

Major Comments

1. line 27-29, please cite some papers related with polydisulfides faciliated intracellular delivery (Chem. Sci., 2023,14, 2070-2081; J. Am. Chem. Soc. 2020, 142(3), 1217–1221, ect)

2. Lack of references supporting claims:
'Despite these advancements, challenges persist, such 29 as low potency, significant cytotoxicity, lack of generality, and endosomal entrapment.'
'Researchers have proposed strategies to enhance the efficacy of CPPs in delivering larger protein cargos, such as cyclization, which likely optimizes the presentation of positive charges on 34 the cyclic peptide.'

3. Please illustrate the strategy more clearly (in figure 1 and related context), we did not get the reason at the beginning why it's a supercharged strategy? Where does the charge come from?

4. Please clarify the aim of introducing PBA. Is the goal cargo conjugation or covalent integration? The sentence 'To achieve the combination of the supercharged nanocage and cargo proteins, the unnatural amino acid (containing one phenylboronic acid (PBA) side chain) was introduced at the surface of the nanocage' is ambiguous.

5， In Figure 3, the author should give experimental data that show the PBA has function on facilitating binding with Cargoes. As it is well-known that muliti-charged molecules or polymers would induce protein to aggregate, from the current data, it is not feasible to conclude that PBA has worked in the delivery process.

6, Insufficient benchmarking. PULSin is used as the sole comparator. Please include comparisons to additional state-of-the-art delivery platforms (e.g., cationic liposomes, CPPs, LNPs). In Figure 5, the delivery efficiency of pFn+216 and classic CPP such as Arg 9 peptides is suggested to be compared. To demonstrate how advanced for pFn+216，We suggest here also compare other positive charged strategies such as polydisulfide-GFP, which is easy to be synthesized.

7. Lack of in vivo safety data. Only short-term toxicity and body weight monitoring are reported. Additional histopathology of liver, spleen, and kidney beyond H&E is encouraged.

8. Immunogenicity concerns not addressed. High surface charge and unnatural residues may elicit immune responses. A brief discussion or preliminary data (e.g., cytokine assays) would improve confidence for therapeutic use.

9. Please report loading efficiency (e.g., protein-to-cage ratio) and batch-to-batch variability if possible.

10. Trim-Away experiments lack mechanistic controls. Consider adding an isotype antibody control and a catalytically inactive Trim21 mutant to demonstrate that degradation is target- and Trim21-dependent.

Minor Comments

11. Abstract: “good biosafety” is vague. Consider rephrasing.

12. Title suggestion: Consider specifying the system and novelty, e.g., 'Supercharged Ferritin Nanocages Enable Universal Cytosolic Protein.'

13. Charge values should be defined more clearly. The meaning of Fn+216, Fn+312 etc., should be clarified earlier and consistently used.

14. Some sentences are overly long or vague. For example, 'a typical protein nanocage with unnatural amino acids'.

15. Introduction could better differentiate from prior work.

16. Terminology clarification: Use consistent terminology for “cargo protein,” “cargo,” and “delivered protein” to avoid confusion.

17. Sequence verification of PBA incorporation: Mass spec or sequencing validation of the incorporation site is mentioned briefly; please provide more robust evidence.

Reviewer #3

(Remarks to the Author)
I co-reviewed this manuscript with one of the reviewers who provided the listed reports. This is part of the Nature Communications initiative to facilitate training in peer review and to provide appropriate recognition for Early Career Researchers who co-review manuscripts.

Reviewer #4

(Remarks to the Author)
The work addresses a significant challenge in biotechnology—efficient protein delivery into mammalian cells—and proposes a novel platform. The manuscript examines the capabilities of this new platform. However, several concerns need to be addressed to improve the rigor of the claims.

Specific Comments:

Figure 1:
• The schematic is conceptually confusing. The transitions from Fn to Fn+ to pFn+ are presented in a manner that suggests sequential chemical steps, when they are rather strategic stages of design. Consider clarifying with arrows or descriptors.
• The early presentation of “benefits” such as cost-effectiveness, biocompatibility, and efficient delivery appears premature and unsupported at this stage. It risks making the work seem promotional rather than hypothesis-driven.
• The depiction of endosomal escape is both anatomically and mechanistically imprecise. Also, the placement of “in vitro” and “in vivo” labels inside the cell is unconventional and may mislead readers.
• Protein cargos are shown as generic colored ribbons. Using electrostatic potential-based coloring (e.g., red/blue for charge) would better support the narrative around surface charge and isoelectric point relevance.

Figure 2:
• The absence of pFn+ in the panel (despite its relevance to subsequent data) is a missed opportunity for continuity.
• FITC signal interpretation is unclear: is it indicating endocytosis, surface binding, or cytosolic accumulation? Given FITC’s pH sensitivity, endosomal versus cytoplasmic localization will significantly affect signal and interpretation.
• Particle size and aggregation state of the FITC-labeled constructs are not described at this stage of the paper, yet are essential to understanding uptake mechanisms.
• Use of transformed cancer lines (MCF and MDA) is limiting. Inclusion of primary cells would enhance translational relevance and better align with Figure 6.

Figure 3:
• The amount of payload in particles is not quantified. Given the broad range of proteins tested, it is important to determine if loading is sequence- or charge-dependent.
• Figure 3D would benefit from color-coding by protein cargo to assess any hidden trends.
• The rationale for the behavior of highly negatively charged Fn variants is lacking—electrostatic repulsion would apply to negative charges as well. Additional discussion or data are needed to explain aggregation behavior.

Figure 4:
• Panel A implies rapid delivery (within 1–8 min), yet other figures (e.g., 3) rely on 12-hour incubations. These discrepancies should be reconciled or clarified.
• Panel B’s image quality is insufficient to convincingly determine intracellular localization. Live/dead cell indicators should be included to verify viability during imaging.
• Panel C contains the ambiguity of what “cellular uptake” means, especially since FITC may report different compartments.
• Panel D contradicts Panel A, as it shows endosomal trapping at 10 min. Colocalization analysis is not quantitative and should not be used to conclude escape.
• The HiBiT system (Panel E–F) does support cytosolic delivery but there is insufficient evidence that this is resulting from endosomal escape .

Figure 5:
• Panels A–B: It’s not established whether β-Gal or HRP are inactivated by endosomal entrapment. Therefore, the enzymatic readout cannot be definitively attributed to cytosolic activity. It could simply represent enhanced endocytic uptake.
• Panel D: Missing controls for toxicity of the delivery vehicle (pFn+ and PULSin) alone. It is not clear how the concentrations are determine and if the pFN+ or PULSin concentrations are changing as well (or just the payload).
• Panel F: The role of gRNA in Cas9 RNP formation and its impact on particle assembly should be experimentally verified. Cas9 alone is a poor proxy for RNP structure and charge.
• Extended Datas Fig14: these cells don’t look alive. If fixed, this raises concern of the fixation artifact.
• 5I: cells are fixed. This has been reported to create artifacts in cell delivery studies: the laminal staining may happen post fixation. One could rationalize that this doesn’t happen with PULSin. Nonetheless, this leaves the possibility that pFn+ are indeed destabilizing to endosomal escape….not enough for very much escape, but enough to contribute leakage during fixation. PULSin is potentially les active and does not give rise to the artifact. It is for instance puzzling that no endosomes

are visible for pFn+, while they were present in Fig 4D

Figure 7:
• panel B. the microscopy data are qualitative and it is not clear what they provide (with risk of biased selection), given that C provides a quantitative measurement. Control data for pFn+ and PULSin alone are absent in both Panel C and Panel G, yet are essential to interpret specificity and toxicity.

Additional Missing Information:
• If pFn+ successfully enters cells and escapes endosomes, what downstream effects does it have? Is there any stress response? The overall cytotoxicity is assessed, but cellular perturbation post-delivery (e.g., transcriptomic or proteomic signatures) would greatly improve confidence in biocompatibility.

• Figure 6: The in vivo gene editing data presented in Figure 6 show clear hepatic accumulation and functional editing in the liver, which is expected given the route of administration and nanoparticle size. While these results support the potential of pFn+216 for liver-targeted delivery, the impact is limited by the lack of exploration of delivery to other tissues or discussion of potential targeting strategies. The text could be strengthened by acknowledging this limitation and by clarifying whether liver delivery is an intended feature or a starting point for broader applications. A brief discussion of future directions, such as modifying the nanocage or changing administration routes to target other organs, would add important context.

Version 1:

Reviewer comments:

Reviewer #1

(Remarks to the Author)
Although the authors have made substantial revisions in response to the reviewers' comments, several important concerns remain insufficiently addressed. We encourage the authors to revise the manuscript accordingly. Specific comments are as follows:

Figures 7E, 7F, 7L, S22D: Are these blots derived from a single experiment or from independent replicates? At least three biological replicates with appropriate statistical analyses are recommended.

The authors present data on pFn+216@RNP distribution across organs, but it remains unclear which cell types predominantly take up the complex—hepatocytes, Kupffer cells, hepatic stellate cells, or sinusoidal endothelial cells. This clarification is necessary.

Figure 7I: Some tumors appear very small in volume. It would strengthen the findings to include an image of a dissected tumor to better illustrate anti-tumor efficacy. Since intratumoral injections were used, it is also important to examine how the complexes diffuse throughout the entire tumor. We recommend analyzing the intratumoral distribution of the complexes. Moreover, a group treated with Alectinib alone should be included to allow for evaluation of potential synergistic effects.

Figure 7M: Although necrosis and apoptosis were assessed, are these effects specifically attributable to pFn+216@Trim21/anti-ALK delivery? Dual or triple staining (e.g., TUNEL, Trim21/anti-ALK complex, and ALK) would help clarify potential colocalization.

Figure 13: Please specify which cell types were used to evaluate uptake efficiency. In Figure 13A, if cell lysates were analyzed, appropriate internal or loading controls should be included.

While the authors demonstrated typical CPP-GFP delivery, the important supercharged GFP positive control is missing (see PMID: 26465072, 29633093, and 35075948). This control should be included.

Figure S19: The authors claim that cytoplasm-released RNP enters the nucleus via nuclear localization sequences to mediate gene editing. How was this conclusion reached? Since no live-cell imaging was presented, we strongly recommend including real-time imaging to demonstrate whether cytoplasmic RNP complexes indeed enter the nucleus.

Materials and Methods: Additional details are required, including manufacturer information, catalog numbers, reagent concentrations, and staining protocols. Please also clarify the source of the RNPs (lab-made or commercial), whether they contain nuclear targeting sequences, and provide other critical methodological information that is currently missing.

Reviewer #2

(Remarks to the Author)
The authors have satisfactorily addressed all of my concerns. I recommend this manuscript for publication in Nature Communications. I believe that supercharged ferritin nanocages will be of broad interest to the community, given its potential impact on the field of intracellular delivery. I look forward to seeing it published.

Reviewer #3

(Remarks to the Author)
I co-reviewed this manuscript with one of the reviewers who provided the listed reports. This is part of the Nature Communications initiative to facilitate training in peer review and to provide appropriate recognition for Early Career Researchers who co-review manuscripts.

Version 2:

Reviewer comments:

Reviewer #1

(Remarks to the Author)
Although the authors claim that Kupffer cells predominantly take up pFn+216@RNP, as shown in Figure 6J, the quality of the three images (Fig. 6J–6J) is quite poor—particularly the DAPI signal in Figure 6J, which is barely visible. Moreover, the evidence provided by these three single staining images is insufficient to convincingly demonstrate that Kupffer cells are the major cell type responsible for particle uptake. Therefore, I recommend that the authors perform triple staining for CD31/HAS/F4/80 using confocal microscopy to clearly determine which liver cell type exhibits the highest uptake efficiency. Additionally, a flow cytometry–based uptake assessment of these three liver cell populations should be included to quantify their relative uptake percentages.

Regarding the in vivo tumor model, the authors did not address whether the observed antitumor effect is dose dependent.

In Figures 7I and 7J, Alectinib shows almost no effect on tumor size. However, Figure S6 in the referenced preprint (doi: 10.1101/2024.04.24.590626) clearly demonstrates that Alectinib (20 mg/kg/day) reduces H3122 tumor growth after 4–6 days of oral administration. The discrepancy between the present results and previously reported findings requires a thorough explanation, as it raises concerns about whether any procedures or experimental conditions in the current study may not have been adequately designed or controlled.

In the Methods section, antibody dilutions should be clearly stated, and the exact amounts of proteins or complexes used remain missing. The number of mice per group, as well as their sex and age, should also be provided.

Finally, full statistical analyses must be reported for all figures, including details of multiple comparison methods where applicable.

Version 3:

Reviewer comments:

Reviewer #1

(Remarks to the Author)
In line 747, the authors cite reference 40 as the protocol for establishing the H3122/Ale cell line. However, the referenced study used MCF7, MDA-MB-231, and HCT8 cells, none of which are related to the H3122/Ale cell line. Therefore, it remains unclear how the H3122/Ale cell line was generated in this study. Given the well-known issue of cell line misidentification and contamination in the field, the authors should provide cell line authentication data, such as STR profiling or whole-genome sequencing (WGS), to confirm the identity of the H3122/Ale cells.

Several published studies (PMID: 39904499, PMID: 40234387, and PMID: 32041944) have clearly demonstrated, both in vitro and in vivo, that alectinib effectively inhibits the growth of H3122 tumor cells. In contrast, the present manuscript reports that alectinib has no effect on the growth of the H3122 cell line. The authors should clarify this discrepancy. If alectinib truly has no effect, it is also unclear why the mice were treated with alectinib for nearly one month. Moreover, Figure S23 clearly shows that alectinib significantly induces apoptosis in H3122/Ale cells, which further contradicts the stated lack of efficacy.

In Figure 25, although tumor volume was measured in H3122-bearing mice, it remains unknown whether the observed antitumor effect is dependent on EML4-ALK degradation.

In Figure 7, based on the presented data, it is still unclear whether pFn+216 can induce cell death in the presence of alectinib co-administration.

Although the authors examined total EML4-ALK levels, key downstream signaling pathways were not adequately assessed. Specifically, the status of pALK/ALK, pERK/ERK, pAKT/AKT, and pS6/S6 remains unknown, despite these signaling axes being critical regulators of H3122 cell growth and survival.

Given the substantial difference in antitumor efficacy between the pFn+216@Trim21/αALK group and the alectinib group in the mouse model, it is possible that non-ALK-degradation mechanisms contribute to tumor growth inhibition. To address this possibility, administering MG132 (or vehicle control) after pFn+216@Trim21/αALK treatment would help determine whether

the observed tumor suppression is indeed ALK-degradation dependent.

In addition, the authors have not clearly labeled the statistical analysis methods or comparison strategies used in each relevant figure.

Finally, in Figure 21, a proper negative control group is missing. Moreover, the percentage of tdTomato-positive cells within the Cy7-positive population should also be quantified and reported.

Version 4:

Reviewer comments:

Reviewer #1

(Remarks to the Author)
Although the authors have made some revisions, several key concerns remain insufficiently addressed. My specific comments are as follows:

The authors state that alectinib-resistant H3122/Ale cells were used to generate the alectinib-resistant H3122 tumor model. However, according to the Methods, no alectinib was administered before the tumors reached 100 mm^3, so it is unclear how alectinib resistance was maintained in vivo during this period. In addition, it is not justified why 20 mg/kg/day alectinib is sufficient to maintain alectinib resistance in mice. The authors should provide a clear rationale (with references and/or pilot data) for this dosing and explain how resistance was verified and preserved in the animal model.

There is a discrepancy between the description of drug administration in the Methods and the treatment groups shown in Figure 7. In the Methods, it appears that only the control group received oral alectinib, whereas Figure 7 indicates that alectinib was co-administered with other treatments in multiple groups. This inconsistency makes the experimental design difficult to interpret. The authors should clearly describe, for each group, which agents were given, at what dose, route, and schedule, and ensure that the text, Methods, and figures are fully consistent.

In Figure 7L, the six groups are: blank, alectinib only, alectinib + pFn+216, alectinib + Trim21/aALK, alectinib + Trim21/aALK + PULSin, and alectinib + Trim21/aALK + pFn+216. In Figure 7N, the six groups are: blank, alectinib only, pFn+216 only, alectinib + Trim21/aALK, alectinib + Trim21/aALK + PULSin, and alectinib + Trim21/aALK + pFn+216. These group definitions are not consistent between the panels, and it is unclear which design was actually implemented in the animal experiments. The authors need to clarify the exact composition of each group, correct any labeling errors, and, if necessary, revise the figures and legends so that the experimental design is unambiguous.

Although the authors added some data to characterize alectinib resistance in the H3122/Ale cell line, the description in line 353 remains very limited: “Compared to the parental cells, H3122/Ale exhibited upregulation of the efflux transporters ABCB1 and ABCC1 (Extended Data Fig. 23A) and an approximately 16.2-fold increase in alectinib resistance.” It is not explained why ABCB1 and ABCC1 were specifically examined, or what the mechanistic relationship is between these transporters and alectinib resistance. The authors should justify the focus on ABCB1 and ABCC1 with appropriate references (e.g., showing these transporters can limit ALK-TKI brain penetration or contribute to ALK-TKI efflux), and discuss how their upregulation mechanistically links to the observed resistance in H3122/Ale.

Regarding the question of whether the antitumor effect depends on EML4-ALK degradation, the current dose-response experiments are not sufficient to resolve this point. To rigorously test degradation-dependence, the authors should include a control group in which the same amount of complex (e.g., Trim21/aALK + carrier) is administered, but EML4-ALK degradation is selectively blocked (for example, by inhibiting the degradation machinery or using a degradation-defective construct), and then compare antitumor efficacy. Without such a degradation-blocked control, it remains unclear to what extent the observed antitumor effects truly depend on EML4-ALK degradation versus other mechanisms.

For every figure, the legend should clearly specify the statistical analysis used and the comparison strategy (e.g., which groups were compared, one-way vs two-way ANOVA, post hoc tests, exact n, and definition of error bars). This is standard good practice for scientific figures and is necessary for readers to correctly interpret the results. The authors should revise all figure legends accordingly.

Version 5:

Reviewer comments:

Reviewer #1

(Remarks to the Author)
In Figure 2C, it is not clear what the blot represents. Is the detection antibody against Fn, or something else? The authors should state this clearly.

The published reference (PMID: 29153837) clearly suggests that endogenous TRIM21 can mediate protein degradation. As expected in Figure S22E, the GFP level is significantly decreased compared with the PBS and IgG groups; however, in

Figures S22B, S22C, and S22D, the results appear to be inconsistent with Figure S22E. Please clarify why this discrepancy occurs.

In Figures S23E and S23G, the gating strategy appears to differ, even in the PBS group. For similar experiments, the same gating settings are recommended so that the data can be compared more reliably.

In Figure 7M, what is the percentage of TUNEL-positive cells in the randomly selected fields? It appears to be nearly 100% positive. Such a high level often suggests extensive necrosis or technical artifacts rather than pure apoptosis, especially given the large difference between the pFn+216@TRIM21/αALK group and the control groups.

In Figure 7I, what does "CR" mean? The authors should define it clearly in the text.

In Figure 7, it is also recommended to measure the p-AKT/AKT ratio and Bcl-2 expression levels across the different groups.

Open Access This Peer Review File is licensed under a Creative Commons Attribution 4.0 International License, which permits use, sharing, adaptation, distribution and reproduction in any medium or format, as long as you give appropriate credit to the original author(s) and the source, provide a link to the Creative Commons license, and indicate if changes were made.
In cases where reviewers are anonymous, credit should be given to 'Anonymous Referee' and the source.
The images or other third party material in this Peer Review File are included in the article's Creative Commons license, unless indicated otherwise in a credit line to the material. If material is not included in the article's Creative Commons license and your intended use is not permitted by statutory regulation or exceeds the permitted use, you will need to obtain permission directly from the copyright holder.
To view a copy of this license, visit https://creativecommons.org/licenses/by/4.0/

Reviewer #1:

In this paper, the authors developed an engineered, fully recombinant, supercharged protein nanocage called pFn+, which exhibits high cellular uptake efficiency, endosomal escape, and biosafety, enabling the delivery of various proteins such as enzymes and antibodies while maintaining their functionality in vivo. While the authors present substantial data supporting the efficacy of this supercharged interface delivery system, several critical concerns remain unaddressed. Therefore, I recommend rejection at this stage. My major concerns are as follows:

Major Concerns:

1. Introduction: Supercharged interface engineering strategies have been previously reported in both research (PMID: 30643229) and review articles (PMID: 38481692). However, the authors fail to discuss the limitations of supercharged protein-based delivery. Notably, as protein size increases, delivery efficiency typically decreases, which may lead to unintended consequences such as immune responses or altered biological functions. Furthermore, the in vivo study does not assess immune responses, and biosafety, biocompatibility of following injection.

Response:

Thank you for the thoughtful suggestion. First, we carefully reviewed the referenced study (PMID: 30643229) and found that although it also employs a supercharging strategy, it is fundamentally different from our surface supercharging approach in terms of experimental design, methodology, and intended application. Specifically, the referenced work introduces both negatively and positively charged mutations into proteins to generate oppositely charged variants, which are then assembled into diverse supramolecular structures. As clearly stated in their abstract - "The ready assembly of this structure suggests that combining oppositely supercharged pairs of protein variants may provide broad opportunities for generating novel architectures via otherwise unprogrammed interactions" - the primary goal of their approach is to explore de novo protein assembly. By contrast, our surface supercharging strategy is explicitly designed to enable efficient intracellular delivery of functional protein payloads. Rather than leveraging charge complementarity for structural assembly, we engineer ultrahigh surface charge density on a single protein scaffold to enhance cellular uptake and facilitate endosomal escape, with a clear focus on functional cytosolic delivery.

Further, we fully recognize that conventional supercharged protein carriers face intrinsic limitations, most notably the inverse correlation between cargo size and delivery efficiency - a widely reported challenge primarily stemming from steric hindrance and endosomal entrapment (PMID: 38481692). However, our engineered pFn+216 platform fundamentally diverges from previous systems through two key innovations. First, by achieving a net positive charge of up to +312 per nanocage - nearly 10-fold higher than the most advanced previously reported variants (e.g., +32 in PMID: 30643229) - we overcome the electrostatic energy barriers that have traditionally limited the internalization of large cargoes, enabling efficient delivery of multi-component complexes up to 430 kDa (Fig. 3C). Second, the highly charged nanocomplexes (~100 nm) exhibit excellent cellular uptake, and upon internalization, they dissociate into ~12 nm monomers within the acidic endosomal environment, thereby minimizing steric hindrance and preserving cargo bioactivity (Fig. 4G). Moreover, we have expanded the discussion in the revised manuscript and included both relevant references accordingly.

To address concerns regarding the safety of this system, our study provides multidimensional safety profiling

spanning from *in vitro* to *in vivo* models. For cytocompatibility, pFn+216 exhibits no significant cytotoxicity across six cell lines (Extended Data Fig. 15A-F). Hemocompatibility assessments showed no significant hemolysis at concentrations up to 10 μM (Extended Data Fig. 15G). For organ toxicity, histopathological assessment of major organs (liver, kidney, spleen, heart, lung) reveals no abnormalities - including absence of inflammatory infiltrates, fibrosis, or necrosis - after repeated intravenous dosing (Extended Data Fig. 16A-C). For immunogenicity, serum cytokine profiling (IL-6, TNF-α, IFN-γ) shows no elevation post-injection (Extended Data Fig. 16D). RNA-seq analysis comparing pFn+216-treated and vehicle control cells identified 118 significantly differentially expressed genes (Extended Data Fig. 17A). Crucially, gene set enrichment analysis (GSEA) showed no enrichment in canonical stress-response or cell death pathways (Extended Data Fig. 17B-C).

The original submission data are marked (blue colored in the bracket), and supplementary experimental data are marked (green colored in the bracket), and revised parts are shown below (red colored in the text). The corresponding passages in the manuscript are marked in the same manner.

In "Results - Study on the cellular entry of cage/cargo protein complex" Section

We further conducted multidimensional safety evaluations of the system across both *in vitro* and *in vivo* models. For cytocompatibility, pFn+216 exhibits no significant cytotoxicity (Extended Data Fig. 15A-F). Hemocompatibility assessments showed no significant hemolysis at concentrations up to 10 μM (Extended Data Fig. 15G). The *in vivo* safety assessment encompassed acute toxicity screening (14-day observation), multi-organ histopathology (H&E staining of liver, spleen, kidney, and heart; Extended Data Fig. 16A), serum biochemistry (ALT/AST for liver function, BUN/creatinine for kidney function; Extended Data Fig. 16B-C), and cytokine profiling (Extended Data Fig. 16D)—provides comprehensive evidence supporting the biosafety of pFn+216 at therapeutically relevant doses. To further evaluate potential cellular stress responses induced by pFn+216-mediated protein delivery, we conducted comprehensive transcriptomic profiling. RNA-seq analysis comparing pFn+216-treated and vehicle control cells identified 118 significantly differentially expressed genes (DEGs), comprising 38 upregulated and 80 downregulated transcripts (Extended Data Fig. 17A). Crucially, gene set enrichment analysis (GSEA) showed no enrichment in canonical stress-response or cell death pathways (e.g., p53 signaling, apoptosis, ER stress). Gene Ontology (GO) analysis indicated the DEGs were primarily associated with physiological regulatory processes, including voltage-gated ion channel activity, cell-cell junction assembly, and basolateral plasma membrane organization, consistent with homeostatic cellular adaptation (Extended Data Fig. 17B). KEGG analysis further confirmed enrichment solely in benign functional pathways, most notably taste transduction (Extended Data Fig. 17C). Collectively, these preliminary findings support the potential of pFn+216 as an effective and safe protein delivery carrier.

In "Discussion" Section

In this study, we present a supercharged protein nanocage engineering strategy that enables efficient cytosolic delivery of diverse functional proteins. By systematically redesigning the surface charge landscape of Fn nanocages through in silico-guided mutagenesis, we generated a library of Fn+ variants with charges spanning from -168 to +312. Crucially, we demonstrate a charge-dependent cellular internalization mechanism: while low-charge Fn+ relies on CD71-mediated endocytosis, variants with net surface charges exceeding +168 bypass receptor dependency and achieve charge-dominated uptake. This shift from receptor-mediated to charge-driven entry significantly expands the

utility of the Fn platform, opening avenues for the delivery of a wider range of therapeutic cargoes beyond natural binding partners.

To achieve universal, non-conjugative cargo protein loading, we pioneered the site-specific incorporation of PBA into pFn+ via genetic code expansion. pFn+ dynamically self-assembles with cargo proteins, but complex formation is exquisitely charge-dependent: moderately charged variants (pFn+120 to pFn+216) form stable ~100 nm nanocomplexes, whereas excessive charge (>+240) disrupts assembly through electrostatic repulsion. This optimal charge density range—peaking at pFn+216—balances nanocomplex stability and cellular uptake efficiency, enabling superior endosomal escape and functional delivery that outperforms commercial reagents like PULSin™. The system demonstrates exceptional safety due to the biocompatible Fn scaffold and reversible cargo protein binding, resulting in negligible cytotoxicity, hemolysis, and organ toxicity. In addition, the system is highly scalable, enabling efficient expression in *E. coli* (unoptimized yield >50 mg/L) without the need for complex chemical modifications.

Notably, *in vivo* gene editing via systemic administration achieved highly efficient, liver-specific tdTomato activation in *Ai14* mice, leveraging the inherent hepatic first-pass accumulation of nanoparticles. Furthermore, successful antibody-guided intracellular protein degradation (Trim-Away) was demonstrated for both GFP and the oncogenic driver EML4-ALK; therapeutic pFn+216 nanoparticles encapsulating Trim21/anti-ALK complexes effectively suppressed tumor growth *in vivo*, validating intracellular antibody functionality. Beyond these demonstrated applications, the supercharged interface engineering strategy underlying pFn+216 offers distinct advantages over existing platforms. It exhibits remarkable versatility, efficiently delivering proteins ranging from 13 to 430 kDa, with pI spanning 5.0 to 9.5, including complex multi-component cargos such as Cas9/sgRNA ribonucleoproteins and Trim21/antibody complexes.

In our *in vivo* gene editing studies, we observed predominant accumulation of pFn+216@cargo protein complexes in the liver, which presents a substantial advantage for the development of therapeutics targeting liver-associated diseases. Nonetheless, expanding the targeting scope beyond the liver represents a key direction for future optimization. This could be achieved through fusion with tissue-specific homing peptides or small targeting moieties such as nanobodies or single-chain antibodies. Alternatively, surface charge masking strategies—for example, shielding positive charges with PEG—could enable conditional exposure of the supercharged surface in specific tissue microenvironments to achieve localized delivery. Moreover, the intrinsic cavity of Fn itself offers opportunities for cargo protein loading[38] (e.g., metal ions or small molecules such as doxorubicin), enabling the development of multi-cargo co-delivery strategies that fully leverage the native properties of the Fn scaffold. Finally, while our supercharged interface engineering strategy has thus far been applied only to Fn, future studies may extend this approach to other natural or synthetic protein nanocages[39], providing valuable guidance for the design of next-generation intracellular delivery vehicles with tunable surface or exposed charge properties.

2. In Vivo Study: The authors state that 20 mg/kg/day of Alectinib was orally administered, but they do not specify the amount of pFn+216@Trim21 used per mouse. Additionally, they fail to examine anti-ALK levels in mice or demonstrate whether the antibody successfully reaches the tumor.

Response:

We have now explicitly stated in the revised Methods section: "Mice were administered intratumoral injections of saline, pFn+216, Trim21/anti-ALK, PULSin@Trim21/anti-ALK, or pFn+216@Trim21/anti-ALK complex per mouse (equivalent to 1 μg anti-ALK IgG) every 4 days. In the pFn+216@Trim21/anti-ALK complex, the optimized molar ratio of the three components is 1:8:8 (pFn+216:Trim21: IgG)." To address concerns regarding the successful delivery of antibodies to tumor tissue, we performed Western blot analysis on tumor lysates (Fig. 7L; an expanded version of Extended Data Fig. 16B from the previous manuscript, now including additional control groups). This directly demonstrates effective intratumoral antibody delivery and functional activity, as evidenced by a marked reduction in EML4-ALK protein levels in the pFn+216@Trim21/anti-ALK treatment group compared to saline controls.

The supplementary experimental data are marked (green colored in the bracket), and revised parts are shown below (red colored in the text). The corresponding passages in the manuscript are marked in the same manner.

In "Methods - In vivo mouse models and treatments" Section

Mice were administered intratumoral injections of saline, pFn+216, Trim21/anti-ALK, PULSin@Trim21/anti-ALK, or pFn+216@Trim21/anti-ALK complex per mouse (equivalent to 1 μg anti-ALK IgG) every 4 days. In the pFn+216@Trim21/anti-ALK complex, the optimized molar ratio of the three components is 1:8:8 (pFn+216:Trim21:IgG).

3. Immune Response: Given that ferritin comprises a significant portion of the nanocage and has been highly mutated to carry a net charge, the authors should evaluate whether inflammation or immune responses occur in tumor-bearing mice upon administration.

Response:

As tumor-bearing mice received intratumoral injections, we conducted immunological safety assessments in healthy mice subjected to repeated high-dose intravenous administration of pFn+216 to comprehensively evaluate potential systemic risks. Histopathological examination of major organs (liver, spleen, kidney, lung) revealed no evidence of inflammatory infiltrates, granuloma formation, or tissue necrosis in H&E-stained sections (Extended Data Fig.16A), indicating absence of acute tissue inflammation. Serum biochemistry analysis further confirmed systemic biocompatibility, with alanine aminotransferase (ALT) and aspartate aminotransferase (AST) levels remaining within normal physiological ranges (Extended Data Fig.16B), demonstrating preserved hepatic function. Renal safety was similarly validated through stable blood urea nitrogen (BUN) and creatinine levels (Extended Data Fig.16C). Most critically, multiplex cytokine analysis of serum samples collected post-injection showed no elevation in key pro-inflammatory mediators including IL-6, TNF-α, and IL-1β (Extended Data Fig.16D), effectively ruling out cytokine storm responses.

The supplementary experimental data are marked (green colored in the bracket), and revised parts are shown below (red colored in the text). The corresponding passages in the manuscript are marked in the same manner.

In "Results - Study on the cellular entry of cage/cargo protein complex" Section

The *in vivo* safety assessment encompassed acute toxicity screening (14-day observation), multi-organ histopathology (H&E staining of liver, spleen, kidney, and heart; Extended Data Fig. 16A), serum biochemistry (ALT/AST for liver function, BUN/creatinine for kidney function; Extended Data Fig. 16B-C), and cytokine profiling (Extended Data Fig. 16D)—provides comprehensive evidence supporting the biosafety of pFn+216 at therapeutically relevant doses.

4. Figure 7K and 7L: The authors do not provide quantitative analysis or statistical comparisons between different groups. This is necessary to support their conclusions.

Response:

We have now supplemented quantitative analysis for TUNEL staining. Histopathological analysis confirmed the tumor cell death-inducing effect of pFn+216@Trim21/anti-ALK (Fig. 7M). The co-delivery of pFn+216@Trim21/anti-ALK achieved a higher amount of apoptosis (Fig. 7N), significantly outperforming commercial transfection reagents.

The supplementary experimental data are marked (green colored in the bracket), and revised parts are shown below (red colored in the text). The corresponding passages in the manuscript are marked in the same manner.

In "Results - Antibody-Mediated Degradation of Endogenous Proteins" Section

Histopathological analysis employing H&E staining and TUNEL assays revealed that the pFn+216@Trim21/anti-ALK treatment group exhibited significantly larger areas of necrosis and a higher incidence of apoptosis compared to the other groups (Fig. 7M). Quantitative analysis of the TUNEL assays further confirmed that the co-delivery of pFn+216@Trim21/anti-ALK induced a significantly higher level of apoptosis (Fig. 7N), markedly outperforming commercial transfection reagents.

5. Figure 6H: The authors should label images with the antibody name rather than the cell type. Additionally, further investigation into protein distribution and localization within the cytoplasm is needed. Are the proteins trapped in lysosomes, endosomes, or other organelles? What percentage of the protein is successfully released from the endosome?

Response:

In the revised Fig. 6H-J, all fluorescence labels have been updated to explicitly indicate the specific antibodies used: anti-CD31 (green) for endothelial cells, anti-HSA (green) for hepatocytes, and anti-F4/80 (green) for Kupffer cells, with anti-RFP (red) marking tdTomato expression in all panels. Regarding cytoplasmic protein distribution, our comprehensive analysis demonstrates efficient endosomal escape and functional cytosolic release. Confocal microscopy revealed that the delivered GFP exhibited a predominantly diffuse cytoplasmic distribution, with only a minor fraction colocalizing with lysosomes (Fig. 4D). Crucially, quantitative assessment using HiBiT-LgBiT luciferase assays (Fig. 4E-F) confirms that 36.3% of internalized proteins achieve cytosolic release - 10.7-fold higher

than PULSin controls (3.4 %, P<0.001). Functional assays following intracellular delivery of diverse protein cargos revealed robust biological activity, indirectly supporting successful endosomal escape of a substantial fraction of the internalized proteins (Fig. 5-7).

The original submission data are marked (blue colored in the bracket), and supplementary experimental data are marked (green colored in the bracket), and revised parts are shown below (red colored in the text). The corresponding passages in the manuscript are marked in the same manner.

In "Figure Legend" section of Fig. 6H-J:

Liver sections were stained with anti-RFP antibody for tdTomato (red), an anti-CD31 antibody for endothelial cells (green in H), an anti-HAS (hepatocyte-specific antigen) antibody (green in I), an anti-F4/80 antibody for Kupffer cells (green in J), and DAPI for nucleus (blue).

In "Results - Study on the cellular entry of cage/cargo protein complex" Section

The colocalization of the delivered GFP with lysotracker (lysosome marker) was studied. Colocalization analysis indicated that at 1 hours, GFP was diffusely distributed throughout the cytoplasm (Fig. 4D). In parallel, we employed the HiBiT-LgBit system to quantitatively evaluate lysosomal escape efficiency[30,31]. This method specifically measures cytoplasmic HiBiT peptide fluorescence relative to total cellular fluorescence (Fig. 4E). The results indicate that the lysosomal escape efficiency of pFn+216@HiBit (36.3%) is significantly higher than that of PULSin@HiBit (3.4%). Meanwhile, compared to PULSin, the total intracellular uptake and cytoplasmic quantity were approximately 2.0-fold and 20.7-fold higher, respectively (Fig. 4F). Chloroquine treatment significantly enhanced pFn+216-mediated delivery, further indicating endosomal involvement (Extended Data Fig. 12C). Collectively, these results indicate that pFn+216 facilitates efficient escape of its cargo from early endosomes prior to lysosomal degradation, leading to significant accumulation within the cytoplasm.

6. Nanocage Size at Different pH Conditions: The authors should clarify the size of the nanocages and protein-loaded complexes under different pH conditions, as this could significantly impact their stability and delivery efficiency.

Response:

We thank the reviewer for highlighting the importance of pH-dependent behavior for intracellular delivery systems. As shown in Fig. 4G, we have already provided direct experimental evidence that pFn+216@GFP complexes undergo pH-triggered disassembly. Our data demonstrate that the size of the complex was approximately 100 nm at pH 7.4, but decreased to approximately 10 nm when the pH was below 6.5, presumably due to the disruption of intermolecular interactions leading to complex disassembly. In addition, control experiments confirm that only pFn+216 exhibit no significant size change across pH 5.0-7.4, demonstrating that the pH-responsive behavior is exclusive to the pFn+216@cargo protein complexes (Extended Data Fig. 11C).

The original submission data are marked (blue colored in the bracket), and supplementary experimental data are marked (green colored in the bracket), and revised parts are shown below (red colored in the text). The corresponding passages in the manuscript are marked in the same manner.

In "Results - Study on the cellular entry of cage/cargo protein complex" Section

In addition, control experiments confirm that only pFn+216 exhibit no significant size change across pH 5.0-7.4, demonstrating that the pH-responsive behavior is exclusive to the pFn+216@cargo protein complexes (Extended Data Fig. 11C).

7. Primary Cell and Tissue Uptake: Although the study provides data in mice, protein-based delivery often struggles in primary cultured cells and solid tissues. The authors only examine localization in immortalized cell lines, yet primary cells exhibit markedly different uptake efficiencies. This discrepancy should be addressed.

Response:

We are grateful for this valuable critique regarding the translational relevance of protein delivery platforms. To directly address the concern about primary cell efficiency, we have performed new experiments on primary murine cells (T lymphocytes and hepatocytes). While primary cells often exhibit reduced uptake for protein carriers, pFn+216 maintains high efficiency in primary T cells and hepatocytes (Extended Data Fig. 12A). This broad applicability may stem from its ultrahigh-charge-driven internalization mechanism, which bypasses reliance on cell type–specific surface receptors.

The supplementary experimental data are marked (green colored in the bracket), and revised parts are shown below (red colored in the text). The corresponding passages in the manuscript are marked in the same manner.

In "Results - Study on the cellular entry of cage/cargo protein complex" Section

This efficient delivery mediated by pFn+216 was remarkably consistent across multiple diverse cell lines (MDA-MB-231, C6, A549, PC12, BEL-7402, and Jurkat cells) and notably effective in transducing hard-to-transfect primary cells such as T lymphocytes and hepatocytes, highlighting its utility as a delivery vehicle (Extended Data Fig. 12A).

8. Biodistribution and Toxicity (Figure 6C, 6D, 6F, 6G): The data show that 99.9% of the delivered proteins accumulate in the liver and kidney, with minimal presence in other organs. The authors must clarify how much anti-ALK antibody reaches the tumor. Additionally, the nearly complete tumor regression raises concerns—could this be due to pFn+216 toxicity? A control group receiving only pFn+216 should be included.

Response:

We have clearly indicated in both the figure legends and Methods section that the biodistribution data in Fig. 6 were obtained following intravenous administration, whereas the antitumor efficacy shown in Fig. 7 was assessed using intratumoral injection. For the tumor therapy study, localized intratumoral delivery was employed to maximize bioavailability at the target site—this route bypasses systemic circulation. To definitively exclude the possibility that tumor regression was caused by carrier-related toxicity, we included an additional control group receiving pFn+216 alone in the revised Fig. 7H-N. Tumor growth kinetics in this group were indistinguishable from those in the saline-

treated group, confirming that tumor suppression was exclusively mediated by the functional payload (Trim21/anti-ALK). Furthermore, Western blot analysis further confirmed that pFn+216@Trim21/anti-ALK effectively reduced EML4-ALK levels *in vivo* (Fig. 7L).

The original submission data are marked (blue colored in the bracket), and supplementary experimental data are marked (green colored in the bracket), and revised parts are shown below (red colored in the text). The corresponding passages in the manuscript are marked in the same manner.

In "Results - Antibody-Mediated Degradation of Endogenous Proteins" Section

To further evaluate *in vivo* efficacy, H3122 tumor-bearing mice received intratumoral injections of pFn+216@Trim21/anti-ALK (Fig. 7H). Tumor growth inhibition was significantly greater in the pFn+216@Trim21/anti-ALK group compared to the PULSin@Trim21/anti-ALK, Trim21/anti-ALK alone, pFn+216 alone and saline control groups (Fig. 7I, J). Body weights remained stable across all treatment groups, indicating no systemic toxicity (Fig. 7K). Furthermore, Western blot analysis confirmed that pFn+216@Trim21/anti-ALK effectively reduced EML4-ALK levels *in vivo* (Fig. 7L). Histopathological analysis employing H&E staining and TUNEL assays revealed that the pFn+216@Trim21/anti-ALK treatment group exhibited significantly larger areas of necrosis and a higher incidence of apoptosis compared to the other groups (Fig. 7M). Quantitative analysis of the TUNEL assays further confirmed that the co-delivery of pFn+216@Trim21/anti-ALK induced a significantly higher level of apoptosis (Fig. 7N), markedly outperforming commercial transfection reagents.

9. Selective TdTomato Expression (Figure 6F): The authors claim that pFn+216@RNP selectively induces tdTomato expression in specific cell types. However, in Figure 6F, tdTomato expression appears localized to specific areas rather than being randomly distributed throughout the liver. Given that the delivery was intravenous rather than direct liver injection, the authors should explain this pattern.

Response:

We appreciate the reviewer's insightful comment regarding the non-uniform tdTomato expression pattern observed in Fig. 6F. Indeed, the spatially localized expression reflects not random variability but rather the intrinsic zonation architecture of liver lobules and the trafficking dynamics of systemically administered nanoparticles. Specifically, intravenous delivery results in first-pass distribution through the hepatic portal circulation, where nanoparticles encounter hepatocytes in a zonated fashion as they traverse the sinusoidal network. This results in preferential exposure of certain hepatocyte subpopulations - particularly pericentral or periportal zones, depending on particle size, charge, and surface properties. Such heterogeneous gene editing patterns have been widely reported for diverse nanoparticle systems following systemic administration [1-3].

Reference:

[1] Chen K, Han H, Zhao S, Xu B, Yin B, Lawanprasert A, Trinidad M, Burgstone BW, Murthy N, Doudna JA. Lung and liver editing by lipid nanoparticle delivery of a stable CRISPR-Cas9 ribonucleoprotein. Nat Biotechnol. 2024 Oct 16:10.1038/s41587-024-02437-3.

[2] Radmand A, Kim H, Beyersdorf J, Dobrowolski CN, Zenhausern R, Paunovska K, Huayamares SG, Hua X, Han K, Loughrey D, Hatit MZC, Del Cid A, Ni H, Shajii A, Li A, Muralidharan A, Peck HE, Tiegreen KE, Jia S, Santangelo PJ, Dahlman JE. Cationic cholesterol-dependent LNP delivery to lung stem cells, the liver, and heart. Proc Natl Acad Sci U S A. 2024 Mar 12;121(11):e2307801120.
[3] Xie R, Wang X, Wang Y, Ye M, Zhao Y, Yandell BS, Gong S. pH-Responsive Polymer Nanoparticles for Efficient Delivery of Cas9 Ribonucleoprotein With or Without Donor DNA. Adv Mater. 2022 Jun;34(23):e2110618.

10. Gene Editing Efficiency (Figure 6H): The authors should assess gene editing efficiency across all major organs to determine the systemic effects of their delivery platform.

Response:

Our experimental design focused on validating the liver-targeting capability of pFn+216, a property intrinsically linked to its nanoparticle size. The biodistribution data (Fig. 6B-D) demonstrates that intravenously injected pFn+216@RNP accumulates in the liver within 24 hours, with negligible retention in other major organs (heart, lung, spleen, brain). This profound hepatic tropism - consistent with the known filtration behavior of nanoparticles by liver sinusoidal endothelial cells [1] - provides a clear rationale for prioritizing functional validation in the liver rather than systemically. In addition, following the reviewer's suggestion, we evaluated non-hepatic tissues (kidney, heart, lung, spleen) from pFn+216@RNP-treated mice and found minimal fluorescence accumulation (Extended Data Fig. 21). These results are consistent with the low levels of tissue retention observed in the biodistribution studies. The subsequent functional analysis (Fig. 6F-H) specifically examines tdTomato expression within liver cell populations, as biodistribution data clearly identified the liver as the primary site of nanoparticle accumulation.

The original submission data are marked (blue colored in the bracket), and supplementary experimental data are marked (green colored in the bracket), and revised parts are shown below (red colored in the text). The corresponding passages in the manuscript are marked in the same manner.

In "pFn+216@RNP complex effectively edits organs *in vivo*" Section

Immunofluorescence staining further revealed concentrated fluorescence accumulation in the liver for pFn+216@RNP, with minimal fluorescence elsewhere (Extended Data Fig. 21). In contrast, the PULSin@RNP group exhibited significantly lower hepatic accumulation but markedly higher expression in the kidneys. This biodistribution profile indicates that pFn+216@RNP achieves unexpectedly liver-targeted delivery compared to both RNP and PULSin@RNP.

Reference:

[1] Wunsch BH, *et al*. Nanoscale lateral displacement arrays for the separation of exosomes and colloids down to 20 nm. Nat Nanotechnol. 2016 Nov;11(11):936-940.

Reviewer #2:

The manuscript presents an innovative strategy for cytosolic protein delivery using supercharged, PBA-modified ferritin nanocages. The design combines several features: genetically encoded unnatural amino acids for reversible, covalent cargo conjugation; tunable surface charge to facilitate cellular uptake; and structural modularity to accommodate diverse cargo proteins. The delivery platform exhibits notable versatility, enabling efficient intracellular transport of various proteins including Cas9, antibodies, and Trim21 complexes. The incorporation of PBA for reversible cargo loading, combined with charge-tunable delivery behavior, is particularly interesting. However, while the platform is conceptually strong and the data are promising, to meet the publication standards of Nature Communications, the authors need to address several concerns. A major concern is the delivery efficiency experiments lack comparison experiments with the previous methods. Without these comparisons, the practical advantage of this system remains unclear. Therefore, I recommend major revision prior to further consideration.

Major Comments

1. line 27-29, please cite some papers related with polydisulfides faciliated intracellular delivery (Chem. Sci., 2023,14, 2070-2081; J. Am. Chem. Soc. 2020, 142(3), 1217−1221, ect)

Response:

Thank you for the suggestion. We have incorporated these two key references to seminal studies on polydisulfide-mediated cytosolic delivery systems in the revised manuscript.

2. Lack of references supporting claims: 'Despite these advancements, challenges persist, such 29 as low potency, significant cytotoxicity, lack of generality, and endosomal entrapment.'

'Researchers have proposed strategies to enhance the efficacy of CPPs in delivering larger protein cargos, such as cyclization, which likely optimizes the presentation of positive charges on 34 the cyclic peptide.'

Response:

We have strengthened the manuscript by incorporating key references to support the critical claims.

3. Please illustrate the strategy more clearly (in figure 1 and related context), we did not get the reason at the beginning why it's a supercharged strategy? Where does the charge come from?

Response:

We appreciate the reviewer's request for clarification regarding the molecular basis of our supercharged strategy. The enhanced positive surface charge of pFn+ variants is achieved through a two-step protein engineering process explicitly detailed in the revised manuscript. First, site-directed mutagenesis replaces solvent-exposed anionic residues (aspartic acid [D] and glutamic acid [E]) with cationic residues (lysine [K] or arginine [R]) at 13 spatially

optimized positions on each ferritin monomer. Second, genetic code expansion (GCE) technology incorporates the unnatural amino acid 4-boronophenylalanine (4-BPA) at a defined surface site, appending phenylboronic acid (PBA) moieties without altering the net positive charge density. This dual-modification approach achieves two synergistic objectives: (i) the engineered cationic surface enables electrostatic-driven cellular uptake and endosomal escape, while (ii) the PBA groups mediate reversible cargo conjugation through boronate ester bonds. The integration of these elements - depicted schematically in the revised Fig. 1 - establishes pFn+ as a unique platform where charge-driven delivery and dynamic cargo release are genetically encoded into a single nanocage architecture.

The revised parts are shown below (red colored in the text). The corresponding passages in the manuscript are marked in the same manner.

In "Figure Legend" section of Fig. 1:

This platform utilizes a ferritin (Fn) nanocage, engineered through a two-step protein engineering strategy to achieve supercharged interfaces. The supercharged interface engineering strategy is achieved through a two-step protein engineering process. First, site-directed mutagenesis replaces solvent-exposed anionic residues (aspartic acid [D] and glutamic acid [E]) with cationic residues (lysine [K] or arginine [R]) at optimized positions on each ferritin monomer, yielding Fn+. Second, genetic code expansion (GCE) technology incorporates the unnatural amino acid 4-boronophenylalanine (4-BPA) at a defined surface site, appending phenylboronic acid (PBA) moieties, yielding pFn+. This pFn+ nanocage can be efficiently produced at scale in a cost-effective manner using an *E. coli* expression system. The platform enables the straightforward formation of complexes through simple mixing with various protein cargoes. These complexes are subsequently internalized by mammalian cells with high efficiency and released into the cytoplasm, where they exert their biological functions both *in vitro* and *in vivo*.

4. Please clarify the aim of introducing PBA. Is the goal cargo conjugation or covalent integration? The sentence 'To achieve the combination of the supercharged nanocage and cargo proteins, the unnatural amino acid (containing one phenylboronic acid (PBA) side chain) was introduced at the surface of the nanocage' is ambiguous.

Response:

The primary goal of introducing PBA is to enable specific and reversible cargo conjugation through dynamic boronate ester bonding, rather than permanent covalent integration [1-3].

The revised parts are shown below (red colored in the text). The corresponding passages in the manuscript are marked in the same manner.

Reference:

[1] Liu X, Zhao Z, Wu F, Chen Y, Yin L. Tailoring Hyperbranched Poly(β-amino ester) as a Robust and Universal Platform for Cytosolic Protein Delivery. Adv Mater. 2022 Feb;34(8):e2108116.

[2] Liu C, Wan T, Wang H, Zhang S, Ping Y, Cheng Y. A boronic acid-rich dendrimer with robust and unprecedented efficiency for cytosolic protein delivery and CRISPR-Cas9 gene editing. Sci Adv. 2019 Jun 12;5(6):eaaw8922.

[3] Wang Q, Yang Y, Liu D, Ji Y, Gao X, Yin J, Yao W. Cytosolic Protein Delivery for Intracellular Antigen Targeting

Using Supercharged Polypeptide Delivery Platform. Nano Lett. 2021 Jul 28;21(14):6022-6030.

In "Introduction" section:

To enable reversible covalent conjugation between the supercharged nanocage and cargo proteins, we genetically incorporated a phenylboronic acid (PBA)[21,22]-bearing unnatural amino acid (UAA) at solvent-exposed sites of the nanocage.

5. In Figure 3, the author should give experimental data that show the PBA has function on facilitating binding with Cargoes. As it is well-known that muliti-charged molecules or polymers would induce protein to aggregate, from the current data, it is not feasible to conclude that PBA has worked in the delivery process.

Response:

The pivotal role of PBA in mediating cargo binding is unambiguously demonstrated through controlled experiments comparing modified (pFn+216) and unmodified (Fn+216) nanocages. DLS analysis confirms that PBA functionalization is essential for forming stable, monodisperse complexes (Extended Data Fig. 9A). Confocal microscopy and flow cytometry reveal that only PBA-modified nanocages achieve efficient intracellular GFP delivery, whereas unmodified Fn+216 shows negligible uptake despite identical surface charge (Extended Data Fig. 9B-D).

The supplementary experimental data are marked (green colored in the bracket), and revised parts are shown below (red colored in the text). The corresponding passages in the manuscript are marked in the same manner.

In "Results - Screening of nanocage/cargo protein complex" Section

To determine whether PBA incorporation determines protein-binding capacity of Fn+216, we compared the cellular delivery efficiency of GFP mediated by Fn+216 versus pFn+216. PBA-modified Fn+216 formed stable, monodisperse complexes, with an average hydrodynamic diameter of approximately 100 nm (Extended Data Fig. 9A). Moreover, only pFn+216 successfully facilitated intracellular GFP delivery (Extended Data Fig. 9B-D), indicating that PBA modification is essential for enabling Fn+216 to functionally interact with cargo proteins.

6. Insufficient benchmarking. PULSin is used as the sole comparator. Please include comparisons to additional state-of-the-art delivery platforms (e.g., cationic liposomes, CPPs, LNPs). In Figure 5, the delivery efficiency of pFn+216 and classic CPP such as Arg 9 peptides is suggested to be compared. To demonstrate how advanced for pFn+216， We suggest here also compare other positive charged strategies such as polydisulfide-GFP, which is easy to be synthesized.

Response:

We thank the reviewer for the valuable suggestion to broaden our benchmarking against state-of-the-art protein delivery systems. While PULSin was initially used as a representative commercial transfection reagent, we have

substantially expanded our comparative framework to rigorously evaluate pFn+216 against multiple cutting-edge platforms.

This comprehensive analysis reveals two major advances: First, unlike cationic liposomes (e.g., Lipofectamine CRISPRMAX), which require stringent cargo-specific charge compatibility—achieving high efficiency only for negatively charged proteins such as (-30)GFP-Cas9 while failing with positively charged payloads [1-2] - pFn+216 enables charge-independent delivery across the full isoelectric point (pI) range (5.0–9.5), including notoriously difficult neutral or positively charged proteins. Second, in direct comparisons with ten classical and non-classical CPPs (R9, TAT, DPV1047, MPG, PTD4, Penetratin, TP2, dNP2, Pep1, and SCP) fused to GFP (Extended Data Fig. 13-14 and Table S1), pFn+216 consistently outperformed all candidates in intracellular GFP delivery efficiency.

The supplementary experimental data are marked (green colored in the bracket), and revised parts are shown below (red colored in the text). The corresponding passages in the manuscript are marked in the same manner.

In "Results- Study on the cellular entry of cage/cargo protein complex" Section

In direct comparison with a panel of established CPPs showed that pFn+216 significantly outperformed all tested candidates in intracellular GFP delivery efficiency (Extended Data Fig. 13-14 and Table S1).

[1] Zuris JA, Thompson DB, Shu Y, Guilinger JP, Bessen JL, Hu JH, Maeder ML, Joung JK, Chen ZY, Liu DR. Cationic lipid-mediated delivery of proteins enables efficient protein-based genome editing in vitro and in vivo. Nat Biotechnol. 2015 Jan;33(1):73-80.

[2] Lv J, Fan Q, Wang H, Cheng Y. Polymers for cytosolic protein delivery. Biomaterials. 2019 Oct;218:119358.

7. Lack of in vivo safety data. Only short-term toxicity and body weight monitoring are reported. Additional histopathology of liver, spleen, and kidney beyond H&E is encouraged.

Response:

While this study primarily focuses on establishing the foundational mechanistic efficacy and platform versatility of pFn+216 as a protein delivery tool, our current safety assessment - spanning acute toxicity screening (14-day observation), multi-organ histopathology (H&E of liver/spleen/kidney/heart) (Extended Data Fig. 16A), serum biochemistry (ALT/AST for liver, BUN/creatinine for kidney) (Extended Data Fig. 16B-C), and cytokine profiling (Extended Data Fig. 16D) - provides robust evidence of biosafety at therapeutically relevant doses. These results confirm the absence of overt pathological abnormalities, organ dysfunction, or systemic inflammation in murine models.

The original submission data are marked (blue colored in the bracket), and supplementary experimental data are marked (green colored in the bracket), and revised parts are shown below (red colored in the text). The corresponding passages in the manuscript are marked in the same manner.

In "Results - Study on the cellular entry of cage/cargo protein complex" Section

The *in vivo* safety assessment encompassed acute toxicity screening (14-day observation), multi-organ

histopathology (H&E staining of liver, spleen, kidney, and heart; Extended Data Fig. 16A), serum biochemistry (ALT/AST for liver function, BUN/creatinine for kidney function; Extended Data Fig. 16B-C), and cytokine profiling (Extended Data Fig. 16D) - provides comprehensive evidence supporting the biosafety of pFn+216 at therapeutically relevant doses.

8. Immunogenicity concerns not addressed. High surface charge and unnatural residues may elicit immune responses. A brief discussion or preliminary data (e.g., cytokine assays) would improve confidence for therapeutic use.

Response:

To address this issue, we conducted comprehensive cytokine profiling in serum samples after intravenous administration of pFn+216 (Extended Data Fig. 16D). The data unequivocally demonstrates that key pro-inflammatory mediators - including IL-6, TNF-α, and IL-1β - remained at baseline levels indistinguishable from Saline-treated controls. This absence of cytokine elevation, coupled with histopathological evidence showing no immune cell infiltration in liver/kidney sections (Extended Data Fig. 16A), provides compelling evidence that the engineered nanocage maintains ferritin's innate immunological tolerance.

The original submission data are marked (blue colored in the bracket), and supplementary experimental data are marked (green colored in the bracket), and revised parts are shown below (red colored in the text). The corresponding passages in the manuscript are marked in the same manner.

In "Results - Study on the cellular entry of cage/cargo protein complex" Section

The *in vivo* safety assessment encompassed acute toxicity screening (14-day observation), multi-organ histopathology (H&E staining of liver, spleen, kidney, and heart; Extended Data Fig. 16A), serum biochemistry (ALT/AST for liver function, BUN/creatinine for kidney function; Extended Data Fig. 16B-C), and cytokine profiling (Extended Data Fig. 16D) - provides comprehensive evidence supporting the biosafety of pFn+216 at therapeutically relevant doses.

9. Please report loading efficiency (e.g., protein-to-cage ratio) and batch-to-batch variability if possible.

Response:

We have systematically optimized the loading parameters for pFn+216-based delivery through rigorous quantification of cargo binding efficiency. Flow cytometry analysis (Extended Data Fig. 10A) established a 1:8 molar ratio (pFn+216:GFP) as the optimal formulation, achieving near-saturation cellular uptake. We further validated the robustness of complex formation under physiologically relevant conditions: despite a 65% reduction in uptake efficiency in 100% serum (Extended Data Fig. 10E), the retained fluorescence intensity remained 30-fold above background, confirming stable complex integrity against serum protein interference. Furthermore, batch-to-batch consistency of the pFn+216@GFP nanocomplexes was also verified (Extended Data Fig. 11A-B).

The original submission data are marked (blue colored in the bracket), and supplementary experimental data are

marked (green colored in the bracket), and revised parts are shown below (red colored in the text). The corresponding passages in the manuscript are marked in the same manner.

In "Results - Study on the cellular entry of cage/cargo protein complex" Section

Furthermore, the stability of the pFn+216@GFP nanocomplexes across different production batches was validated. DLS analysis revealed nearly overlapping particle size distribution curves for three batches, with peak diameters consistently around 100 nm (Extended Data Fig. 11A), indicating batch-independent monodispersity of the complexes. Flow cytometry further confirmed functional consistency: GFP fluorescence intensity upon cellular uptake was similar across batches, with no significant differences in mean fluorescence intensity (Extended Data Fig. 11B).

10. Trim-Away experiments lack mechanistic controls. Consider adding an isotype antibody control and a catalytically inactive Trim21 mutant to demonstrate that degradation is target- and Trim21-dependent.

Response:

In accordance with your suggestion, we incorporated two critical controls: an isotype control antibody (IgG) and a catalytically inactive Trim21 RING-domain mutant (C16A) [1]. Neither control induced target protein degradation compared to the pFn+216@Trim21/anti-GFP group, confirming that both antigen-specific binding and the E3 ubiquitin ligase activity of Trim21 are essential for this process. (Extended Data Fig. 22).

The supplementary experimental data are marked (green colored in the bracket), and revised parts are shown below (red colored in the text). The corresponding passages in the manuscript are marked in the same manner.

Reference:

[1] Park, J. S. *et al.* Mechanical regulation of glycolysis via cytoskeleton architecture. *Nature* **578**, 621–626 (2020).

In "Results - Antibody-Mediated Degradation of Endogenous Proteins" Section

Furthermore, we included two additional critical controls: an isotype control antibody (IgG) and a catalytically inactive Trim21 RING-domain mutant (C16A)[35]. Neither control resulted in target protein degradation compared to the pFn+216@Trim21/anti-GFP group, confirming that both antigen-specific binding and functional Trim21 E3 ligase activity are essential for this process (Extended Data Fig. 22).

Minor Comments

11. Abstract: "good biosafety" is vague. Consider rephrasing.

Response:

We have replaced "good biosafety" with "excellent biocompatibility".

12. Title suggestion: Consider specifying the system and novelty, e.g., 'Supercharged Ferritin Nanocages Enable Universal Cytosolic Protein.'

Response:

Thank you for the suggestion. We have revised the title to "Supercharged ferritin nanocages enable universal cytosolic protein delivery."

13. Charge values should be defined more clearly. The meaning of Fn+216, Fn+312 etc., should be clarified earlier and consistently used.

Response:

We have implemented the following revisions in the "Results" section: a series of mutant Fn+ with different numbers of surface positive charge from Fn-168 to Fn+312 (net charges from -168 to +312) were expressed in *E. coli*.

14. Some sentences are overly long or vague. For example, 'a typical protein nanocage with unnatural amino acids'.

Response:

We sincerely appreciate the reviewer's meticulous attention to linguistic precision, which has significantly enhanced the clarity of our manuscript. In direct response to the comment regarding vague phrasing such as "a typical protein nanocage with unnatural amino acids", we have revised this statement to explicitly state: "By incorporating unnatural amino acids bearing phenylboronic acid groups, we developed a representative protein nanocage, pFn+. " We have also carefully reviewed and revised the remainder of the manuscript to ensure clarity and precision throughout.

15. Introduction could better differentiate from prior work.

Response:

Thank you for your suggestion. We have carefully revised the introduction to better contextualize our work in relation to previous studies, with emphasis on two key aspects: (1) While numerous efforts have been made to increase the net positive charge of peptides or proteins, surface charge limitations have restricted these systems to moderate levels (e.g., GFP+36 carrying a net charge of +36); (2) Conventional supercharged peptides or proteins exhibit a marked decline in delivery efficiency for large cargoes (>150 kDa), largely due to steric hindrance and lysosomal degradation. These limitations underscore the urgent need for new strategies that can overcome the current ceiling in achievable surface charge, systematically explore whether increasing positive charge enhances intracellular delivery, and address the persistent challenge of efficiently delivering high-molecular-weight protein cargos.

The revised parts are shown below (red colored in the text). The corresponding passages in the manuscript are

marked in the same manner.

In "Introduction" Section

Cationic CPPs, typically composed of 8-10 arginine or lysine residues, represent the most widely used and well-characterized class. To enhance their efficacy for delivering larger protein cargos, researchers have proposed various strategies - such as peptide cyclization[14] - which likely optimizes the spatial presentation of positive charges. The observation that CPPs tend to form clusters prior to internalization, combined with recent insights into the improved efficacy of cyclic and tricyclic CPPs, supports the hypothesis that higher surface-exposed positive charge correlates with increased delivery efficiency[2,15]. This is further corroborated by studies showing that the net charge of positively supercharged GFP variants strongly influences cellular uptake[16]. However, due to the limited number of surface-exposed amino acid residues in both CPPs and currently available supercharged proteins or polypeptides, achieving higher positive charge density remains challenging. This limitation fundamentally restricts their potential for efficient intracellular delivery. Notably, early supercharged protein systems generally operate within moderate net charge ranges (e.g., GFP+36[17]) and exhibit a critical bottleneck: delivery efficiency drops sharply for cargos larger than ~150 kDa, due to steric hindrance during internalization and inevitable sequestration in endolysosomal compartments[18]. To obtain a more impactful, universal, and biocompatible method for intracellular protein delivery, we aim to devise a novel approach that can overcome the current limitations.

16. Terminology clarification: Use consistent terminology for "cargo protein," "cargo," and "delivered protein" to avoid confusion.

Response:

In response to this valuable feedback, we have implemented comprehensive terminology standardization throughout the revised manuscript, supplementary materials, and figure legends. "Cargo protein" is now exclusively used to refer to all delivered macromolecules, replacing previous variations such as "cargo", "delivered protein"," or "protein cargoes".

17. Sequence verification of PBA incorporation: Mass spec or sequencing validation of the incorporation site is mentioned briefly; please provide more robust evidence.

Response:

We appreciate the reviewer's emphasis on rigorous validation of PBA incorporation. Our initial mass spectrometry analysis (Extended Data Fig. 7D-F) definitively confirmed site-specific modification: LC-MS/MS of tryptic digests identified a mass shift exclusively in the peptide spanning residues (HHHHHH*TTASTSQVR, where * denotes modified residue), with b/y ions unambiguously localizing the modification to Pro7 - consistent with 4-boronophenylalanine (4-BPA) modification. To further strengthen this evidence, we performed functional validation comparing unmodified Fn+216 and PBA-modified pFn+216. Crucially, only pFn+216 formed stable, monodisperse complexes with GFP (Extended Data Fig. 9A), and solely pFn+216 mediated efficient intracellular GFP delivery

(Extended Data Fig. 9B-D). This functional enhancement - directly attributable to PBA-enabled cargo binding - provides biological proof of successful incorporation. The convergence of high-resolution mass spectral data and dramatic functional consequences (cargo complexation and delivery exclusively in PBA-bearing variants) constitutes robust, multi-tiered evidence for site-specific PBA installation at position 7.

The original submission data are marked (blue colored in the bracket), and supplementary experimental data are marked (green colored in the bracket), and revised parts are shown below (red colored in the text). The corresponding passages in the manuscript are marked in the same manner.

In "Results - Screening of nanocage/cargo protein complex" Section

To determine whether PBA incorporation determines protein-binding capacity of Fn+216, we compared the cellular delivery efficiency of GFP mediated by Fn+216 versus pFn+216. PBA-modified Fn+216 formed stable, monodisperse complexes, with an average hydrodynamic diameter of approximately 100 nm (Extended Data Fig. 9A). Moreover, only pFn+216 successfully facilitated intracellular GFP delivery (Extended Data Fig. 9B-D), indicating that PBA modification is essential for enabling Fn+216 to functionally interact with cargo proteins.

Reviewer #4 (Remarks to the Author):

The work addresses a significant challenge in biotechnology—efficient protein delivery into mammalian cells—and proposes a novel platform. The manuscript examines the capabilities of this new platform. However, several concerns need to be addressed to improve the rigor of the claims.

Specific Comments:

Figure 1:

• The schematic is conceptually confusing. The transitions from Fn to Fn+ to pFn+ are presented in a manner that suggests sequential chemical steps, when they are rather strategic stages of design. Consider clarifying with arrows or descriptors.

Response:

We have added text descriptions to the arrows in the schematic diagram and provided more explanations in the figure captions.

The revised parts are shown below (red colored in the text). The corresponding passages in the manuscript are marked in the same manner.

In "Figure Legend" section of Fig. 1:

This platform utilizes a ferritin (Fn) nanocage, engineered through a two-step protein engineering strategy to achieve supercharged interfaces. The supercharged interface engineering strategy is achieved through a two-step protein engineering process. First, site-directed mutagenesis replaces solvent-exposed anionic residues (aspartic acid [D] and glutamic acid [E]) with cationic residues (lysine [K] or arginine [R]) at optimized positions on each ferritin monomer, yielding Fn+. Second, genetic code expansion (GCE) technology incorporates the unnatural amino acid 4-boronophenylalanine (4-BPA) at a defined surface site, appending phenylboronic acid (PBA) moieties, yielding pFn+. This pFn+ nanocage can be efficiently produced at scale in a cost-effective manner using an *E. coli* expression system. The platform enables the straightforward formation of complexes through simple mixing with various protein cargoes. These complexes are subsequently internalized by mammalian cells with high efficiency and released into the cytoplasm, where they exert their biological functions both *in vitro* and *in vivo*.

• The early presentation of "benefits" such as cost-effectiveness, biocompatibility, and efficient delivery appears premature and unsupported at this stage. It risks making the work seem promotional rather than hypothesis-driven.

Response:

Thank you for the suggestion. To ensure scientific rigor, we have removed all descriptive adjectives from the figure.

• The depiction of endosomal escape is both anatomically and mechanistically imprecise. Also, the placement of "in vitro" and "in vivo" labels inside the cell is unconventional and may mislead readers.

Response:

We have revised the schematic to more accurately depict the endosomal escape process and have removed the inappropriate "in vitro" and "in vivo" labels from Fig. 1.

• Protein cargos are shown as generic-colored ribbons. Using electrostatic potential-based coloring (e.g., red/blue for charge) would better support the narrative around surface charge and isoelectric point relevance.

Response:

In response to the reviewer's insightful suggestion, we have revised Fig. 1 and 3 to depict all protein cargoes using electrostatic potential-based coloring (red: negative charge; blue: positive charge; white: neutral). This modification significantly enhances the structural visualization.

Figure 2:

• The absence of pFn+ in the panel (despite its relevance to subsequent data) is a missed opportunity for continuity.

Response:

The purpose of Fig. 2 is to investigate the properties of supercharged Fn+ itself, specifically examining how progressively increasing surface charge influences cellular uptake efficiency and internalization pathways. Our results show a clear positive correlation between net charge and uptake efficiency, with no plateau observed even at a charge of +312 (Fig. 2C-D and Extended Data Fig. 5B-C). This finding provides valuable insights for the future development of next-generation supercharged protein platforms. In contrast, the introduction of PBA serves a distinct purpose: to enable cargo loading and delivery using Fn+ as a carrier. Thus, the two strategies address fundamentally different objectives.

• FITC signal interpretation is unclear: is it indicating endocytosis, surface binding, or cytosolic accumulation? Given FITC's pH sensitivity, endosomal versus cytoplasmic localization will significantly affect signal and interpretation.

Response:

The FITC signal in Fig. 2 quantitatively reflects effective cytosolic accumulation of delivered proteins. Although FITC fluorescence can be partially quenched in acidic endosomal compartments, it readily recovers upon escape into the neutral cytosolic environment. Moreover, as all FITC-labeled proteins were used at identical initial concentrations, differences in cytosolic fluorescence intensity should at least positively correlate with differences in cellular uptake efficiency. To enhance clarity, we have revised the description to explicitly refer to cytosolic protein accumulation.

In CD71^{Low} cells, increasing the surface charge of Fn^{+} variants correlate with enhanced cytosolic uptake efficiency (Fig. 2C and Extended Data Fig. 5B). Crucially, the CD71 blocking experiment (Fig. 2D and Extended Data Fig. 5C) demonstrates that above +168 charge, uptake efficiency becomes receptor-independent, confirming charge dominance over receptor-mediated endocytosis. Therefore, despite the pH sensitivity of FITC, the magnitude of fluorescence differences, together with mechanistic validation, provides compelling evidence for charge-enhanced cytosolic delivery.

The original submission data are marked (blue colored in the bracket), and revised parts are shown below (red colored in the text). The corresponding passages in the manuscript are marked in the same manner.

In "Results - Rational Design of supercharged protein nanocage" Section

Since wildtype Fn can target CD71 to enter cells[26], we further investigate the cytosolic accumulation of Fn+ using MCF10A (CD71^{Low}) and MDA-MB-231 (CD71^{High}) cells (Extended Data Fig. 5A).

• Particle size and aggregation state of the FITC-labeled constructs are not described at this stage of the paper, yet are essential to understanding uptake mechanisms.

Response:

To address this, we have now supplemented the manuscript with DLS analysis of FITC-labeled Fn+ nanocages. The data confirm that FITC conjugation does not compromise nanocage integrity: labeled Fn+ maintains a monodisperse size distribution identical to unlabeled Fn+ controls under physiological conditions (Extended Data Fig. 5B). This preservation of nanocage monodispersity and native size validates that subsequent uptake studies using FITC-Fn+ faithfully reflect the behavior of charge-dependent cellular internalization.

The supplementary experimental data are marked (green colored in the bracket), and revised parts are shown below (red colored in the text). The corresponding passages in the manuscript are marked in the same manner.

In "Results - Rational Design of supercharged protein nanocage" Section

Since wildtype Fn can target CD71 to enter cells[27], we further investigate the cytosolic accumulation of Fn+ using MCF10A (CD71^{Low}) and MDA-MB-231 (CD71^{High}) cells (Extended Data Fig. 5A). FITC conjugation did not compromise nanocage integrity, as labeled Fn+ maintained a monodisperse size distribution identical to unlabeled controls under physiological conditions (Extended Data Fig. 5B). In CD71^{Low} cells, there is a correlation between increased surface charge and enhanced cellular entry efficiency of Fn+ (Fig. 2C and Extended Data Fig. 5C).

• Use of transformed cancer lines (MCF and MDA) is limiting. Inclusion of primary cells would enhance translational relevance and better align with Figure 6.

Response:

To directly address concerns regarding delivery efficiency in primary cells, we conducted additional experiments using primary murine T lymphocytes and hepatocytes. Although primary cells are generally less permissive to protein carrier uptake, pFn+216 retained high delivery efficiency in both primary T cells and hepatocytes (Extended Data Fig. 12A). This broad applicability likely arises from its supercharged, charge-dominated uptake mechanism, which bypasses dependence on cell-type-specific surface receptors.

The supplementary experimental data are marked (green colored in the bracket), and revised parts are shown below (red colored in the text). The corresponding passages in the manuscript are marked in the same manner.

In "Results - Study on the cellular entry of cage/cargo protein complex" Section

This efficient delivery mediated by pFn+216 was remarkably consistent across multiple diverse cell lines (MDA-MB-231, C6, A549, PC12, BEL-7402, and Jurkat cells) and notably effective in transducing hard-to-transfect primary cells such as T lymphocytes and hepatocytes, highlighting its utility as a delivery vehicle (Extended Data Fig. 12A).

Figure 3:

• The amount of payload in particles is not quantified. Given the broad range of proteins tested, it is important to determine if loading is sequence- or charge-dependent.

Response:

We performed rigorous quantitative assessment of protein payload loading efficiency (Extended Data Fig. 10A), which established a universal 1:8 molar ratio (pFn+216:cargo) as the optimal formulation. This ratio achieves near-maximal cytosolic delivery efficiency while preserving complex stability, as evidenced by consistent complex sizes (102-108 nm) across a wide range of proteins with diverse charges and molecular weights (Extended Data Fig. 11 and 18). Subsequent *in vitro* and *in vivo* functional assays across multiple cargos further confirmed that this standardized ratio yields robust biological activity.

• Figure 3D would benefit from color-coding by protein cargo to assess any hidden trends.

Response:

As suggested, we investigated potential correlations between cargo protein properties (e.g., molecular weight, isoelectric point, hydrophobicity) and the hydrodynamic size of the resulting pFn+ - cargo complexes across different surface charge states. However, no statistically significant trends were observed. We speculate that this insensitivity arises from the extensive surface area of pFn+, combined with its surface charge density and reversible PBA-mediated conjugation mechanism (Fig. 3A, B), which together mitigate the impact of cargo-specific biophysical differences. Consistently, subsequent analyses of delivery efficiency and biological activity further highlight the platform's broad applicability across diverse protein cargos.

• The rationale for the behavior of highly negatively charged Fn variants is lacking—electrostatic repulsion would apply to negative charges as well. Additional discussion or data are needed to explain aggregation behavior.

Response:

Thank you for the valuable suggestion. We were also initially surprised by this result, but subsequent analysis suggests that this phenomenon likely arises from the interplay between surface charge heterogeneity and PBA-cargo affinity. For highly negatively charged Fn variants, two synergistic factors counteract the expected electrostatic repulsion: (1) Despite a net negative charge, the surface retains numerous lysine and arginine residues that form localized cationic patches, which can mediate electrostatic adhesion. In contrast, our strategy for introducing positive charge mutations involves replacing pre-existing acidic residues, progressively eliminating negative charges and yielding a surface dominated by uniformly distributed positive charges. As a result, increasing the number of positive mutations enhances electrostatic repulsion. (2) The PBA moieties exhibit strong binding affinity toward cargo proteins; however, the mixed-charge surface of negatively charged Fn - with coexisting positive and negative patches - fails to generate sufficient repulsive force to counteract PBA-mediated attraction, ultimately resulting in the formation of large aggregates (~1000 nm). Conversely, strongly cationic variants (≥ +240) possess a homogeneous surface charge landscape, where electrostatic repulsion dominates and effectively suppresses PBA-driven aggregation, yielding small assemblies (~10 nm). At intermediate charge states (+120 to +216), the balance between attractive and repulsive forces permits formation of ~100 nm nanocomplexes (Fig. 3B). Therefore, we have added a discussion in the Results section to describe the potential mechanisms underlying this phenomenon.

The original submission data are marked (blue colored in the bracket), and revised parts are shown below (red colored in the text). The corresponding passages in the manuscript are marked in the same manner.

In "Results - Screening of nanocage/cargo protein complex" Section

We speculate that two synergistic factors may override the anticipated electrostatic repulsion in highly negatively charged Fn variants: (1) Despite a net negative charge, the surface retains numerous lysine and arginine residues that form localized cationic patches, which can mediate electrostatic adhesion. In contrast, our strategy for introducing positive charge mutations involves replacing pre-existing acidic residues, progressively eliminating negative charges and yielding a surface dominated by uniformly distributed positive charges. As a result, increasing the number of positive mutations enhances electrostatic repulsion. (2) The PBA moieties exhibit strong binding affinity toward cargo proteins; however, the mixed-charge surface of negatively charged Fn - with coexisting positive and negative patches - fails to generate sufficient repulsive force to counteract PBA-mediated attraction, ultimately resulting in the formation of large aggregates (~1000 nm). Conversely, strongly cationic variants (≥ +240) possess a homogeneous surface charge landscape, where electrostatic repulsion dominates and effectively suppresses PBA-driven aggregation, yielding small assemblies (~10 nm). At intermediate charge states (+120 to +216), the balance between attractive and repulsive forces permits formation of ~100 nm nanocomplexes (Fig. 3B).

Figure 4:

• Panel A implies rapid delivery (within 1–8 min), yet other figures (e.g., 3) rely on 12-hour incubations. These

discrepancies should be reconciled or clarified.

Response:

The variation in incubation times across different experiments primarily reflect their distinct objectives. Flow cytometry data in Extended Data Fig. 10C demonstrate that cellular uptake of pFn+216 reaches a plateau at 12 hours, with substantial internalization already observed at 0.5 hours. Building on this, the previous Fig. 4A (now Extended Data Fig. 10D) employs single-molecule live-cell imaging to visualize the early-stage entry of pFn+216@GFP within the first 8 minutes. This provides intuitive visual evidence of rapid cellular entry, though it only captures the initial uptake phase. In contrast, the incubation times used in Fig. 5-7 is guided by the kinetics established in Extended Data Fig. 10C and are designed to assess the functional delivery and biological activity of protein cargos. These extended periods are necessary for downstream processes such as Cas9-mediated DNA cleavage to occur. To ensure greater rigor, we have carefully re-examined the entire manuscript and, wherever possible, specified the exact incubation times.

• Panel B's image quality is insufficient to convincingly determine intracellular localization. Live/dead cell indicators should be included to verify viability during imaging.

Response:

Thank you for the suggestion. We have replaced the original confocal images with quantitative flow cytometry data incorporating live/dead cell discrimination (revised Fig. 4B). Cells were co-stained with the viability dye 7-AAD, allowing gating exclusively on viable cell populations (Extended Data Fig. 10F), thereby ensuring that GFP fluorescence signals originate solely from metabolically active cells. The updated analysis demonstrates that pFn+216@GFP exhibits significantly higher delivery efficiency in live cells compared to PULSin@GFP.

The supplementary experimental data are marked (green colored in the bracket), and revised parts are shown below (red colored in the text). The corresponding passages in the manuscript are marked in the same manner.

In "Results - Study on the cellular entry of cage/cargo protein complex" Section

Our evaluation of pFn+216@GFP revealed significantly enhanced cellular uptake and intracellular localization compared to the classical protein transfection reagent PULSin@GFP (Fig. 4A). Flow cytometry further confirmed that pFn+216 efficiently delivers GFP into cells while preserving high cellular viability (Fig. 4B, Extended Data Fig. 10F).

• Panel C contains the ambiguity of what "cellular uptake" means, especially since FITC may report different compartments.

Response:

The term "cellular uptake" in Fig. 4C specifically refers to "Intracellular MFI" of functionally delivered cargo,

quantified by flow cytometry to eliminate compartment-specific artifacts. Therefore, the y-axis label in Fig. 4C should be revised to "Intracellular MFI Ratio (%)".

• Panel D contradicts Panel A, as it shows endosomal trapping at 10 min. Colocalization analysis is not quantitative and should not be used to conclude escape.

Response:

Thank you for the insightful comment. We have carefully re-examined and repeated the relevant experiments, consistently obtaining reproducible results. We believe the perceived discrepancies primarily stem from the distinct methodologies employed, without affecting the overall conclusion. The original Fig. 4A (now Extended Data Fig. 10D) utilizes high-speed live-cell imaging (5 frames per second), revealing rapid intracellular accumulation of green fluorescence within 60 seconds—supporting the notion of fast cellular entry. However, this experiment does not directly assess endosomal escape. In contrast, Fig. 4D employs confocal microscopy combined with lysosomal staining, providing qualitative evidence of lysosomal escape. Subsequent HiBiT assays (Fig. 4E-F) offer quantitative validation, demonstrating a total cytosolic delivery efficiency of 36.3%. Collectively, these complementary approaches offer a comprehensive view of the delivery process: Extended Data Fig. 10D captures rapid internalization, Fig. 4D qualitatively assesses endosomal escape, and Fig. 4E–F quantitatively confirm cytosolic delivery.

• The HiBiT system (Panel E–F) does support cytosolic delivery but there is insufficient evidence that this is resulting from endosomal escape.

Response:

While we acknowledge that the HiBiT system alone only confirms cytosolic accumulation of proteins, our collective experimental evidence strongly supports endosomal escape as the primary mechanism underlying cytosolic delivery. First, endocytic pathway analysis (Fig. 4C) demonstrated that uptake of pFn+216@cargo is energy-dependent, as it was completely inhibited at 4°C, thereby excluding the possibility of direct membrane translocation. The cargo primarily enters cells via caveolin-mediated and heparan sulfate proteoglycan-dependent pathways, which are known to traffic cargo into early endosomes [1-2]. Second, chloroquine treatment significantly enhanced pFn+216-mediated delivery (Extended Data Fig. 12C), further indicating endosomal involvement. Finally, confocal microscopy (Fig. 4D) revealed a diffuse cytoplasmic GFP signal within 1 hour of incubation, with only limited colocalization with the lysosomal marker LysoTracker, suggesting that a fraction of the delivered cargo successfully escaped the endosomal compartment. Together, the entry route analysis, chloroquine-enhanced delivery, LysoTracker colocalization, and quantitative HiBiT data collectively establish a coherent experimental framework that supports partial endosomal escape.

The original submission data are marked (blue colored in the bracket), and supplementary experimental data are marked (green colored in the bracket), and revised parts are shown below (red colored in the text). The corresponding

passages in the manuscript are marked in the same manner.

In “Results - Study on the cellular entry of cage/cargo protein complex” Section

The colocalization of the delivered GFP with lysotracker (lysosome marker) was studied. Colocalization analysis indicated that at 1 hours, GFP was diffusely distributed throughout the cytoplasm (Fig. 4D). In parallel, we employed the HiBiT-LgBit system to quantitatively evaluate lysosomal escape efficiency[30,31]. This method specifically measures cytoplasmic HiBiT peptide fluorescence relative to total cellular fluorescence (Fig. 4E). The results indicate that the lysosomal escape efficiency of pFn+216@HiBit (36.3%) is significantly higher than that of PULSin@HiBit (3.4%). Meanwhile, compared to PULSin, the total intracellular uptake and cytoplasmic quantity were approximately 2.0-fold and 20.7-fold higher, respectively (Fig. 4F). Chloroquine treatment significantly enhanced pFn+216-mediated delivery, further indicating endosomal involvement (Extended Data Fig. 12C). Collectively, these results indicate that pFn+216 facilitates efficient escape of its cargo from early endosomes prior to lysosomal degradation, leading to significant accumulation within the cytoplasm.

Reference:

[1] Sun F, Wang JZ, Luo JJ, Wang YQ, Pan Q. Exosomes in the Oncobiology, Diagnosis, and Therapy of Hepatic Carcinoma: A New Player of an Old Game. Biomed Res Int. 2018 Jul 25;2018:2747461.

[2] Mandarini E, Tollapi E, Zanchi M, Depau L, Pini A, Brunetti J, Bracci L, Falciani C. Endocytosis and Trafficking of Heparan Sulfate Proteoglycans in Triple-Negative Breast Cancer Cells Unraveled with a Polycationic Peptide. Int J Mol Sci. 2020 Nov 5;21(21):8282.

Figure 5:

• Panels A–B: It’s not established whether β-Gal or HRP are inactivated by endosomal entrapment. Therefore, the enzymatic readout cannot be definitively attributed to cytosolic activity. It could simply represent enhanced endocytic uptake.

Response:

While β-Gal and HRP can retain partial activity within endosomes, the observed signal enhancement with pFn+216 predominantly reflects superior cellular internalization efficiency rather than cytosolic-specific localization. To clarify this distinction, we have replaced “cytoplasmic delivery” with “intracellular”.

In “Results - Intracellular Delivery of Bioactive Proteins” Section

MDA-MB-231 cells treated with pFn+216@HRP exhibited significantly higher intracellular HRP activity compared to the control group.

• Panel D: Missing controls for toxicity of the delivery vehicle (pFn+ and PULSin) alone. It is not clear how the concentrations are determine and if the pFN+ or PULSin concentrations are changing as well (or just the payload).

Response:

Thank you for the thoughtful suggestion. As now shown in Fig. 5D-E, we included control groups treated with the delivery vehicle alone (pFn+216), which exhibited negligible cytotoxicity. The absence of toxicity from both free Saporin (or RNase) and pFn+216 confirms that the observed suppression of cell proliferation by pFn+216@Saporin (or pFn+216@RNase) arises from successful cytosolic delivery and biological activity of the cargo proteins. As noted in the figure legends, the amounts of pFn+216 and PULSin used were scaled proportionally with the cargo concentration to maintain a constant formulation ratio.

In "Figure Legend" section of Fig. 5D-E:

The molar ratio of pFn+216 to cargo proteins is 1:8, and the concentration of the pFn+216 control group is one-eighth that of the corresponding toxic protein.

• Panel F: The role of gRNA in Cas9 RNP formation and its impact on particle assembly should be experimentally verified. Cas9 alone is a poor proxy for RNP structure and charge.

Response:

We clarify that all structural and functional analyses in Fig. 5F-H and Extended Data Fig. 18-19 exclusively employed fully assembled Cas9 RNP complexes rather than Cas9 protein alone.

• Extended Datas Fig14: these cells don't look alive. If fixed, this raises concern of the fixation artifact.

Response:

Thank you for the suggestion. We repeated the experiment (Extended Data Fig. 19) and obtained consistent results. Experimental conditions were carefully controlled to minimize artifacts and ensure data reliability. A comparison between free RNP and PULSin@RNP supports the notion that pFn+216 is capable of delivering RNPs into the nucleus. Furthermore, both the cellular GFP knockdown assay (Fig. 5F) and the *in vivo* study (Fig. 6) confirm that RNPs delivered by pFn+216 remain functionally active within cells.

• 5I: cells are fixed. This has been reported to create artifacts in cell delivery studies: the laminal staining may happen post fixation. One could rationalize that this doesn't happen with PULSin. Nonetheless, this leaves the possibility that pFn+ are indeed destabilizing to endosomal escape….not enough for very much escape, but enough to contribute leakage during fixation. PULSin is potentially les active and does not give rise to the artifact. It is for instance puzzling that no endosomes are visible for pFn+, while they were present in Fig 4D

Response:

Fig. 5I-J collectively provide preliminary evidence that antibodies delivered by pFn+216 retain intracellular antigen-

binding activity. Flow cytometry (Fig. 5I; previously Fig. 5J) quantitatively confirms efficient internalization of the antibody, while confocal imaging (Fig. 5J; previously Fig. 5I) reveals characteristic accumulation at the nuclear envelope, consistent with the known localization pattern of anti–nuclear pore complex antibodies - suggesting preserved antigen recognition within the cell.

We fully acknowledge the reviewer's concern regarding potential fixation-induced artifacts. However, under identical fixation conditions, neither the free antibody nor the PULSin-treated group exhibited comparable nuclear envelope staining, suggesting that the observed pattern is more likely attributable to pFn+216-specific delivery rather than a general artifact introduced by fixation. That said, we recognize that the possibility of fixation-associated redistribution cannot be completely excluded. To address this, we further assessed antibody functionality under non-fixed, live-cell conditions. As shown in Fig. 7E-F, pFn+216-delivered antibodies induced degradation of the target protein in unfixed cells, thereby confirming successful cytosolic delivery and intracellular bioactivity. Collectively, these results support the conclusion that antibodies delivered by pFn+216 are functionally active inside cells, beyond what can be explained by fixation artifacts.

Figure 7:

• panel B. the microscopy data are qualitative and it is not clear what they provide (with risk of biased selection), given that C provides a quantitative measurement. Control data for pFn+ and PULSin alone are absent in both Panel C and Panel G, yet are essential to interpret specificity and toxicity.

Response:

As rightly noted, Fig. 7B is intended to provide an intuitive qualitative illustration, whereas Fig. 7C-D offer quantitative validation. We appreciate the reviewer's suggestion and, to exclude any potential contribution from the delivery vehicle itself, we have incorporated a control group treated with pFn+216 alone across all *in vitro* and *in vivo* experiments in Fig. 7. These controls consistently showed no detectable biological activity or toxicity, confirming that the observed effects are attributable solely to the functional cargo.

Additional Missing Information:

• If pFn+ successfully enters cells and escapes endosomes, what downstream effects does it have? Is there any stress response? The overall cytotoxicity is assessed, but cellular perturbation post-delivery (e.g., transcriptomic or proteomic signatures) would greatly improve confidence in biocompatibility.

Response:

We have conducted comprehensive transcriptomic profiling to evaluate potential cellular stress responses following pFn+216-mediated protein delivery (Extended Data Fig. 17). RNA-seq analysis comparing pFn+216-treated and vehicle control cells identified 118 significantly differentially expressed genes (DEGs), comprising 38 upregulated and 80 downregulated transcripts. Crucially, these DEGs showed no enrichment in canonical stress-response or cell death pathways (e.g., p53 signaling, apoptosis, ER stress, or oxidative phosphorylation). Gene Ontology (GO)

enrichment analysis revealed that the DEGs were predominantly associated with physiological regulatory processes including voltage-gated ion channel activity, cell-cell junction assembly, and basolateral plasma membrane organization - all indicative of homeostatic cellular adaptation rather than pathological perturbation. KEGG pathway analysis further confirmed exclusive enrichment in benign functional categories, most notably taste transduction, which has no established link to cytotoxicity. This molecular landscape, combined with our prior findings of >95% cell viability and absent inflammatory cytokine release, provides multidimensional evidence that pFn+216 delivery elicits minimal cellular perturbation at therapeutically relevant doses.

The supplementary experimental data are marked (green colored in the bracket), and revised parts are shown below (red colored in the text). The corresponding passages in the manuscript are marked in the same manner.

In "Results - Study on the cellular entry of cage/cargo protein complex" Section

To further evaluate potential cellular stress responses induced by pFn+216-mediated protein delivery, we conducted comprehensive transcriptomic profiling. RNA-seq analysis comparing pFn+216-treated and vehicle control cells identified 118 significantly differentially expressed genes (DEGs), comprising 38 upregulated and 80 downregulated transcripts (Extended Data Fig. 17A). Crucially, gene set enrichment analysis (GSEA) showed no enrichment in canonical stress-response or cell death pathways (e.g., p53 signaling, apoptosis, ER stress). Gene Ontology (GO) analysis indicated the DEGs were primarily associated with physiological regulatory processes, including voltage-gated ion channel activity, cell-cell junction assembly, and basolateral plasma membrane organization, consistent with homeostatic cellular adaptation (Extended Data Fig. 17B). KEGG analysis further confirmed enrichment solely in benign functional pathways, most notably taste transduction (Extended Data Fig. 17C).

• Figure 6: The in vivo gene editing data presented in Figure 6 show clear hepatic accumulation and functional editing in the liver, which is expected given the route of administration and nanoparticle size. While these results support the potential of pFn+216 for liver-targeted delivery, the impact is limited by the lack of exploration of delivery to other tissues or discussion of potential targeting strategies. The text could be strengthened by acknowledging this limitation and by clarifying whether liver delivery is an intended feature or a starting point for broader applications. A brief discussion of future directions, such as modifying the nanocage or changing administration routes to target other organs, would add important context.

Response:

Thank you very much for your insightful suggestion. This was an exploratory study, and we initially had no prior knowledge of where the complex would accumulate *in vivo*. The hepatic enrichment was identified empirically, and as you correctly noted, we believe it results from the administration route and the intrinsic size of the nanoparticle. While this may represent a limitation in some contexts, it also constitutes a defining feature of pFn+216. Its size favors selective hepatic uptake through physiological filtration mechanisms, aligning well with the unmet clinical needs of liver-targeted therapies. For instance, it enables efficient editing of hepatocyte-specific metabolic targets such as PCSK9 for hypercholesterolemia, while minimizing off-target exposure in non-hepatic tissues. In response, we have expanded the Discussion section, with a particular focus on the characteristics, limitations, and future

development directions of pFn+216.

The revised parts are shown below (red colored in the text). The corresponding passages in the manuscript are marked in the same manner.

In "Discussion" Section

In our *in vivo* gene editing studies, we observed predominant accumulation of pFn+216@cargo proteins complexes in the liver, which presents a substantial advantage for the development of therapeutics targeting liver-associated diseases. Nonetheless, expanding the targeting scope beyond the liver represents a key direction for future optimization. This could be achieved through fusion with tissue-specific homing peptides or small targeting moieties such as nanobodies or single-chain antibodies. Alternatively, surface charge masking strategies - for example, shielding positive charges with PEG - could enable conditional exposure of the supercharged surface in specific tissue microenvironments to achieve localized delivery. Moreover, the intrinsic cavity of Fn itself offers opportunities for cargo proteins loading[35] (e.g., metal ions or small molecules such as doxorubicin), enabling the development of multi-cargo co-delivery strategies that fully leverage the native properties of the Fn scaffold. Finally, while our supercharged interface engineering strategy has thus far been applied only to Fn, future studies may extend this approach to other natural or synthetic protein nanocages[36], providing valuable guidance for the design of next-generation intracellular delivery vehicles with tunable surface or exposed charge properties.

REVIEWER COMMENTS

Reviewer #1 (Remarks to the Author):

Although the authors have made substantial revisions in response to the reviewers' comments, several important concerns remain insufficiently addressed. We encourage the authors to revise the manuscript accordingly. Specific comments are as follows:

1. Figures 7E, 7F, 7L, S22D: Are these blots derived from a single experiment or from independent replicates? At least three biological replicates with appropriate statistical analyses are recommended.

Response:

Thank you for the suggestion. The Western blot results shown in Figures 7E, 7F, 7L, and S22D were obtained from three independent biological replicates, with each replicate representing a separate experiment. In the revised manuscript, we have performed appropriate statistical analyses on the quantitative data derived from these replicates.

2. The authors present data on pFn+216@RNP distribution across organs, but it remains unclear which cell types predominantly take up the complex—hepatocytes, Kupffer cells, hepatic stellate cells, or sinusoidal endothelial cells. This clarification is necessary.

Response:

We thank the reviewer for this important question. Our data indicate that pFn+216@RNP is taken up by multiple cell types in the liver, with the predominant uptake observed in Kupffer cells (KCs), the liver-resident macrophages. This is based on immunofluorescence analysis using the specific macrophage marker F4/80, which showed a strong signal co-localizing with the delivered complex (Fig. 6J). Furthermore, a minimal distribution was observed in endothelial cells and hepatocyte cells (Fig. 6H-I). The preferential accumulation in Kupffer cells suggests that our delivery system may be particularly advantageous for targeting macrophage-mediated liver pathologies. The revised parts are shown below (red colored in the text). The corresponding passages in the manuscript are marked in the same manner.

In Results "pFn+216@RNP complex effectively edits organs in vivo" Section

The pFn+216@RNP successfully targeted the SV40 polyA sequence, resulting in a marked increase in tdTomato fluorescence in the liver, indicative of successful gene editing (Fig. 6F-G). Immunofluorescence revealed tdTomato expression in multiple hepatic cell types - hepatocytes, endothelial cells, and Kupffer cells (Fig. 6H-J). Results showed predominant accumulation in Kupffer cells (Fig. 6J), with strong co-localization to the macrophage marker F4/80, consistent with their physiological role in filtering particulate matter from circulation. This Kupffer cell-preferential uptake suggests that pFn+216 is particularly suitable for targeting macrophage-associated liver disorders. Together, these results demonstrate that pFn+216 mediates efficient RNP delivery and functional gene editing in vivo, supporting its potential as a versatile platform for liver-directed therapies.

3. Figure 7I: Some tumors appear very small in volume. It would strengthen the findings to include an image of a dissected tumor to better illustrate anti-tumor efficacy. Since intratumoral injections were used, it is also important to examine how the complexes diffuse throughout the entire tumor. We recommend analyzing the intratumoral distribution of the complexes. Moreover, a group treated with Alectinib alone should be included to allow for evaluation of potential synergistic effects.

Response:

As recommended, to directly assess intratumoral distribution, we performed live imaging following intratumoral injection of Cy7-labeled complexes (Extended Data Fig. 24). Compared with the control groups (IgG-Cy7 alone and PULSin@IgG-Cy7), the pFn+216@IgG-Cy7 formulation exhibited markedly more uniform penetration and dispersion throughout the tumor mass, highlighting the superior distribution capability of our nanocage platform. In addition, we repeated the tumor-bearing mouse study with all groups starting from an average initial tumor volume of >100 mm^3. A new treatment arm with Alectinib monotherapy was included (Fig. 7H-N). Consistent with the resistant phenotype of the cancer cells used in our model, Alectinib alone did not elicit a significant antitumor response. The supplementary experimental data are marked (green colored in the bracket), and revised parts are shown below (red colored in the text). The corresponding passages in the manuscript are marked in the same manner.

In Results "Antibody-Mediated Degradation of Endogenous Proteins" Section

To evaluate the *in vivo* anti-tumor potential of this system, we performed real-time imaging after intratumoral injection of Cy7-labeled complexes. Compared to control groups (IgG-Cy7 alone and PULSin@IgG-Cy7), the pFn+216@IgG-Cy7 group exhibited significantly more uniform penetration and diffusion throughout the tumor tissue (Extended Data Fig. 24), demonstrating the superior distribution capability of our nanocage platform.

4. Figure 7M: Although necrosis and apoptosis were assessed, are these effects specifically attributable to pFn+216@Trim21/anti-ALK delivery? Dual or triple staining (e.g., TUNEL, Trim21/anti-ALK complex, and ALK) would help clarify potential colocalization.

Response:

To directly address whether the pro-apoptotic effects are specifically caused by the delivered cargo (Trim21/anti-ALK), we performed a complementary set of experiments. We conducted Annexin V/Propidium Iodide (AV/PI) flow cytometry analysis to quantitatively assess apoptosis and necrosis across all relevant control groups and pFn+216@Trim21/anti-ALK (Extended Data Fig. 23). The new results consistently demonstrate that a significant increase in apoptosis is observed exclusively in the group treated with the full pFn+216@Trim21/anti-ALK complex. The lack of a comparable effect in the groups containing the individual components or incomplete assemblies strongly confirms that the induction of apoptosis is a specific outcome of the successful intracellular delivery of the functional Trim21/anti-ALK complex, thereby directly attributing the effect to our delivery system. The supplementary experimental data are marked (green colored in the bracket), and revised parts are shown below (red colored in the text). The corresponding passages in the manuscript are marked in the same manner.

In Results "Antibody-Mediated Degradation of Endogenous Proteins" Section

To determine whether these effects specifically resulted from the intracellular delivery of functional cargo, we performed Annexin V/PI flow cytometry. Apoptosis was significantly enhanced only in cells treated with the complete pFn+216@Trim21/anti-ALK complex, confirming that the pro-apoptotic effect is cargo-specific and dependent on efficient nanocage-mediated delivery (Extended Data Fig. 23).

5. Figure 13: Please specify which cell types were used to evaluate uptake efficiency. In Figure 13A, if cell lysates were analyzed, appropriate internal or loading controls should be included.

Response:

We acknowledge an error in the original figure title for Extended Data Fig. 13. The correct title should be "Characterization of recombinant CPP-GFP fusion proteins." This figure panel was intended to present the biochemical characterization (specifically, SDS-PAGE, Western blot, and RP-HPLC analysis) of the purified fusion proteins prior to any cellular uptake experiments, and not a comparison of delivery efficiency. Therefore, it does not contain data from cell lysates. We apologize for the confusion caused by the incorrect labeling. The revised parts are shown below (red colored in the text). The corresponding passages in the manuscript are marked in the same manner.

In "Supplementary information" Figure Legend

Extended Data Fig. 13 Characterization of recombinant CPP-GFP fusion proteins.

All recombinant proteins, the series of cell-penetrating peptide (TP2, R9, DPV1047, PTD-4, Penetratin, dNP2, TAT, Pep-1, MPG, SCP) fused GFP constructs, were constructed, expressed, and purified in this study. The figure presents a comprehensive characterization to confirm the successful production, purity, and identity of these proteins. **A.** SDS-PAGE gel electrophoresis of a series of CPP-GFP proteins (10 ng/lane). A single, predominant band at the expected molecular size o is observed for each CPP-GFP fusion protein and the GFP control, demonstrating successful expression and high purity after purification. **B.** Recombinant proteins from (A) were immunoblotted using a primary antibody against GFP. A specific immunoreactive signal is detected for all samples, confirming that the purified proteins are the intended GFP-containing fusions. **C.** RP-HPLC analysis of a series of CPP-GFP proteins. Each sample displays a single, sharp major peak, indicating a homogeneous preparation. The distinct retention times for different constructs reflect their varying hydrophobicity profiles.

6. While the authors demonstrated typical CPP-GFP delivery, the important supercharged GFP positive control is missing (see PMID: 26465072, 29633093, and 35075948). This control should be included.

Response:

As recommended, we performed additional experiments incorporating supercharged GFP as a control. We compared the cellular delivery efficiencies of GFP, TAT-GFP (a classical CPP fusion), supercharged GFP+36, and our

pFn+216@GFP nanocage complex (Extended Data Fig. 8D-E). The results clearly show that pFn+216@GFP achieves the highest level of intracellular delivery, markedly outperforming both TAT-GFP and supercharged GFP+36. These findings further substantiate the superior performance of our supercharged nanocage-based delivery platform relative to existing supercharged protein benchmarks. The supplementary experimental data are marked (green colored in the bracket), and revised parts are shown below (red colored in the text). The corresponding passages in the manuscript are marked in the same manner.

In Results "Screening of nanocage/cargo protein complex" Section

Specifically, quantitative analysis of GFP fluorescence intensity in MDA-MB-231 and C6 cells showed that pFn+216@GFP achieved a markedly higher signal compared to GFP+36 (Extended Data Fig. 8D-E).

7. Figure S19: The authors claim that cytoplasm-released RNP enters the nucleus via nuclear localization sequences to mediate gene editing. How was this conclusion reached? Since no live-cell imaging was presented, we strongly recommend including real-time imaging to demonstrate whether cytoplasmic RNP complexes indeed enter the nucleus.

Response:

To address the question of nuclear entry, we performed a critical supplemental experiment using high-resolution confocal microscopy on fixed cells. We compared the subcellular distribution of our delivered RNP complexes with and without functional nuclear localization sequences (NLS). The results, now presented in the new Extended Data Fig. 19, clearly show a distinct pattern: RNP complexes conjugated with NLS peptides exhibit significant accumulation within the nucleus. In contrast, control RNP complexes without NLS remain predominantly localized in the cytoplasm. This stark difference in localization strongly supports the conclusion that the nuclear import of the RNP is an active, NLS-mediated process, rather than passive diffusion. The supplementary experimental data are marked (green colored in the bracket). The corresponding passages in the manuscript are marked in the same manner.

8. Materials and Methods: Additional details are required, including manufacturer information, catalog numbers, reagent concentrations, and staining protocols. Please also clarify the source of the RNPs (lab-made or commercial), whether they contain nuclear targeting sequences, and provide other critical methodological information that is currently missing.

Response:

We thank the reviewer for the helpful suggestion. In response, we have thoroughly revised the Materials and Methods section. Specifically, we have added the necessary experimental details and provided complete manufacturer information and catalog numbers for all commercial reagents. Both the Cas9 protein with a nuclear localization sequence and the version without it are commercially available products, and the relevant information has been indicated in the Materials and Methods section.

REVIEWER COMMENTS

Reviewer #1 (Remarks to the Author):

1. Although the authors claim that Kupffer cells predominantly take up pFn+216@RNP, as shown in Figure 6J, the quality of the three images (Fig. 6J–6J) is quite poor—particularly the DAPI signal in Figure 6J, which is barely visible. Moreover, the evidence provided by these three single staining images is insufficient to convincingly demonstrate that Kupffer cells are the major cell type responsible for particle uptake. Therefore, I recommend that the authors perform triple staining for CD31/HAS/F4/80 using confocal microscopy to clearly determine which liver cell type exhibits the highest uptake efficiency. Additionally, a flow cytometry–based uptake assessment of these three liver cell populations should be included to quantify their relative uptake percentages.

Response:

We sincerely thank you for your valuable comments regarding image quality and quantitative evaluation. We first re-stained a subset of the samples shown in Figures 6H-J to improve image quality and performed colocalization analysis, which revealed a strong overlap between the F4/80 and tdTomato signals. In addition, following your suggestion, we conducted supplementary flow cytometry-based experiments. After intravenous injection of Cy7-labeled pFn+216@RNP, primary liver cells were isolated at 24 hours post-injection and stained with antibodies against CD31 (endothelial cells), HSA (hepatocytes), and F4/80 (Kupffer cells). Quantitative analysis clearly demonstrated that, among the analyzed liver cell populations, the F4/80- and Cy7-double-positive cells accounted for the highest proportion (Extended Data Fig. 21). Collectively, these data confirm that Kupffer cells are indeed the predominant hepatic cell type responsible for the uptake of the pFn+216 nanocage. The supplementary experimental data are marked (green colored in the bracket), and revised parts are shown below (red colored in the text). The corresponding passages in the manuscript are marked in the same manner.

In Results "pFn+216@RNP complex effectively edits organs *in vivo*" Section

...*Ex vivo* imaging of major organs at 24 hours post-injection confirmed strong hepatic accumulation of fluorescence specifically in the pFn+216@RNP group (Fig. 6C-D), aligning with the *in vivo* data. To precisely identify the cellular tropism within the liver, we performed flow cytometric analysis of primary hepatocytes, Kupffer cells, and endothelial cells isolated from treated mice. This analysis revealed that Kupffer cells exhibited the highest uptake efficiency of pFn+216@RNP, with 41.0% of F4/80-positive cells showing high Cy7 signal, significantly surpassing the uptake by hepatocytes (8.98%) and endothelial cells (28.3%) (Extended Data Fig. 21)….

…Immunofluorescence revealed tdTomato expression in multiple hepatic cell types - hepatocytes, endothelial cells, and Kupffer cells (Fig. 6H-J). Quantitative colocalization analysis showed predominant accumulation in Kupffer cells, with strong overlap between F4/80 and tdTomato signals (Fig. 6J). This Kupffer cell-preferential uptake suggests that pFn+216 is particularly suitable for targeting macrophage-associated liver disorders….

2. Regarding the in vivo tumor model, the authors did not address whether the observed antitumor effect is dose dependent.

Response:

Following your suggestion, we performed additional in vivo experiments to systematically evaluate the dose-response relationship of the therapeutic effect. In the H3122/Ale subcutaneous tumor model, mice received intratumoral injections of pFn+216@Trim21/anti-ALK complexes every 3 days (with a molar ratio of 1:8:8 for pFn+216:Trim21:anti-ALK), with saline serving as the control. Doses were expressed as the equivalent amount of anti-ALK IgG per mouse: high (1 μg), medium (0.5 μg), and low (0.25 μg). The results revealed a pronounced dose-dependent antitumor effect, with tumor growth inhibition strongly correlated with increasing dosage. Specifically, the high-dose group exhibited the most substantial reduction in tumor volume, significantly outperforming both the medium- and low-dose groups (Extended Data Fig. 25). The supplementary experimental data are marked (green colored in the bracket), and revised parts are shown below (red colored in the text). The corresponding passages in the manuscript are marked in the same manner.

In Results "Antibody-Mediated Degradation of Endogenous Proteins" Section

To evaluate the *in vivo* anti-tumor potential of this system, we performed real-time imaging after intratumoral injection of Cy7-labeled complexes. Compared to control groups (IgG-Cy7 alone and PULSin@IgG-Cy7), the pFn+216@IgG-Cy7 group exhibited significantly more uniform penetration and diffusion throughout the tumor tissue (Extended Data Fig. 24), demonstrating the superior distribution capability of our nanocage platform. We further investigated the dose-dependent therapeutic efficacy. Using the H3122/Ale subcutaneous tumor model, intratumoral injections of pFn+216@Trim21/anti-ALK complexes at three distinct doses (low, 0.25 μg; medium, 0.5 μg; and high, 1.0 μg anti-ALK IgG per mouse) revealed a clear dose-dependent inhibition of tumor growth, with the high-dose group showing the most potent effect and no associated systemic toxicity (Extended Data Fig. 25). Subsequently, we assessed the comparative in vivo anti-tumor efficacy of pFn+216@Trim21/anti-ALK (at the high dose) in H3122/Ale tumor-bearing mice via intratumoral injection (Fig. 7H). Tumor growth inhibition was significantly greater in the pFn+216@Trim21/anti-ALK group compared to the PULSin@Trim21/anti-ALK, Trim21/anti-ALK alone, pFn+216 alone, Alectinib alone and saline control groups (Fig. 7I, J).

3. In Figures 7I and 7J, Alectinib shows almost no effect on tumor size. However, Figure S6 in the referenced preprint (doi: 10.1101/2024.04.24.590626) clearly demonstrates that Alectinib (20 mg/kg/day) reduces H3122 tumor growth after 4–6 days of oral administration. The discrepancy between the present results and previously reported findings requires a thorough explanation, as it raises concerns about whether any procedures or experimental conditions in the current study may not have been adequately designed or controlled.

Response:

We thank you for your careful observation regarding the discrepancy in the efficacy of Alectinib between our study and previous reports. The observed discrepancy arises from fundamental differences in the cellular models employed. In the present study, we used the H3122/Ale cell line, an Alectinib-resistant subline derived from parental H3122 cells, which was established to model clinically acquired resistance. This point was already explicitly stated in the

Results section of the previous version of the manuscript: "EML4-ALK, a well-characterized oncogene, encodes a cytoplasmic fusion protein that drives tumor progression[36]. Resistance to small-molecule ALK inhibitors often leads to treatment failure[37], highlighting the need for alternative strategies such as targeted protein degradation. To test this hypothesis, we employed an Alectinib-resistant H3122 cell line (H3122/Ale)." The detailed procedure for generating this resistant subline has been described in our earlier publication (doi: 10.1111/bph.15801). Consistent with its resistant phenotype, treatment with Alectinib alone (20 mg/kg/day) did not result in significant tumor growth inhibition in our model, further confirming the insensitivity of this cell line to Alectinib. Therefore, the results observed in the control group of the present study do not contradict the data reported in the cited preprint (doi: 10.1101/2024.04.24.590626). The revised parts are shown below (red colored in the text). The corresponding passages in the manuscript are marked in the same manner.

In Methods "In vivo mouse models and treatments" Section

Mice were euthanized when the tumor burden exceeded the threshold. The establishment method of H3122/Ale cell line, as detailed in our other published work[40], was designed to mimic clinically common acquired resistance. For H3122/Ale subcutaneous tumor model, female mice (6-8 weeks) were inoculated with H3122/Ale cells (1×10^6) in the right inguinal region.

4. In the Methods section, antibody dilutions should be clearly stated, and the exact amounts of proteins or complexes used remain missing. The number of mice per group, as well as their sex and age, should also be provided. Finally, full statistical analyses must be reported for all figures, including details of multiple comparison methods where applicable.

Response:

In response to these concerns, we have comprehensively revised the Methods and Figure Legend sections and added the requested experimental details in accordance with your suggestions. Specifically, we now clearly state the dilution ratios of all antibodies used (e.g., anti-HSA at 1:1000, anti-CD31 at 1:200, and anti-F4/80 at 1:500). In addition, the exact amounts or ratios of proteins or complexes used in both in vitro and in vivo experiments are now explicitly specified. For the animal studies, we have provided complete information regarding the number of mice per group, as well as their sex and age. Finally, full statistical analyses have been added for all figures. Data are presented as mean ± SEM, and all statistical details, including exact p-values and the number of replicates (n), are now clearly indicated in the corresponding figure legends. The revised parts are shown below (red colored in the text). The corresponding passages in the manuscript are marked in the same manner.

Thank you !

REVIEWER COMMENTS

Reviewer #1 (Remarks to the Author):

1. In line 747, the authors cite reference 40 as the protocol for establishing the H3122/Ale cell line. However, the referenced study used MCF7, MDA-MB-231, and HCT8 cells, none of which are related to the H3122/Ale cell line. Therefore, it remains unclear how the H3122/Ale cell line was generated in this study. Given the well-known issue of cell line misidentification and contamination in the field, the authors should provide cell line authentication data, such as STR profiling or whole-genome sequencing (WGS), to confirm the identity of the H3122/Ale cells.

Response：

Following the established principle of reference 40, the H3122/Ale cell line in our study was generated specifically by chronically exposing the parental H3122 cell line to increasing concentrations of Alectinib until a stably resistant population was obtained. In response to your suggestion, we performed STR profiling to authenticate the identity of the newly established H3122/Ale cell line. A screenshot of the STR report is provided below for reference. This authentication ensures the validity of our model for studying resistance mechanisms.

Report of Human Cell Line Authentication

I. Sample

Sample Name: labeled as 'NCI-H3122'.

II. Method and Procedure

1. PCR is amplified with STR Multi-Amplification Kit (PowerPlex 21D System);
2. PCR products are assayed with 3100 DNA Analyzer (Applied Biosystems®).
3. Amplification of gene COI and electrophoresis are employed to survey the species of the sample.

III. Results

1. The STR profiles of the cell line sample are in the attached table and figure.
2. The search result in DSMZ and ExPASy databases.
3. The electrophoresis figure of gene COI.

NCI-H3122: ①No loci has tri-alleles or tetra-alleles. Contamination of other human cell lines are not found (Figure 1 & Table 1). ②Compared the STR data of NCI-H3122 cell line in the databases of DSMZ and ExPASy, the alleles of NCI-H3122 were 91.3% matched with the alleles of NCI-H3122 cells found in ExPASy cell bank (Figure 2&3). ③The sample is a human cell line. Contamination of other species cells are not found in the sample (Figure 4).

To all above, the sample is a single cell line, and it is derived from a common ancestry with NCI-H3122 cell line.

Operator: Wanting Jiang

Auditor: Ting Xu

Guangzhou Cellcook Biotech Co., Ltd

GUANGZHOU CELLCOOK BIOTECH CO.,LTD 30 Jan 2026 CELL STR PROFILING PASS

(Notice: This authentication report is restricted to the cell sold from Guangzhou Cellcook Biotech Co., Ltd, and the date with seal is the date of delivery.)

Figure 1. STR profiles of NCI-H3122 cell line

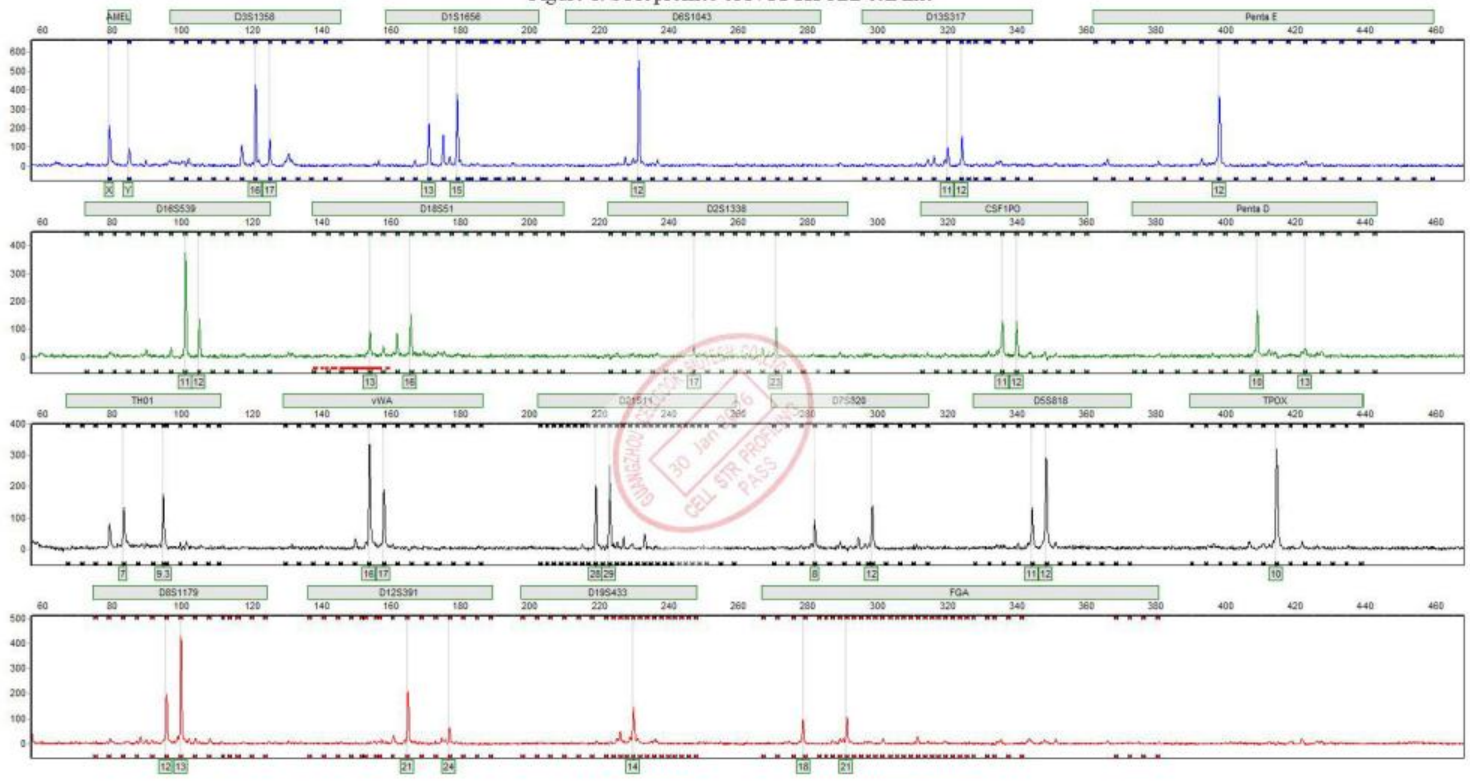

Table 1. STR profiles of NCI-H3122 cell line

21	Allele1	Allele2
AMEL	X	Y
D3S1358	16	17
D1S1656	13	15
D6S1043	12	
D13S317	11	12
Penta E	12	
D16S539	11	12
D18S51	13	16
D2S1338	17	23
CSF1PO	11	12
Penta D	10	13
TH01	7	9.3
vWA	16	17
D21S11	28	29
D7S820	8	12
D5S818	11	12
TPOX	10	
D8S1179	12	13
D12S391	21	24
D19S433	14	
FGA	18	21

Figure 2. Search result in DSMZ database

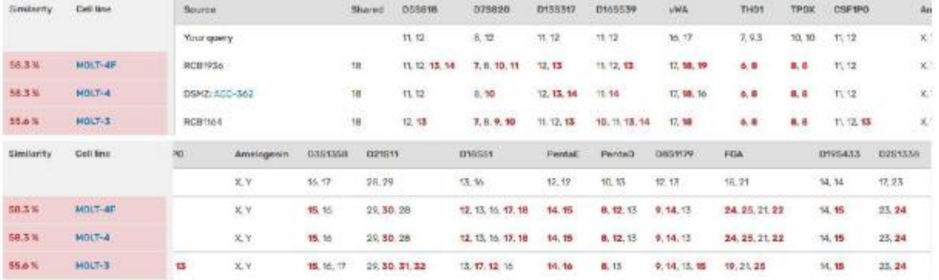

Similarity	Cell line	Source	Shared	D5S818	D7S820	D13S317	D16S539	vWA	TH01	TPOX	CSF1PO	Am[illegible]
		Your query		11, 12	8, 12	11, 12	11, 12	16, 17	7, 9.3	10, 10	11, 12	X, [illegible]
58.3 %	MOLT-4F	RCB1936	18	11, 12, 13, 14	7, 8, 10, 11	12, 13	11, 12, 13	17, 18, 19	6, 8	8, 8	11, 12	X, [illegible]
58.3 %	MOLT-4	DSMZ: ACC-362	18	11, 12	8, 10	12, 13, 14	11, 14	17, 18, 16	6, 8	8, 8	11, 12	X, [illegible]
55.6 %	MOLT-3	RCB1164	18	12, 13	7, 8, 9, 10	11, 12, 13	10, 11, 13, 14	17, 18	6, 8	8, 8	11, 12, 13	X, [illegible]

Similarity	Cell line	[illegible]PO	Amelogenin	D3S1358	D21S11	D18S51	PentaE	PentaD	D8S1179	FGA	D19S433	D2S1338
			X, Y	16, 17	28, 29	13, 16	12, 12	10, 13	12, 13	18, 21	14, 14	17, 23
58.3 %	MOLT-4F		X, Y	15, 16	29, 30, 28	12, 13, 16, 17, 18	14, 15	8, 12, 13	9, 14, 13	24, 25, 21, 22	14, 15	23, 24
58.3 %	MOLT-4		X, Y	15, 16	29, 30, 28	12, 13, 16, 17, 18	14, 15	8, 12, 13	9, 14, 13	24, 25, 21, 22	14, 15	23, 24
55.6 %	MOLT-3	13	X, Y	15, 16, 17	29, 30, 31, 32	13, 17, 12, 16	14, 16	8, 13	9, 14, 13, 15	19, 21, 25	14, 15	23, 24

Figure 3. Search result in ExPASy database

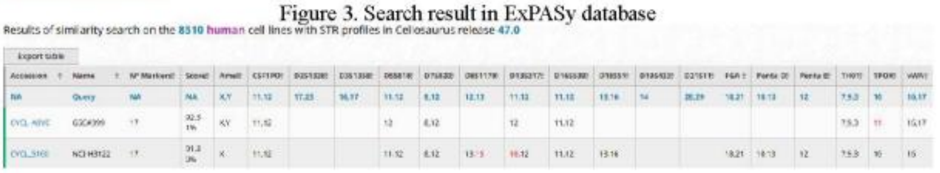

Results of similarity search on the 8510 human cell lines with STR profiles in Cellosaurus release 47.0

Export table

Accession	Name	Nº Markers	Score	Amel	CSF1PO	D2S1338	D3S1358	D5S818	D7S820	D8S1179	D13S317	D16S539	D18S51	D19S433	D21S11	FGA	Penta D	Penta E	TH01	TPOX	vWA
NA	Query	NA	NA	X,Y	11,12	17,23	16,17	11,12	8,12	12,13	[illegible]	11,12	13,16	14	28,29	18,21	10,13	12	7,9.3	10	16,17
CVCL_[illegible]	[illegible]	17	92.51%	X,Y	11,12			12	8,12		12	11,12							7,9.3	11	16,17
CVCL_[illegible]	NCI-H3122	17	91.2[illegible]%	X	11,12			11,12	8,12	13,[illegible]	[illegible],12	11,12	13,16			18,21	10,13	12	7,9.3	10	[illegible]

Figure 4. Authentication of the species of the sample

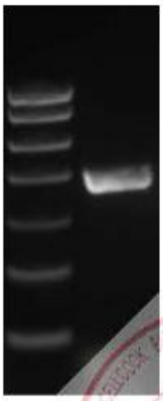

M: Marker. As the size of 700, 600, 500, 400, 300, 200 and 100bp from up to down.

Eight species are checked, as follow: *Homo sapiens* 391bp, *Cricetulus griseus* 315bp, *Macaca mulatta* 287bp, *Cercopithecus aethiops* 222bp, *Rattus norvegicus* 196bp, *Canis familiaris* 172bp, *Mus musculus* 150bp, *Bos Taurus* 102bp.

The sample: The band size is 391bp which matches the size of human.

2. Several published studies (PMID: 39904499, PMID: 40234387, and PMID: 32041944) have clearly demonstrated, both in vitro and in vivo, that alectinib effectively inhibits the growth of H3122 tumor cells. In contrast, the present manuscript reports that alectinib has no effect on the growth of the H3122 cell line. The authors should clarify this discrepancy. If alectinib truly has no effect, it is also unclear why the mice were treated with alectinib for nearly one month. Moreover, Figure S23 clearly shows that alectinib significantly induces apoptosis in H3122/Ale cells, which further contradicts the stated lack of efficacy.

Response:

The H3122 subline used in this study is an alectinib-resistant derivative (H3122/Ale), which has been clearly indicated in both the Methods and Results sections. This resistant cell line was established through long-term in vitro exposure of parental H3122 cells to gradually increasing concentrations of alectinib, following commonly used approaches as cited in the manuscript. The generation of drug-resistant cell lines using this strategy has been extensively described across multiple cell types and in numerous published studies [1-3].

Because the studies you cited did not employ an alectinib-resistant cell line, alectinib understandably exhibited significant antitumor effects in those reports. Therefore, their findings are

not contradictory to ours. The primary focus of our study is the development of an efficient intracellular protein delivery system rather than the development of a novel antitumor therapy. As such, the description of the resistant cell line establishment may have been relatively brief in the original manuscript. To further address your concern, we performed comprehensive validation of the resistant phenotype of H3122/Ale cells: Western blot analysis confirmed upregulation of the efflux transporters ABCB1 and ABCC1 (Extended Data Fig. 23A); CCK-8 assays showed a 16.2-fold increase in the IC_{50} value compared with parental H3122 cells (Extended Data Fig. 23B); and clonogenic assays together with flow cytometric apoptosis analysis (Extended Data Fig. 23C-F) demonstrated that while parental H3122 cells exhibited significant dose-dependent inhibition at 1, 10, and 100 nM alectinib, H3122/Ale cells showed a markedly attenuated response, with significant effects observed only at the highest concentration (100 nM).

Therefore, the term "lack of efficacy" in our manuscript specifically refers to the substantially reduced or lost responsiveness in this resistant model. In the in vivo experiments, alectinib served as a control treatment to highlight that our therapeutic strategy remains effective against alectinib-resistant tumors. As noted in the Results section, "Resistance to small-molecule ALK inhibitors often leads to treatment failure." In non-resistant tumors, the advantage of protein-targeted degradation technology would not be fully demonstrated. For this reason, we established the alectinib-resistant H3122 cell line.

The supplementary experimental data are marked (green colored in the bracket), and revised parts are shown below (red colored in the text). The corresponding passages in the manuscript are marked in the same manner.

Reference:

[1] Meng X, Zhao Y, Han B, Zha C, Zhang Y, Li Z, Wu P, Qi T, Jiang C, Liu Y, Cai J. Dual functionalized brain-targeting nanoinhibitors restrain temozolomide-resistant glioma via attenuating EGFR and MET signaling pathways. Nat Commun. 2020 Jan 30;11(1):594.

[2] Zhou X, Chen R, Yu Z, Li R, Li J, Zhao X, Song S, Liu J, Huang G. Dichloroacetate restores drug sensitivity in paclitaxel-resistant cells by inducing citric acid accumulation. Mol Cancer. 2015 Mar 19;14:63.

[3] Huang L, Perrault C, Coelho-Martins J, Hu C, Dulong C, Varna M, Liu J, Jin J, Soria C, Cazin

L, Janin A, Li H, Varin R, Lu H. Induction of acquired drug resistance in endothelial cells and its involvement in anticancer therapy. J Hematol Oncol. 2013 Jul 9;6:49.

In Results "Antibody-Mediated Degradation of Endogenous Proteins" Section

……To test this hypothesis, we established the resistant H3122/Ale subline via chronic, escalating Alectinib exposure. Compared to the parental cells, H3122/Ale exhibited upregulation of the efflux transporters ABCB1 and ABCC1 (Extended Data Fig. 23A) and an approximately 16.2-fold increase in Alectinib resistance (Extended Data Fig. 23B). Functional assays confirmed a markedly attenuated response: while parental H3122 cells showed potent, dose-dependent inhibition at low Alectinib concentrations (1-100 nM), significant effects in H3122/Ale cells were only observed at the highest dose (100 nM) (Extended Data Fig. 23C-F). Critically, western blot analysis confirmed that pFn+216@Trim21/αALK significantly reduced EML4-ALK levels in H3122/Ale cells (Fig. 7F) and markedly inhibited cell proliferation (Fig. 7G).……

3. In Extended Data Fig. 25, although tumor volume was measured in H3122-bearing mice, it remains unknown whether the observed antitumor effect is dependent on EML4-ALK degradation.

Response:

To directly address whether the observed antitumor effect is dependent on EML4-ALK degradation, we performed a subsequent Western blot analysis on tumor tissues. The results, now included in the revised manuscript, demonstrate a clear dose-dependent reduction in EML4-ALK protein levels following treatment with pFn+216@Trim21/anti-ALK (Extended Data Fig. 26E-F). These findings establish a strong mechanistic association between EML4-ALK degradation and tumor suppression. The supplementary experimental data are marked (green colored in the bracket), and revised parts are shown below (red colored in the text). The corresponding passages in the manuscript are marked in the same manner.

In Results "Antibody-Mediated Degradation of Endogenous Proteins" Section

……Using the H3122/Ale subcutaneous tumor model, intratumoral injections of pFn+216@Trim21/αALK complexes at three distinct doses (low, 0.25 μg; medium, 0.5 μg; and

high, 1.0 μg αALK IgG per mouse) revealed a clear dose-dependent inhibition of tumor growth, with the high-dose group showing the most potent effect and no associated systemic toxicity (Extended Data Fig. 26A-D). Further analysis of tumor lysates revealed a dose-dependent downregulation of EML4-ALK, phosphorylated AKT (p-AKT), and the anti-apoptotic protein BCL-2, with the most pronounced reduction observed in the high-dose group (Extended Data Fig. 26E-F). Finally……

4. In Figure 7, based on the presented data, it is still unclear whether pFn+216 can induce cell death in the presence of alectinib co-administration.

Response:

The primary objective of the experiment presented in Figure 7 was to evaluate the ability of the pFn+216 platform to deliver the Trim21/anti-ALK complex into cells, thereby highlighting the capacity of our intracellular protein delivery system to transport large proteins or protein complexes. The alectinib-resistant cell line was selected because resistance to ALK inhibitors is a well-recognized clinical challenge. In principle, targeted protein degradation, which directly eliminates the disease-driving protein, is expected to remain effective even in resistant cells. Evaluating the strategy in a resistant model therefore better highlights the intrinsic advantages of protein-targeted degradation - an approach widely acknowledged in the field. This experimental design is related to the nature of the cargo being delivered rather than to the delivery system itself.

Compared with the alectinib-only and control groups, the group treated with the pFn+216@Trim21/anti-ALK complex exhibited significant tumor regression and increased apoptosis. These results demonstrate that our delivery system is capable of transporting functional protein complexes into cells and that the delivered complexes retain their biological activity intracellularly.

5. Although the authors examined total EML4-ALK levels, key downstream signaling pathways were not adequately assessed. Specifically, the status of pALK/ALK, pERK/ERK, pAKT/AKT, and pS6/S6 remains unknown, despite these signaling axes being critical regulators of H3122 cell growth and survival.

Response:

We further performed additional Western blot analyses to systematically evaluate the signaling changes following EML4-ALK degradation. The results confirm that successful knockdown of EML4-ALK by pFn+216@Trim21/anti-ALK markedly suppressed the activity of its critical downstream signaling axis. Specifically, we observed a significant decrease in AKT phosphorylation (p-AKT), accompanied by a pronounced reduction in the protein level of BCL-2, a key anti-apoptotic effector regulated by the AKT signaling pathway (Extended Data Fig. 26E-F). The supplementary experimental data are marked (green colored in the bracket), and revised parts are shown below (red colored in the text). The corresponding passages in the manuscript are marked in the same manner.

In Results "Antibody-Mediated Degradation of Endogenous Proteins" Section

……Using the H3122/Ale subcutaneous tumor model, intratumoral injections of pFn+216@Trim21/αALK complexes at three distinct doses (low, 0.25 µg; medium, 0.5 µg; and high, 1.0 µg αALK IgG per mouse) revealed a clear dose-dependent inhibition of tumor growth, with the high-dose group showing the most potent effect and no associated systemic toxicity (Extended Data Fig. 26A-D). Further analysis of tumor lysates revealed a dose-dependent downregulation of EML4-ALK, phosphorylated AKT (p-AKT), and the anti-apoptotic protein BCL-2, with the most pronounced reduction observed in the high-dose group (Extended Data Fig. 26E-F). Finally,……

<u>6. Given the substantial difference in antitumor efficacy between the pFn+216@Trim21/αALK group and the alectinib group in the mouse model, it is possible that non-ALK-degradation mechanisms contribute to tumor growth inhibition. To address this possibility, administering MG132 (or vehicle control) after pFn+216@Trim21/αALK treatment would help determine whether the observed tumor suppression is indeed ALK-degradation dependent.</u>

Response:

First, we would like to clarify that the difference in antitumor efficacy between the

pFn+216@Trim21/αALK group and the alectinib group is attributable to the use of an alectinib-resistant cell line. As detailed in our previous response, we employed a resistant model specifically to address the clinical challenge of ALK inhibitor resistance and have already explained the rationale for this choice. During the revision process, we also incorporated sufficient additional controls, including a blank pFn+216 control group, multiple dosage groups, and extensive analyses of relevant downstream signaling pathways. Collectively, these data adequately demonstrate that the observed antitumor effect is not due to the pFn+216 carrier itself, which is the central focus of our study.

We sincerely appreciate your constructive suggestions, which have helped improve the quality of our manuscript. In accordance with your recommendation, we performed a rescue experiment using the proteasome inhibitor MG132. A comprehensive set of assays - including CCK-8 cell viability, clonogenic formation, and Western blot analysis of ALK protein levels - consistently showed that MG132 significantly attenuated the antitumor effect of pFn+216@Trim21/αALK (Extended Data Fig. 24). These findings indicate that the pronounced tumor-suppressive effect of pFn+216@Trim21/αALK is mechanistically dependent on the proteasomal degradation of the EML4-ALK oncoprotein.

The supplementary experimental data are marked (green colored in the bracket), and revised parts are shown below (red colored in the text). The corresponding passages in the manuscript are marked in the same manner.

In Results "Antibody-Mediated Degradation of Endogenous Proteins" Section

……To further demonstrate that the anti-tumor effect depends on proteasomal degradation of the target protein, we employed the proteasome inhibitor MG132. Co-treatment with MG132 effectively reversed the key outcomes induced by pFn+216@Trim21/αALK: it prevented EML4-ALK degradation (Extended Data Fig. 24A), restored cell viability in CCK-8 assays (Extended Data Fig. 24B), and largely rescued clonogenic survival (Extended Data Fig. 24C, D).

<u>7. In addition, the authors have not clearly labeled the statistical analysis methods or comparison strategies used in each relevant figure.</u>

Response:

We have now thoroughly revised the manuscript to explicitly state the statistical methods and comparison strategies for all relevant experiments. The "Statistical Analysis" subsection in the Methods section has been revised to provide detailed explanations of the specific testing methods used. Furthermore, each figure legend now explicitly specifies the statistical test applied, the exact value of n for each experiment, and the meaning of all error bars. All significant comparisons are clearly indicated in the figures, and the specific pairwise comparisons performed are described in the respective legends. The revised parts are shown below (red colored in the text). The corresponding passages in the manuscript are marked in the same manner.

In Methods "Statistical Data Analysis" Section

Data are presented as the mean ± SEM. GraphPad Prism 10.5 (GraphPad Software Inc.) was used for statistical analyses. The declared group size (n) refers to the number of independent values rather than technical replicates; technical replicates were averaged to generate a single independent value. Statistical comparisons between two groups were performed using an unpaired two-tailed Student's t-test, and multi-group comparisons were analyzed using one-way or two-way ANOVA followed by Tukey's or Šídák's multiple comparisons test. No outliers were excluded from the analyses. $^{*}P<0.05$, $^{**}P<0.01$, and $^{***}P<0.001$ were considered statistically significant.

8. Finally, in Extended Data Fig. 21, a proper negative control group is missing. Moreover, the percentage of tdTomato-positive cells within the Cy7-positive population should also be quantified and reported.

Response:

Thank you for your suggestion. We have added a negative control group, RNP-Cy7 (i.e., the formulation without the pFn+216 nanocage carrier). As expected, virtually no Cy7 signal was detected in any of the analyzed liver cell populations, clearly highlighting the critical role of pFn+216 in efficient cellular delivery.

Regarding the quantification of tdTomato-positive cells, we would like to clarify the primary purpose of Extended Data Fig. 21. This experiment was specifically designed to determine cellular

tropism - that is, which liver cell types take up the nanocage - using the Cy7 label as a direct tracer. The sgRNA used in this experiment was a non-targeting control sequence with no cleavage activity; therefore, it cannot activate tdTomato expression, making tdTomato quantification irrelevant for this analysis. The observed fluorescence signal is entirely derived from the Cy7 label.

Thank you!

REVIEWER COMMENTS

Reviewer #1 (Remarks to the Author):

1. The authors state that alectinib-resistant H3122/Ale cells were used to generate the alectinib-resistant H3122 tumor model. However, according to the Methods, no alectinib was administered before the tumors reached 100 mm³, so it is unclear how alectinib resistance was maintained in vivo during this period. In addition, it is not justified why 20 mg/kg/day alectinib is sufficient to maintain alectinib resistance in mice. The authors should provide a clear rationale (with references and/or pilot data) for this dosing and explain how resistance was verified and preserved in the animal model.

Response:

We thank the reviewer for this important question regarding the maintenance of the drug-resistant phenotype in our in vivo model. The alectinib-resistant phenotype of H3122/Ale cells was established through long-term, stepwise drug selection in vitro. During in vitro culture, continuous drug exposure is typically required to maintain selective pressure and prevent the outgrowth of drug-sensitive subpopulations. However, in in vivo xenograft studies, it is common practice to implant pre-established drug-resistant cells without continuous drug administration during tumor establishment [1-2]. Under these conditions, resistant CDX and PDX models are widely used and generally retain their drug-resistant phenotypes during tumor growth and serial passaging [3-6]. Importantly, in our study, tumors derived from H3122/Ale cells remained insensitive to alectinib treatment (Fig. 7J), directly confirming that the resistant phenotype was preserved after implantation.

Regarding the dosing strategy, alectinib was administered at 20 mg/kg/day, a commonly used and pharmacologically active dose in preclinical xenograft studies [7]. In this context, the purpose of alectinib treatment was not to maintain resistance, but to provide a pharmacological reference for comparison, highlighting the limited efficacy of alectinib in resistant tumors while demonstrating that pFn+216@Trim21/αALK remains effective in this resistant setting.

Therefore, continuous alectinib administration prior to tumor establishment was not required for maintaining resistance, and the selected dosing regimen provides a relevant framework for evaluating treatment efficacy.

Reference

1. Wang D, Liu Y, Chen Y. *et al.* EGFR Targeted Liposomal PROTAC Assisted With Epigenetic Regulation as an Efficient Strategy for Osimertinib-Resistant Lung Cancer Therapy. *Adv Sci (Weinh).* **12**, e10197(2025).
2. Zhang L, He L, Lin Y. *et al.* The novel strategy to overcome the drug-resistant lung cancer: Dual targeting delivery of PROTAC to inhibit cancer-associated fibroblasts and lung cancer cells. *Drug Resist Updat.* **84**, 101316(2026).

3. Vasan, N., Baselga, J. & Hyman, D. M. A view on drug resistance in cancer. *Nature*. **575**, 299–309 (2019).
4. Gunnarsson EB, Magnússon BV, Foo J. Optimal dosing of anti-cancer treatment under drug-induced plasticity. *NPJ Syst Biol Appl.* **11**, 98(2025).
5. Marine, J.-C., Dawson, S.-J. & Dawson, M. A. Non-genetic mechanisms of therapeutic resistance in cancer. *Nat Rev Cancer.* **20**, 743–756 (2020).
6. Son, J. *et al.* AXL and SHC1 confer crizotinib resistance in patient-derived xenograft model of ALK-driven lung cancer. *iScience.* **27**, 110846 (2024).
7. Kleczko, E.K., Hinz, T.K., Nguyen, T.T. *et al.* Durable responses to alectinib in murine models of EML4-ALK lung cancer requires adaptive immunity. *npj Precis Onc.* **7**, 15 (2023).

2. There is a discrepancy between the description of drug administration in the Methods and the treatment groups shown in Figure 7. In the Methods, it appears that only the control group received oral alectinib, whereas Figure 7 indicates that alectinib was co-administered with other treatments in multiple groups. This inconsistency makes the experimental design difficult to interpret. The authors should clearly describe, for each group, which agents were given, at what dose, route, and schedule, and ensure that the text, Methods, and figures are fully consistent.
In Figure 7L, the six groups are: blank, alectinib only, alectinib + pFn+216, alectinib + Trim21/aALK, alectinib + Trim21/aALK + PULSin, and alectinib + Trim21/aALK + pFn+216. In Figure 7N, the six groups are: blank, alectinib only, pFn+216 only, alectinib + Trim21/aALK, alectinib + Trim21/aALK + PULSin, and alectinib + Trim21/aALK + pFn+216. These group definitions are not consistent between the panels, and it is unclear which design was actually implemented in the animal experiments. The authors need to clarify the exact composition of each group, correct any labeling errors, and, if necessary, revise the figures and legends so that the experimental design is unambiguous.

Response：

We thank the reviewer for carefully identifying this inconsistency. We apologize for the confusion caused by an error in figure labeling. Upon thorough re-examination of the Methods, figure panels, and legends, we confirm that the discrepancy arises from incorrect labeling in Fig. 7L and Fig. 7N. Specifically, alectinib was mistakenly indicated as being co-administered in multiple treatment groups in Fig. 7L and Fig. 7N, whereas in the actual experimental design, alectinib was administered only in the alectinib control group.

The correct treatment design is as follows:

- Saline group: no treatment
- Alectinib group: alectinib alone (20 mg/kg/day, oral gavage)

- pFn+216 group: pFn+216 alone
- Trim21/αALK group: Trim21/αALK complex alone
- Trim21/αALK@PULSin group
- Trim21/αALK @pFn+216 group

No alectinib was administered in any groups other than the alectinib control group. The incorrect inclusion of "+ Alectinib" in several groups in Fig. 7L and Fig. 7N was a labeling error introduced during figure preparation. We have now corrected the group labels in all relevant figure panels and revised the figure legends and Methods section to explicitly and consistently describe, for each group, the administered agents, doses, routes, and schedules.

Importantly, we would like to clarify that the description of drug administration in the Methods and Results section is accurate and reflects the actual experimental design. The inconsistency arose solely from incorrect labeling in specific figure panels (Fig. 7L and 7N) during figure preparation. This labeling error does not affect the experimental procedures, data interpretation, or the conclusions of the study. We have now carefully corrected the figure labels and ensured full consistency across the Methods, figure legends, and main text.

We sincerely apologize for this oversight and thank the reviewer for bringing it to our attention, which has helped us improve the clarity and accuracy of the manuscript. The revised parts are shown below (red colored in the text). The corresponding passages in the manuscript are marked in the same manner.

Manuscript Modification:

We would like to clarify that although the labeling errors occurred specifically in Fig. 7L and Fig. 7N, to further improve clarity of the experimental design, we have additionally revised the legend of the schematic diagram in Fig. 7H to explicitly define all treatment groups.

(H) Schematic diagram illustrating the antitumor effect in an H3122/Ale subcutaneous tumor model. Mice were randomly assigned to six groups (saline, Alectinib, pFn+216, Trim21/αALK, PULSin@Trim21/αALK, and pFn+216@Trim21/αALK).

3. Although the authors added some data to characterize alectinib resistance in the H3122/Ale cell line, the description in line 353 remains very limited: "Compared to the parental cells, H3122/Ale exhibited upregulation of the efflux transporters ABCB1 and ABCC1 (Extended Data Fig. 23A) and an approximately 16.2-fold increase in alectinib resistance." It is not explained why ABCB1 and ABCC1 were specifically examined, or what the mechanistic relationship is between these transporters and alectinib resistance. The authors should justify the focus on ABCB1 and ABCC1 with appropriate references (e.g., showing these transporters can limit ALK-TKI brain penetration or contribute to ALK-TKI efflux), and discuss how their upregulation mechanistically links to the observed resistance in H3122/Ale.

Response:

We thank the reviewer for this insightful comment and for highlighting the need for clearer justification. We would like to clarify that the primary objective of this study is to develop and validate an intracellular protein delivery platform, rather than to systematically dissect the mechanisms of alectinib resistance. Across multiple rounds of revision, we have incorporated additional experiments to strengthen the characterization of the resistant phenotype in response to the reviewer's suggestions; however, a comprehensive mechanistic investigation of resistance is beyond the scope of the present work.

ABCB1 and ABCC1 were selected as representative and well-established markers of drug efflux-associated multidrug resistance. These transporters are among the most extensively studied ATP-binding cassette (ABC) transporters and have been widely reported to reduce cellular sensitivity to a broad range of anticancer agents, including multiple tyrosine kinase inhibitors [1-4]. In our study, H3122/Ale cells exhibit significant upregulation of ABCB1 and ABCC1 (Extended Data Fig. 23A), together with an approximately 16.2-fold increase in alectinib IC_{50} (Extended Data Fig. 23B), indicating a markedly reduced drug sensitivity. While we do not claim a direct causal relationship, the elevated expression of these transporters is consistent with an efflux-associated resistance phenotype and may contribute to the observed reduction in drug responsiveness. We also note that acquired resistance is typically multifactorial and heterogeneous, particularly in models generated through long-term in vitro drug selection [5, 6]. Therefore, additional mechanisms may be involved, which are beyond the scope of the current study.

Importantly, we would like to further clarify the rationale for using the alectinib-resistant H3122/Ale model. In this study, the resistant tumor model was established to provide a stringent and clinically relevant context in which conventional ALK inhibitor therapy is ineffective, thereby enabling a more rigorous evaluation of the functional activity of the delivered protein cargo (Trim21/αALK). In this setting, the observed antitumor effects primarily reflect the successful intracellular delivery and activity of the protein-based degradation strategy, rather than the modulation of specific resistance mechanisms or the intrinsic properties of the delivery system itself.

Therefore, the detailed molecular basis of alectinib resistance is not expected to impact the central conclusion of this study, which is the development of an efficient ferritin-based nanocage system for cytosolic protein delivery. The revised parts are shown below (red colored in the text). The corresponding passages in the manuscript are marked in the same manner.

Manuscript Modification:

We have revised the Results section to provide clearer explanation:

Compared to the parental cells, H3122/Ale exhibited upregulation of the efflux transporters ABCB1 (P-glycoprotein) and ABCC1 (MRP1), which are ATP-binding cassette (ABC) transporters widely associated with multidrug resistance phenotypes in cancer cells[38-41]. Consistent with these observations, H3122/Ale cells displayed an approximately 16.2-fold increase in Alectinib resistance

compared to parental cells (Extended Data Fig. 23A-B), indicating a markedly reduced drug sensitivity. Functional assays confirmed a markedly attenuated response: while parental H3122 cells showed potent, dose-dependent inhibition at low Alectinib concentrations (1-100 nM), significant effects in H3122/Ale cells were only observed at the highest dose (100 nM) (Extended Data Fig. 23C-F).

Reference

1 Robey, R. W. et al. Revisiting the role of ABC transporters in multidrug-resistant cancer. *Nat Rev Cancer.* **18**, 452–464 (2018).

This review comprehensively documents the role of ABCB1 and ABCC1 in mediating resistance to chemotherapy and targeted therapies across multiple cancer types.

2. Shukla S, Chen ZS, Ambudkar SV. Tyrosine kinase inhibitors as modulators of ABC transporter-mediated drug resistance. *Drug Resist Updat.* **15**, 70-80(2012).

As noted in the literature: "Although a majority of the research in the area of clinical drug resistance to TKIs has been focused on identifying mutations in the target regions of kinases, resistance due to the overexpression of ABC drug transporters can still be a challenge. This should be studied as an alternative mechanism by which tumor cells evade the cytotoxic effects of TKIs."

3. Katayama R, Sakashita T, Yanagitani N. *et al.* P-glycoprotein Mediates Ceritinib Resistance in Anaplastic Lymphoma Kinase-rearranged Non-small Cell Lung Cancer. *EBioMedicine.* **3**, 54-66(2015).

4. Wu Z, Zhao W, Yang Z, Wang YM, Dai Y, Chen LA. Novel Resistance Mechanisms to Osimertinib Analysed by Whole-Exome Sequencing in Non-Small Cell Lung Cancer. *Cancer Manag Res*. **13**, 2025-2032(2021).

As noted in the literature: "ABCC1 in the ABC membrane pump system were identified as candidate crucial genes of drug resistance to osimertinib."

5. Vander Velde, R., Yoon, N., Marusyk, V. *et al.* Resistance to targeted therapies as a multifactorial, gradual adaptation to inhibitor specific selective pressures. *Nat Commun.* **11**, 2393 (2020).

6. Marusyk A, Janiszewska M, Polyak K. Intratumor Heterogeneity: The Rosetta Stone of Therapy Resistance. *Cancer Cell.* **37**, 471-484(2020).

4. Regarding the question of whether the antitumor effect depends on EML4-ALK degradation, the current dose-response experiments are not sufficient to resolve this point. To rigorously test degradation-dependence, the authors should include a control group in which the same amount of complex (e.g., Trim21/aALK + carrier) is administered, but EML4-ALK degradation is selectively blocked (for example, by inhibiting the degradation machinery or using a degradation-defective construct), and then compare antitumor efficacy. Without such

a degradation-blocked control, it remains unclear to what extent the observed antitumor effects truly depend on EML4-ALK degradation versus other mechanisms.

Response:

We thank the reviewer for this critical question regarding whether the observed antitumor effect depends on EML4-ALK degradation. To directly address this point, we employed a loss-of-function approach using a catalytically inactive Trim21 mutant. Specifically, we introduced the Trim21(C16A) mutant, which carries a point mutation in the RING domain that disrupts E3 ubiquitin ligase activity [1]. Using this strategy, we compared the effects of wild-type Trim21 and Trim21(C16A) in H3122/Ale cells (Extended Data Fig. 27). We found that the catalytically inactive mutant abolished EML4-ALK degradation, demonstrating that the degradation process depends on the ubiquitination activity of Trim21. These results support that the observed target reduction is mediated through a Trim21-dependent proteasomal mechanism.

In addition, we would like to note that appropriate controls to distinguish delivery effects from cargo function were included in our original experimental design (Fig.7F-N). In particular, treatment with the delivery carrier alone (e.g., pFn+216) did not produce significant antitumor effects, indicating that the therapeutic activity is not attributable to the carrier itself, but rather to the functional protein cargo. We also note that, in previous rounds of revision, we have incorporated additional supporting experiments, including pharmacological inhibition assays (Extended Data Fig. 24) and in vivo dose-response studies (Extended Data Fig. 26), to further strengthen the mechanistic interpretation.

We agree that a fully systematic dissection of all possible degradation pathways would provide additional insight; however, this is beyond the primary scope of the present study, which focuses on the development of an efficient intracellular protein delivery platform. Nevertheless, in response to the reviewer's suggestion, we have now included the Trim21(C16A) loss-of-function experiment to more directly demonstrate the dependence of target degradation on Trim21 activity. The supplementary experimental data are marked (green colored in the bracket), and revised parts are shown below (red colored in the text). The corresponding passages in the manuscript are marked in the same manner.

Manuscript Modification:

We have added the Trim21(C16A) mutant experimental data as **Extended Data Figure 27** to the Supplementary Materials. The Results and sections have been revised to incorporate these findings:

To further assess whether these effects depend on Trim21 catalytic activity, we employed the catalytically inactive Trim21(C16A) mutant[35]. In H3122/Ale cells, while pFn+216@Trim21/αALK effectively reduced EML4-ALK levels, the C16A mutant largely abrogated this effect (Extended Data Fig. 27A), indicating that the degradation process depends

on Trim21 catalytic function. The C16A mutant failed to suppress cell viability or inhibit clonogenic survival, whereas wild-type Trim21 retained these effects (Extended Data Fig. 27B-C). Importantly, in vivo validation using H3122/Ale xenografts showed that the C16A mutant lost tumor-suppressive activity, while wild-type Trim21 significantly inhibited tumor growth (Extended Data Fig. 27D-F). These results demonstrate that Trim21 catalytic activity is required for efficient EML4-ALK degradation and is closely associated with the observed antitumor effects.

Reference

1. Li JY, Zhao Y, Gong S. *et al.* TRIM21 inhibits irradiation-induced mitochondrial DNA release and impairs antitumour immunity in nasopharyngeal carcinoma tumour models. *Nat Commun.* **14**, 865(2023).

5. For every figure, the legend should clearly specify the statistical analysis used and the comparison strategy (e.g., which groups were compared, one-way vs two-way ANOVA, post hoc tests, exact n, and definition of error bars). This is standard good practice for scientific figures and is necessary for readers to correctly interpret the results. The authors should revise all figure legends accordingly.

Response:

We thank the reviewer for this important comment. In response, we have comprehensively revised both the Statistical Analysis section and all figure legends to clearly specify the statistical methods used and the comparison strategies for each dataset. For each figure, we now explicitly indicate the statistical test applied (e.g., unpaired two-tailed Student's t-test, one-way or two-way ANOVA), the corresponding multiple comparison method (Dunnett's, Tukey's, or Šídák's), the exact sample size (n), and the definition of error bars. The revised parts are highlighted in red in the manuscript.

Thank you!

REVIEWERS' COMMENTS

Reviewer #1 (Remarks to the Author):

1. **In Figure 2C, it is not clear what the blot represents. Is the detection antibody against Fn, or something else? The authors should state this clearly.**

Response:

The reviewer is referring to Supplementary Fig. 2C rather than Fig. 2C in the main text. This figure shows a western blot analysis performed for protein identification purposes, and the blot was detected using an anti-Fn antibody. To clarify this point, we have revised the figure legend in the revised manuscript as follows: "Supplementary Fig. 2C: Western blot analysis for protein identification. The PVDF membrane was stained with anti-Fn antibody to confirm the identity of ferritin protein."

2. **The published reference (PMID: 29153837) clearly suggests that endogenous TRIM21 can mediate protein degradation. As expected in Figure S22E, the GFP level is significantly decreased compared with the PBS and IgG groups; however, in Figures S22B, S22C, and S22D, the results appear to be inconsistent with Figure S22E. Please clarify why this discrepancy occurs.**

Response:

We thank the reviewer for this valuable comment. Due to figure reorganization during revision, the apparent discrepancy is between Supplementary Fig. 22D (corresponding to Fig. S22E in the reviewer's comment) and Supplementary Fig. 22A-C (corresponding to Fig. S22B-D in the reviewer's comment), which may arise from differences in the detection methods used. Confocal imaging (Supplementary Fig. 22A) and flow cytometry (Supplementary Fig. 22B-C) measure GFP fluorescence signals, whereas western blotting (Supplementary Fig. 22D) directly detects GFP protein levels. Therefore, changes in GFP fluorescence intensity may not fully parallel the reduction in protein abundance detected by western blotting. Importantly, the results from all three methods support the same overall conclusion. In particular, the C16A catalytic mutant of Trim21 showed markedly reduced degradation activity compared with wild-type Trim21, indicating that GFP reduction is dependent on TRIM21 activity. Together with the other experimental results, these findings support that the pFn+216@Trim21/αGFP complex effectively mediates GFP degradation through the TRIM21-proteasome pathway.

3. **<u>In Figures S23E and S23G, the gating strategy appears to differ, even in the PBS group. For similar experiments, the same gating settings are recommended so that the data can be compared more reliably.</u>**

Response:

We thank the reviewer for this valuable technical suggestion. Upon careful review, we found that the gating strategies used in Figures S23E and S23G were highly similar, although not completely identical in the original presentation. We agree that using the same gating settings would allow more direct comparison between experiments. Therefore, we re-analyzed the flow cytometry data using identical gating parameters and updated the figures accordingly. The re-analysis yielded results consistent with our original conclusions.

4. **<u>In Figure 7M, what is the percentage of TUNEL-positive cells in the randomly selected fields? It appears to be nearly 100% positive. Such a high level often suggests extensive necrosis or technical artifacts rather than pure apoptosis, especially given the large difference between the pFn+216@TRIM21/αALK group and the control groups.</u>**

Response:

We thank the reviewer for this important comment. The representative image shown in Fig. 7M may give the impression of very high TUNEL positivity due to the strong apoptotic response observed in the pFn+216@TRIM21/αALK treatment group. However, quantitative analysis was performed using 3 randomly selected fields per slide from 5 animals per group, as shown in Fig. 7N. The quantified percentage of TUNEL-positive cells was significantly increased in the pFn+216@TRIM21/αALK group compared with all control groups, but was not close to 100%.

The marked increase in apoptosis is consistent with the mechanism of the TRIM21/αALK-mediated targeted degradation system, in which effective ALK degradation induces tumor cell apoptosis specifically in the pFn+216@TRIM21/αALK group. We have updated the figure legend to clarify the quantification method and sample size.

5. **<u>In Figure 7I, what does “CR” mean? The authors should define it clearly in the text.</u>**

Response:

We apologize for the omission and thank the reviewer for pointing this out. “CR” stands for Complete Response. We have revised the figure legend to clearly define this abbreviation. The updated legend now reads: “Fig.7I: Tumor growth curves of different treatment groups over 21

days. CR, Complete Response, defined as tumor volume less than 50 mm^3 for the remainder of the experiment."

6. **<u>In Figure 7, it is also recommended to measure the p-AKT/AKT ratio and Bcl-2 expression levels across the different groups.</u>**

Response:

We greatly appreciate this constructive suggestion. We performed additional western blot analyses of tumor tissue lysates to evaluate the p-AKT/AKT ratio and Bcl-2 expression levels. The results showed significant reductions in both the p-AKT/AKT ratio and Bcl-2 expression in the pFn+216@TRIM21/αALK treatment group. The representative western blot images are shown in Supplementary Fig. 27G, and the quantitative analysis is presented in Fig. 7L.

These findings further support that pFn+216@TRIM21/αALK treatment suppresses AKT signaling and promotes apoptosis in vivo. Accordingly, we have added the following description to the Results section: "Mechanistically, Western blot analysis confirmed the effective depletion of EML4-ALK fusion protein *in vivo* (Fig. 7L and Supplementary Fig. 27G). Consequently, we observed a marked inactivation of the AKT signaling pathway, reflected by a significant reduction in the p-AKT/AKT ratio ($P< 0.001$). This was accompanied by a significant downregulation of the anti-apoptotic protein Bcl-2 ($P< 0.001$)."

Thank you!
